# One Pot Synthesis of the C-3 Complex (Curcumin, Demethoxycurcumin, and Bis-Demethoxycurcumin): Their Joint and Independent Biological Actions

**DOI:** 10.3390/ijms26199599

**Published:** 2025-10-01

**Authors:** Marco A. Obregón-Mendoza, Rubén Sánchez-Obregón, Rosario Tavera-Hernández, Leidys L. Pérez-González, Antonio Nieto-Camacho, Rogelio Rodríguez-Sotres, Carolina Escobedo-Martínez, Irma Romero, Raúl G. Enríquez

**Affiliations:** 1 Instituto de Química, Universidad Nacional Autónoma de México, Circuito Exterior, Ciudad Universitaria, Ciudad de México 04510, Mexico; marco.obregon@zaragoza.unam.mx (M.A.O.-M.); rubens@unam.mx (R.S.-O.); rosario.tavera@gmail.com (R.T.-H.); leidyslaura92@gmail.com (L.L.P.-G.); camanico2015@yahoo.com (A.N.-C.); 2 Facultad de Medicina, Universidad Nacional Autónoma de México, Ciudad de México 04510, Mexico; sotres@unam.mx; 3 Departamento de Farmacia, División de Ciencias Naturales y Exactas, Universidad de Guanajuato, Campus Guanajuato, Guanajuato 36050, Mexico; c.escobedo@ugto.mx; 4 Departamento de Bioquímica, Facultad de Medicina, Universidad Nacional Autónoma de México, Ciudad Universitaria, Ciudad de México 04510, Mexico; irma@bq.unam.mx

**Keywords:** *Curcuma longa*, C-3 complex, curcumin, demethoxycurcumin, bis-demethoxycurcumin, DPPH scavenging, TBARS, antioxidant

## Abstract

Curcumin (CUR) is the primary metabolite isolated from the *Curcuma longa* L. rhizome. Most synthetic and biological studies have focused mainly on the curcumin molecule due to its essential biological activity as an antioxidant, anti-cancer, and anti-Alzheimer’s disease agent. However, the natural extract of turmeric also contains two essential curcuminoids (demethoxycurcumin (DMC) and bisdemethoxycurcumin (BDMC)), which altogether comprise the so-called C-3 complex. They are present in commercial compositions for treating biliary or digestive ailments. The vegetal rhizome’s extraction typically leads to a mixture of the three main curcuminoids, CUR, DMC, and BDMC, in variable proportions, and each of these metabolites has reported specific synthetic routes. Herein, we have performed the synthesis and isolation of the three major curcuminoids using the method called scrambling of aldehydes followed by aldol di-condensation reactions. A density functional theory (DFT) approach supported the experimental results by inspecting the predicted energies for the aldol condensation. Thus, the di-condensation reaction is substantially favoured (*ΔG*° = −2685.9 kJ/mol) over the mono-condensation reaction (*ΔG*° = −1393.753 kJ/mol). Our approach allows us to mimic closely the proportions of these curcuminoids found in extracts from natural sources that follow the order CUR > DMC > BDMC, respectively. The proportion of aldehydes can be modified in the scrambling reaction with an adequate mixture of aldehydes to render the order DMC > CUR > BDMC. This is an advantageous way to increase the amount of the unsymmetric DMC metabolite.

## 1. Introduction

**Figure 1:**
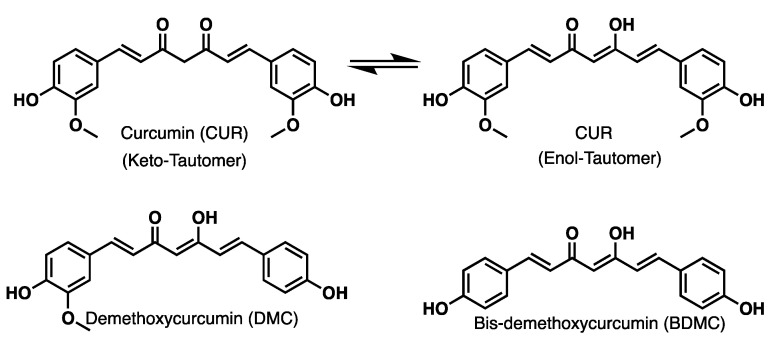
Main curcuminoids extract from turmeric.

Turmeric, the natural E100 dye [1], belongs to the Zingiberaceae family [2,3]. Turmeric rhizome contains three secondary metabolites [4], namely the following curcuminoids [5]: curcumin (CUR), demethoxycurcumin (DMC), and bis-demethoxycurcumin (BDMC) [6,7], with structures shown in Figure 1. They are extracted from the natural source as a mixture and receive the name C-3 complex [8]. They are also considered the primary active metabolites of turmeric extract. However, both total curcuminoid content and individual ones in the rhizome depend on the region where they are harvested and from the plant genotype [9].

*Curcuma longa* has an overall content of 5.80% m/m of curcuminoids when the extract originates from India, while the same species harvested from Jamaica contains a total of 3.10% m/m of curcuminoids [10]. Different species of *Curcuma* sp. harvested from Indonesia have curcuminoid contents of 2.9–9.1% for *C. domestica*, 0.8–1.0% contents for *C. zanthorrhiza*, and 0.02–0.03% for *C. aeruginosa* [9]. Moreover, the average content of individual curcuminoids for India’s turmeric is [5] as follows: 75–80% for curcumin, 15–20% for demethoxycurcumin, and 3–5% for bis-demethoxycurcumin.

In addition to the variability of genotypes [11] and growing conditions [12], the damage to rhizomes during harvest must be avoided, because of the presence of pathogens [13]. Therefore, the management of this crop is considered difficult [14]. Although in vitro propagation options have addressed these problems, a high variability of curcuminoid percentages occur.

Most scientific studies are focused on the main metabolite curcumin [15] and its derivatives, although studies involving the three metabolites [16] are found in the literature to a lesser degree. In the latter case, the regular trend is that the proportions of metabolites follow the content order [16,17,18,19,20,21]: curcumin (CUR) > demethoxycurcumin (DMC) > bisdemethoxycurcumin (BDMC). It is important to emphasise that most turmeric’s therapeutic properties are associated with these three metabolites [22].

It is known that turmeric extract has extensive medicinal properties and is used to treat bile problems, abdominal pain, and icterus, among others. In addition, turmeric has essential therapeutic properties such as being an antioxidant [23], an anti-cancer [24], and an anti-Alzheimer’s disease agent [25,26]. Therefore, it is essential to emphasise that these activities have been awarded to the three main curcuminoids (chemically diarylheptanoids, Figure 1). The extraction process of curcuminoids by chromatographic [18] or fractional crystallisation methods limits the production of individual chemical components [27]. Moreover, the analysis and isolation of curcuminoids from turmeric is carried out mainly by chromatographic techniques. Although high-performance liquid chromatography (HPLC) delivers good efficiency in separating chemical components from the rhizome extract, the need for high-purity solvents makes it a rather expensive procedure [28]. Therefore, it is convenient to develop a synthetic process to obtain these three molecules either as individual compounds or as a mixture (C-3).

The underlying idea of the present work is the synthesis, through a simple reaction, of the curcuminoids present in the natural source.

## 2. Results

Herein, we obtained CUR, DMC, and BDMC individually through synthetic procedures. Secondly, to achieve a mixture in a whole (mixture C-3), a scrambling of aldehydes (e.g., vanillin and 4-hydroxybenzaldehyde) was used and condensed with the difluoroboronate of acetylacetone (synthon 1, see ^1^H NMR in Figure S1). Three experimental conditions of different proportions of aldehydes allowed the obtention of mixtures with different chemical ratios of curcuminoids C-3. Furthermore, all curcuminoids are analysed by their ability to scavenge free radicals of 2,2-diphenyl-1-picrylhydrazyl (DPPH) and thiobarbituric acid reactive substances (TBARS) and were compared to each other.

The synthesis of C-3 is achieved by varying proportions of the reagents used. In contrast, the natural source is affected by the quality of the processed plant rhizome and the solvent extraction method employed. As a result, synthetic compounds are devoid of pathogens and other components typically found in turmeric rhizomes, such as essential oils, fats, sugars, and fibres.

### 2.1. Synthesis of Symmetric Curcuminoids Through Acetyl Aldehydes

**Figure 2:**
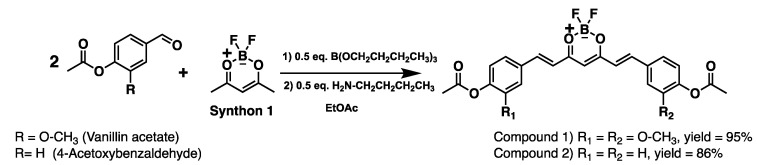
Synthesis of di-fluoroboron curcuminoids symmetric (precursors).

Two equivalents of acetylated aldehydes (vanillin acetate or 4-acetoxybenzaldehyde, see ^1^H NMR in Figures S2 and S3) were condensed, respectively, with one equivalent of difluoroboron-pentanedione (synthon 1) for the precursor’s synthesis of curcumin or bis-demethoxycurcumin (symmetrical curcuminoids), as depicted in Figure 2.

The yields of curcuminoids-BF_2_ are excellent (up > 86%, for characterisation spectral see Figures S4–S16) due to the blocking of phenols (from starting aldehydes) with acetyl groups, which allowed us an increase of 5% of the overall yield of symmetrical curcuminoids compared to previously reported yields [29]. It is possible that the catalyst (n-butylamine), responsible for generating the nucleophile (enolate), becomes more efficient when phenolic groups are protected.

**Figure 3:**
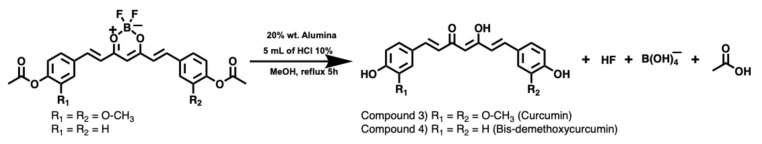
Symmetrical curcuminoids synthesis.

Then, after removal of the difluoroboron (BF_2_) moiety and acetyl groups from the intermediate precursors, the symmetrical diarylheptanoids were obtained in enolic form [30], as shown in Figure 3.

The recovery of the beta-diketone functionality was carried out using alumina and HCl as catalysts (for spectral characterisation, see Figures S17–S23 (Curcumin), Figures S24–S30 (Bis-demethoxycurcumin) and Figures S31–S37 (Demethoxycurcumin), respectively). This approach allowed obtaining the curcuminoids in relatively short reaction times (5 h), because the constant generation of acetic acid in the reaction medium (from hydrolysis of the acetyl group) contributes to the elimination of the BF_2_ group.

### 2.2. Synthesis of Mixtures C-3 (CUR, DMC, and BDMC)

**Figure 4:**
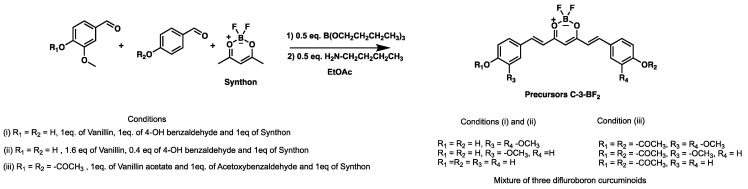
Synthesis of C-3 curcuminoids-BF_2_ (Precursors).

In this research, we used three different experimental conditions to obtain mixed curcuminoids in the form of complexes (C-3) as follows: (i) scrambling 1 eq of each aldehyde (vanillin and 4-hydroxybenzaldehyde), followed by di-condensation with 1 eq of the synthon; (ii) using disproportionation in the scrambling of aldehydes, i.e., 1.6 eq of vanillin and 0.4 eq of 4-hydroxybenzaldehyde, followed by di-condensation with 1 eq of the synthon; (iii) and using the scrambling of 1 eq of each acetylated aldehyde (namely acetyl vanillin and 4-acetylbenzaldehyde), followed by di-condensation with 1 eq of the synthon (see Figure 4).

The mixture of the three intermediate precursors for the synthesis of C3-BF_2_ was isolated using a one-pot approach [30] with minimal workup. In all cases, precipitated powders were obtained in the reaction medium (NMR spectra, infrared and mass spectrometry for the C3-BF_2_ precursors are found in Figures S38–S49). They were filtered off, and at the end of the isolation process, the bulk weight of the material reached an overall yield of 90%.

Afterwards, BF_2_ group was removed by acid hydrolysis [31], as depicted in Figure 5, and afforded the curcuminoids in enolic form in different chemical proportions (analysed by HPLC) for each experimental condition (i, ii, and iii). Spectral characterisations of the C-3 curcuminoid mixtures are found in Figures S50–S61.

## 3. Discussion

There are three curcuminoids present in the *Curcuma longa* L. rhizome [32]; two of them are symmetrical (CUR and BDMC) and one is unsymmetrical (DMC) [33]. Using this synthetic approach, the obtention of symmetrical curcuminoids is possible since the BF_2_-type precursor allows obtaining consistently high yields [34] and they are presented as precipitated solid powders (by reacting 2 moles of the same aldehyde with one mole of the synthon, 1:0.5 ratio). Although an attempt was made to synthesise the asymmetric curcuminoid (DMC) in pure form by an aldol di-condensation reaction by mixing two different aldehydes with the synthon (in molar proportions 1:1:1), this was not possible due to competitive reactions between the other two possible symmetric curcuminoids obtained in the reaction flask [35].

Another synthetic option for obtaining the unsymmetric curcuminoids is the isolation of a monocondensed product (i.e., reacting an aromatic aldehyde with the synthon using the 1:1 ratio). The second step occurs by condensation with a second aldehyde. However, in this way, very low yields have been reported [36], while in extreme cases, the second condensation does not occur.

**Figure 6:**
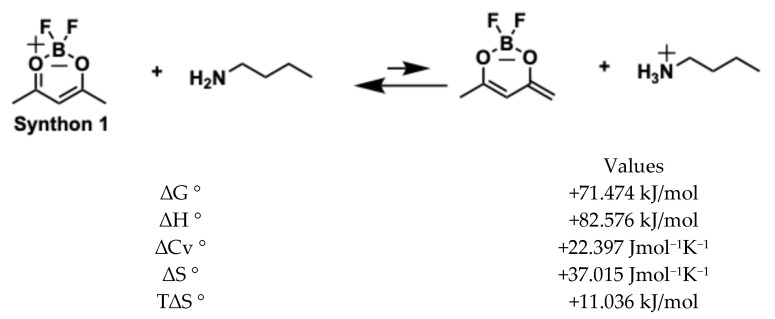

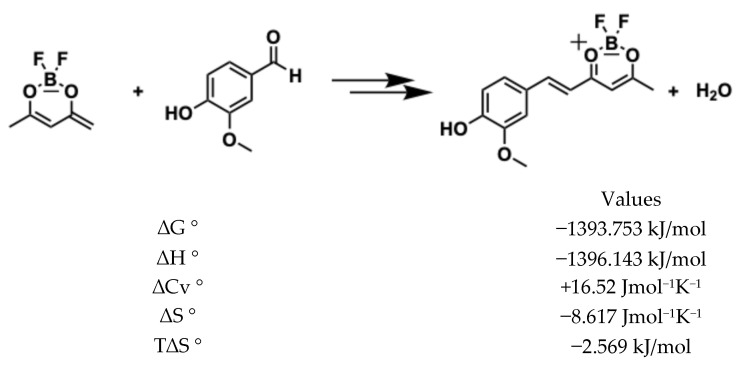
Generation of enolate and first condensation of vanillin.

Therefore, we have performed theoretical calculations to give a plausible explanation in the formation of mono- and di-condensed products, and we have taken as an example the condensation reaction of vanillin with the synthon, as shown below:

The reaction of difluoroboronite of acetylacetone (synthon 1) with vanillin, catalysed by n-butylamine, occurs in two steps: first, synthon 1 donates an acidic proton to n-butylamine; secondly, the resulting anion acts as a nucleophile to attack the aldehyde group of vanillin (see Figure 6). According to thermochemical calculations (DFT: B3LyP basis: aug-cc-pvdz), the anion formation is slightly unfavourable (in the first step), having a positive *ΔG*° = +71.4 kJ/mol. However, the first reaction of nucleophilic substitution (monoaldolic condensation) is highly favourable and drives the whole reaction forward with a global result of *ΔG*° = −1393.753 kJ/mol.

**Figure 7:**
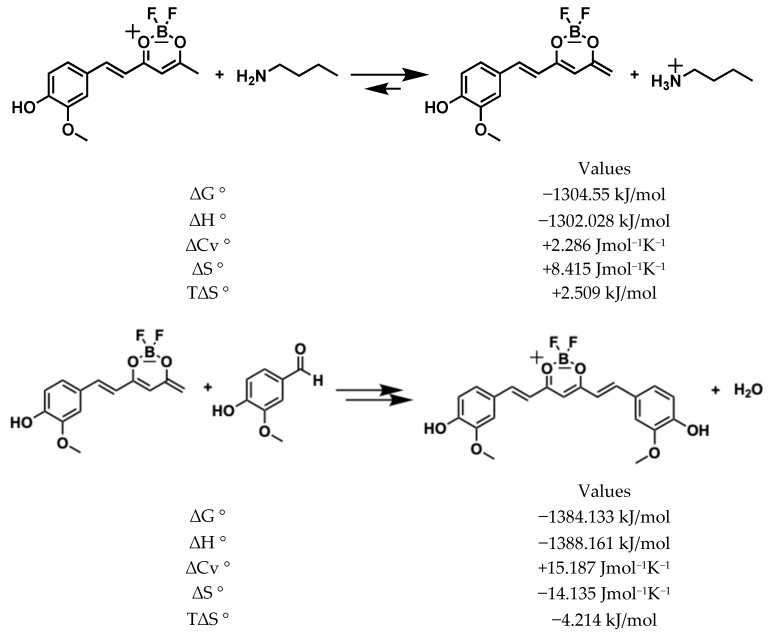
Generation of second enolate and second condensation of vanillin with mono condensed synthon.

To produce a di-condensed compound, the process must repeat itself and the product of the synthon 1 mono-condensed must donate a second acid proton. Unlike the first enolization, the negative charge in this step can be distributed in the aromatic ring, and the free energy is very favourable (*ΔG*° = −1304.5 kJ/mol), as shown in Figure 7. Hence, the second nucleophilic attack (di-condensation aldolic) to the aldehyde group of vanillin is as favourable (*ΔG*° = −1384.13 kJ/mol) as the first substitution, and the global *ΔG*° for the two reactions is highly favourable (*ΔG*° = −2685.9 kJ/mol).

This analysis does not hint at the relative rates of each reaction, because the structure of the corresponding transition states is uncertain, as the aldol condensation does not occur in a single step. Still, it takes the nucleophilic attack of an anion to the carbonylic carbon, followed by water elimination and a new double bond generation. However, the values can be used to predict the number of chemical species at equilibrium. So, it is expected that under this synthetic approach, the main products at the end of the reaction will correspond to di-condensate products. From this analysis, it follows that when vanillin and synthon 1 react in a 1:1 mol ratio, the di-substituted species will still predominate in the final products. A part of the mono-condensed product in low concentration remains in the reaction flask.

Although the theoretical study answers that the final product will always be a di-substituted curcuminoid, the experimental and analytical parts help to predict the proportions of curcuminoids that will be obtained when scrambling is carried out with chemically different aldehydes.

**Figure 8:**
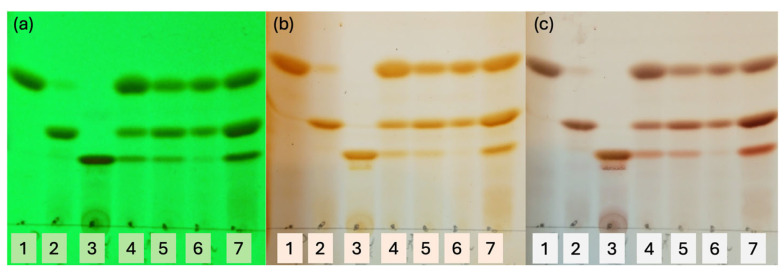
TLC with solvents CH_2_Cl_2_/Hexane/Methanol 65::30::5, three times of elution. (**a**) Under lamp UV 253 nm, (**b**) iodine chamber, (**c**) cerium (IV) sulphate. Spots: 1 = CUR, 2 = DMC, 3 = BDCM, 4 = extract from turmeric (Sigma-Aldrich), 5 = C-3 experimental condition (i), 6 = C-3 experimental condition (ii), 7 = C-3 experimental condition (iii).

We verified the obtention of these three curcuminoids by thin-layer chromatography (TLC). Furthermore, an improved resolution of the mixture of curcuminoids was achieved in TLC using a combination of three solvents, as shown in Figure 8.

We have used pure standards of CUR, DMC, and BDMC in the first three spots of the TLC to compare with the following spots that correspond to turmeric extract and curcuminoids of the three experimental conditions carried out.

Analysing the lanes for spots 5, 6, and 7, a greater intensity of CUR (spot 6) is observed only in the experimental conditions (ii). These results suggest that the chemical function of aldehydes (phenol or acetyl) is not influencing the final concentrations of curcuminoids because almost the same concentration was obtained when HPLC analysed the samples following the order DMC > CUR > BDMC for experimental conditions (i) and (iii).

When a scrambling of aldehydes is used in the relationships 1:1 (conditions (i) and (iii)), a higher concentration enriched with DMC is obtained, but when an 80::20 proportion of aldehydes is used as in the experimental condition (ii), CUR is obtained in the highest concentration. Surprisingly, in all cases, a lower concentration of BDMC has been obtained. However, it is enriched when acetylated aldehydes were used for experimental condition (iii) and is shown in spot 7 (TLC).

**Figure 9:**
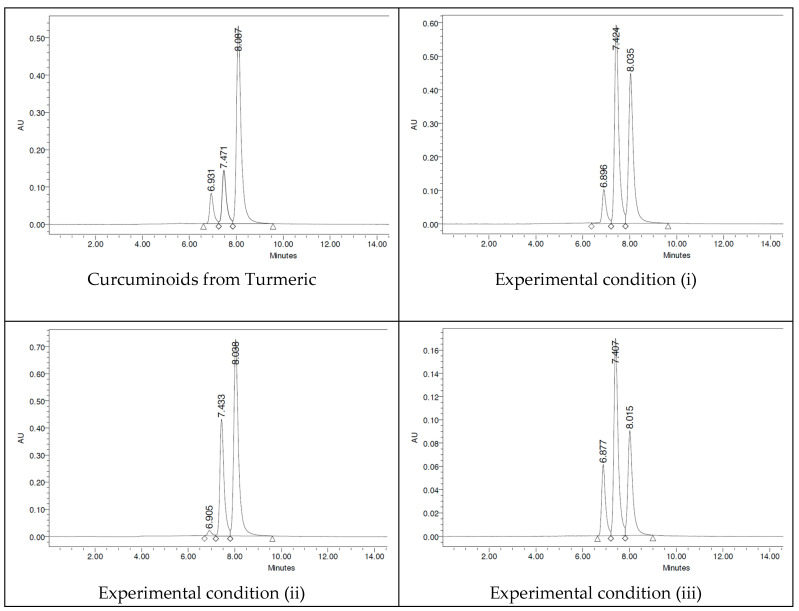
HPLC chromatograms for C-3 curcuminoid mixtures.

The HPLC chromatograms were carried out with a mixture of three solvents (acetonitrile/water/orthophosphoric acid (0.02%)). In the HPLC studies, it can be observed that synthetic curcuminoids in the form of mixtures show the exact retention times as the turmeric curcuminoid extract, occurring naturally. Indeed, synthetic samples adequately cover all aspects of the curcuminoids of natural origin. The HPLC chromatograms presented in Figure 9 show that the retention times of CUR (8 min), DMC (7.4 min), and BDMC (6.9 min) are the same for all samples studied.

The percentage amounts of curcuminoids are reported in Table 1, where the concentrations of curcuminoids obtained correlate appropriately with the TLC study. The concentrations of synthetic curcuminoids obtained in experimental condition (ii) mimic those of turmeric rhizome [21,22] (i.e., CUR > DMC > BDMC), and this experimental condition (iii) shows enrichment of the unsymmetric curcuminoid (DMC). The mention above is brought out because we used a synthetic source of DMC, obtained by preparative TLCchromatography.

The above has led us to suggest that it is possible to obtain the curcuminoids present in turmeric rhizome by synthetic procedures and maintain practically the same chemical relationships, resulting in the highest concentration for CUR [37], an intermediate concentration for DMC and the lowest concentration for BDMC. The synthetic procedure indicates that aldehyde scrambling could be performed similarly to experimental conditions (ii) (i.e., disproportionally), which could result in mimicry of natural curcuminoid concentrations.

NMR spectroscopy was carried out on both purified precursors (curcuminoids-BF_2_) and all curcuminoids of the synthetic route. The analysis of the BF_2_ precursors allowed us to establish that the chemical shifts for vinylic and central methine protons are in major frequencies with respect to target curcuminoids due to the electron-withdrawing effect of the BF_2_ moiety.

**Figure 10:**
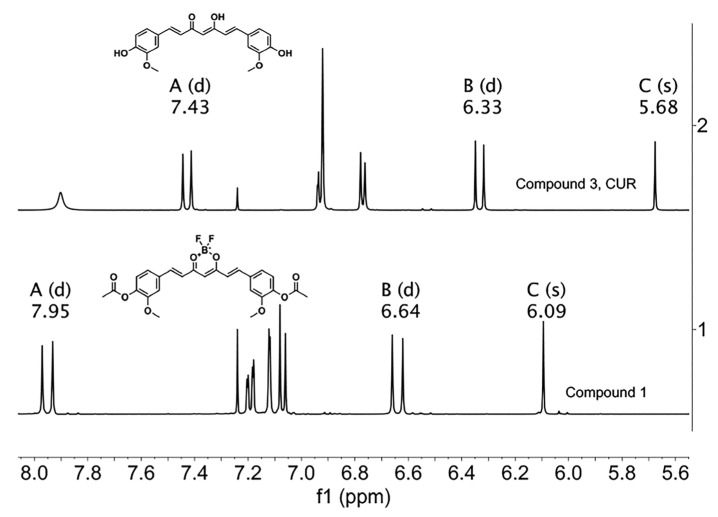
^1^H-NMR spectra of precursor **1** and CUR. The letters A, B and C correspond to ββ, αα, and C methine protons, respectively. (CDCl_3_, 400 MHz).

The ^1^H-NMR spectrum of the BF_2_ precursor shows chemical shifts to higher frequencies with respect to the CUR [29]. Methine protons occur at 6.09 ppm for precursor (compound **1**) and 5.09 ppm for CUR (compound **3**). The doublet that corresponds to the vinyl protons of the precursor at 7.95 ppm (ββ) and 6.64 ppm (αα) showed higher shifts, whereas the vinyl protons of CUR showed lower shifts at 7.43 ppm (ββ) and 6.33 ppm (αα), as depicted in Figure 10.

**Figure 11:**
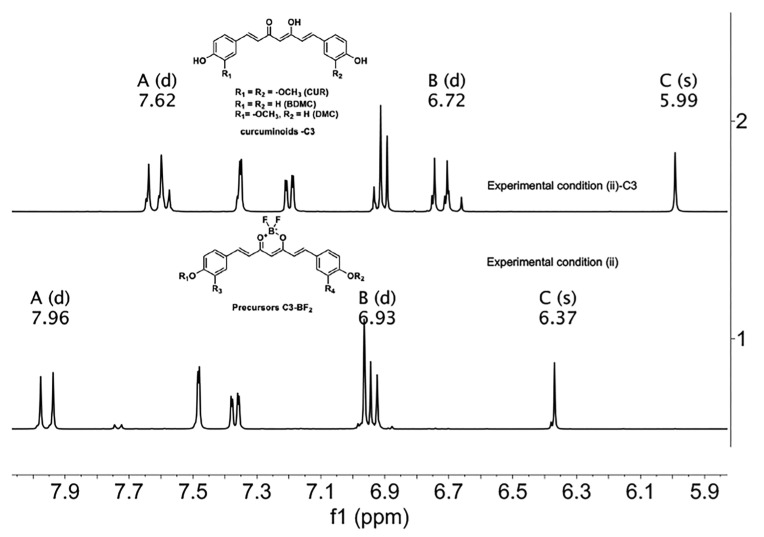
^1^H-NMR spectra of (1) C-3 precursors-BF_2_ and (2) C-3 Curcuminoids from experimental condition (ii). The letters A, B and C correspond to ββ, αα, and C methine protons, respectively. (Acetone-*d_6_*, 400 MHz).

The previous analysis has served as the criterion to establish a follow-up by spectroscopic means from curcuminoids-BF_2_ (precursors) to curcuminoids in C-3 mixtures. Although the ^1^H-NMR spectra of the BF_2_ precursors and curcuminoids are not easy to assign because they are mixtures (superposition of signals) of compounds in different proportions, some regions of the spectrum are susceptible to analysis. For example, the protons in the aromatic region (6.5 ppm–7.7 ppm, for precursors-BF_2_) are observed with fewer signals because of the overlap of signals in the mixture. Curcuminoids exhibit several sets of signals in the same region. Furthermore, methine protons can be identified as singlets at 6.37 ppm (high frequencies) for precursors-BF_2_ and 5.99 ppm (low frequencies) for curcuminoids C-3. Double signals of the vinylic protons are differentiated at 7.96 ppm (ββ) and 6.93 ppm (αα) for the precursors of BF_2_, while curcuminoid-C-3 has lower chemical shifts with respect to the starting materials with signals at 7.62 ppm (ββ) and 6.72 ppm (αα), as shown in Figure 11.

Although CUR and turmeric curcuminoids often share the same commercial name “curcumin” [38], their chemical composition of individual components is different. Therefore, to establish a difference between curcumin (CUR) and curcuminoid mixtures from turmeric or synthetic C-3 (CUR, DMC, BDMC) is essential. Thus, we recorded and compared their ^1^H-NMR spectra in Acetone-*d_6_*.

The CUR molecule shows one set of perfectly differentiated signals [39]. The singlet signal at 5.98 ppm corresponds to the methine proton, and the vinylic protons at 7.60 ppm (ββ) and 6.70 ppm (αα) are observed as doublets. In comparison, the aromatic protons are observed with their respective splits at 6.88 ppm (doublet, ortho), 7.17 ppm (doublet of doublet, ortho and meta), and 7.33 ppm (doublet, meta); the singlets for methoxy groups are not shown in the spectrum but correspond to the signals at ca. δ3.9 ppm (see Supplementary Materials).

The NMR spectrum at 400 MHz in Acetone-*d_6_* does not distinguish the three different protons expected for each synthetic curcuminoid, at ca. δ5.98 ppm for the central methines. Furthermore, signals of aromatic proton in regions (6.6 ppm–7.7 ppm) correspond to the mixture of the three curcuminoids, while signals for vinyl’s protons (ββ) and (αα) appear at 7.60 ppm and 6.70 ppm, respectively.

**Figure 12:**
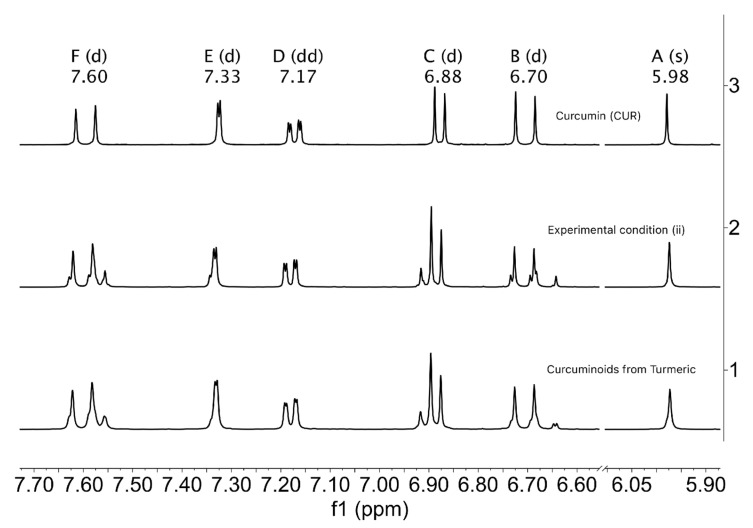
^1^H-NMR spectra of aromatic regions of curcuminoids: 1, curcuminoids from natural turmeric; 2: mixture of curcuminoids from experimental condition (ii); and 3: synthetic curcumin, **3**. The regions marked with letters correspond to: F to ββ, E, D and C to aromatics, B to αα, and A to methine protons for the three curcuminoids, respectively. (Acetone-*d_6_*, 400 MHz).

The resulting spectra show distinctive signals for CUR in all cases, while other signals exhibit some degree of overlap, as can be seen in Figure 12.

**Figure 13:**
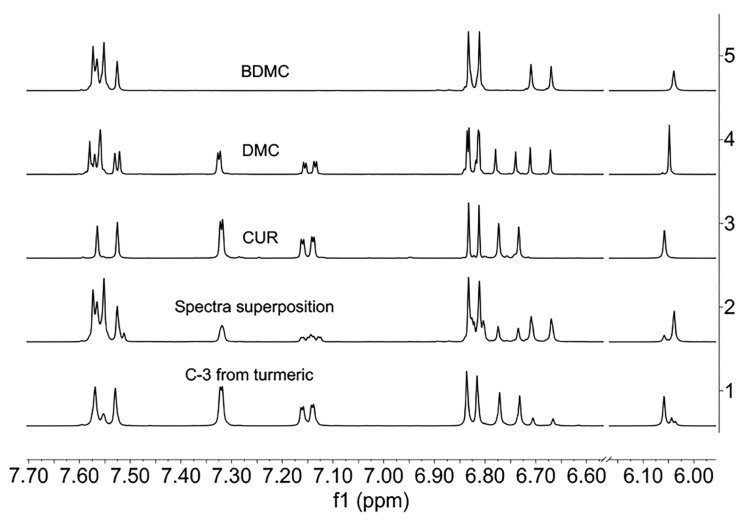
Spectra of curcuminoids 1: from turmeric; 2: superposition; 3: CUR; 4: DMC; and 5: BDMC (^1^H-NMR, aromatic region, DMSO-*d_6_*, 400 MHz).

We carried out a superposition of the ^1^H-NMR individual spectra of CUR, DMC, and BDMC in pure forms (Figure 13), and the resulting spectrum does not represent the signal patterns obtained when comparing the spectra of turmeric (natural) or C-3 mixture (synthetic), possibly caused by mutual anisotropic interactions from each curcuminoid.

The ^1^H-NMR spectrum of C-3 from natural origin is rather informative. When the spectrum is obtained in DMSO-*d6*, three singlets of methine protons are observed at ca. 6.05 ppm (Figure 13). Some additional signals of low intensity correspond to small impurities present. The signals of CUR stand out clearly, while the other minor curcuminoids correspond to DMC and BDMC. Furthermore, the close structural resemblance explains the difficulty of chromatographic or crystallographic separation when the three components (CUR, DMC, and BDMC) come from a mixture of co-crystallised powders.

To support the chromatographic and spectroscopic analyses, mass spectrometry (MS) has been obtained for individual curcuminoids and C-3 mixtures (see Supplementary Materials). Curcuminoids exhibit the expected molecular ion peaks with *m*/*z* = 368 (CUR), *m*/*z* = 338 (DMC), and *m*/*z* = 309 (BDMC) that correlate with the expected formulas C_21_H_20_O_6_, C_20_H_18_O_5_, and C_19_H_16_O_4_ respectively, while the mixtures of turmeric curcuminoids and those obtained synthetically show the three peaks (*m*/*z* = 368, *m*/*z* = 338, and *m*/*z* = 309) along the molecular ions in the same spectrum. All mass spectra present the fragmentation expected and show the ion of (4-hydroxy-3-methoxyphenyl) acrylaldehyde (*m*/*z* = 177) or (4-hydroxyphenyl) acrylaldehyde (*m*/*z* = 147), which correspond to fragmentation of the central part of the curcuminoids with molecular formulas C_10_H_10_O_3_ and C_9_H_8_O_2_**,** respectively.

In the antioxidant test, we found that CUR is the most potent phenolic antioxidant compound compared to DMC and BDMC (see Table 2), attributed to the fact that the ortho-methoxyphenol systems allow H-donation [40], capture, and stabilisation of free radicals (DPPH). Although BDMC has two phenolic groups, its antioxidant activities are lower (IC_50_ = > 100µM) compared to curcuminoids CUR and DMC, a property that correlates with the lack of methoxy groups in the ortho positions, which does not allow an effective donation of H atoms. The complete statistical analysis of the antioxidant tests is found in Tables S1–S4 and Figures S62–S64.

The analysis of curcuminoid activities (DPPH capture and TBARS inhibition) has been carried out, estimating the individual molecular contribution, in terms of the degree of the oxygenated substituents (phenol and methoxyl groups). However, other studies suggest using the term “residual complexity” (RC) [38], where impurities or hidden elements are considered to have a more complete image of biological activities from curcuminoids. From RC, the turmeric rhizome has been segmented into four important components: (a) lipophilic metabolites, (b) pristine curcumin, (c) mixture C-3, and (d) hydrophilic metabolites, and all have been associated with important biological activities.

The results of the antioxidant assay presented in Table 3 correspond to C-3 mixtures from natural and synthetic sources. Although the curcumin molecule presents the highest concentration (from turmeric (74.5%) and from experimental condition (ii) (64.1%)) in the mixtures with greater potency, it cannot be conclusively stated that the curcumin molecule is the primary substance responsible for all the antioxidant activity in the turmeric extract or the synthetic C-3 mixtures since the other two curcuminoids (DMC and BDMC) are also active.

Table 3 also shows the samples that were better than a-tocopherol for the free radical capture test (DPPH) and were inhibited with low concentrations (e.g., IC_50_ = 1.43 µg/mL) the TBARS. This study is consistent with previous reports [41,42], which attributed the presence of a pyrocatechin group to increase the stability of phenoxide radicals. The presence of two conjugated -C=C- double bonds and the aromatic rings makes an additional contribution.

Therefore, based on these studies, it is important to expand the biological tests of all curcuminoids, including those that have been little or not studied at all. In this way, overlooking active substances such as DMC and BDMC—both of which possess critical biological activities but that have not yet received as much attention and study as curcumin itself—would be avoided.

## 4. Materials and Methods

Acetic anhydride, pyridine, vanillin, 4-hydroxybenzaldehyde, n-butylamine, boron trifluoride, THF complex (CAS 462-34-0), tributyl borate, alumina Brockmann III (1344-28-1), SiO_2_, *Curcuma longa* (Turmeric) powder (Batch number: 023K3482), and all solvents (ethyl acetate, n-hexane, methanol) HPLC-grade were purchased from Sigma-Aldrich (St. Louis, MO, USA) and were used without prior purification.

Melting points were determined on an Electrothermal Engineering IA9100 (London, UK) digital melting point apparatus in open capillary tubes and were uncorrected [43].

^1^H and ^13^C NMR spectra were obtained in a Bruker Fourier 400 MHz or 500 MHz (Billerica, MA., USA) spectrometer using TMS as an internal reference and CDCl_3_ or Acetone-*d_6_*, or DMSO-*d_6_* as deuterated solvents. NMR spectra were processed with MestreNova software 12.0.0 [44] and are found in the Supplementary Materials.

IR absorption spectra were recorded using an FT-IR Bruker Tensor 27 spectrophotometer (Billerica, MA, USA) in the range of 4000–400 cm^−1^ as KBr pellets (see Supplementary Materials).

Mass spectra were recorded using the MStation JMS-700 JEOL equipment (JEOL de Mexico SA de CV, Mexico, CDMX) (Electron Ionisation, 70 eV, 250 °C, impact positive mode, and calibration standard with perfluorokerosene) and the AccuTOF JMS-T100LC JEOL (JEOL de Mexico SA de CV, Mexico, CDMX) equipment (DART^+^, 350 °C, positive ion mode and calibration standard with PEG 600). All mass spectra are shown in the Supplementary Materials.

Quantification by HPLC [45] of each curcuminoid from turmeric (Sigma-Aldrich, code C1386) and synthetic complexes C-3 was recorded using an Agilent 1200 equipment with detector of diodes-UV Waters-2996 at 425 nm, solvent isocratic from acetonitrile/water 55:45 (orthophosphoric acid 0.02%) flow rate 1 mL/min, and Spherisorb C18 5 μm, 250 mm × 4.6 mm as stationary phase.

Predicted energy changes associated with chemical reactions were estimated computationally using thermochemical calculations from vibrational frequencies, as implemented in Gaussian 16 [46]. Calculations were carried out using DFT B3LYP with aug-cc-PVDT basis. Some calculations were repeated with aug-cc-PVTZ with only minor differences in energies and overall geometries; therefore, the less computationally expensive basis was favoured for most of the calculations. The predicted differences *ΔG*°, *ΔH*°, *ΔS*°, and *ΔCv* for the chemical steps under analysis were obtained as the difference in each standard value between the products and reactants, taking into consideration stoichiometry. Equation (1) illustrates the basic principle for Gibbs free energy. Other parameters were derived in a similar fashion. Values reported are calculated considering standard states (1 atm, 298 K) and in a vacuum.

(1) $$\Delta G_{Reaction}^{0}=m_{1}G_{P_{1}}^{0}+m_{2}G_{P_{2}}^{0}+\ldots -n_{1}G_{R_{1}}^{0}-n_{2}G_{R_{2}}^{0}-\ldots$$

Equation (1). Parameter difference from thermochemical calculations. Here, $n_{1}$ and $n_{2}$ are the stoichiometric coefficients for the reactants 1 and $R_{2}$, respectively, while $m_{1}$ and $m_{2}$ are the stoichiometric coefficients for the products $P_{1}$ and $P_{2}$, respectively. $G_{X}^{0}$ represents the DFT electronic energy with the thermal correction for free energy for the X chemical species.

The free radical scavenging activity was measured using a modified method from Mellors and Tappel [47]. The test was carried out on 96-well microplates. A 50 µL aliquot of the solution of the test compound was mixed with 150 µL of an ethanol solution of DPPH (final concentration 100 µM). This mixture was incubated at 37 °C for 30 min, and the absorbance was then measured at 515 nm using a BioTek microplate reader SYNERGY HT. The inhibition per cent for each compound was determined by comparison with a 100 µM DPPH ethanol blank solution.

As an index of lipid peroxidation, TBARS levels were measured using rat brain homogenates according to the method described by Ng and co-workers [48]. The concentration of TBARS was calculated by interpolation on a standard curve of tetramethoxypropane (TMP) as a precursor of MDA [49]. Results are expressed as nmol of TBARS per mg of protein. The inhibition ratio (IR [%]) was calculated using the formula IR = (C − E) × 100/C, where C is the control absorbance and E is the sample absorbance. Quercetin and α-tocopherol were used as positive standards. All data were represented as mean ± standard error (SEM). Data were analysed by one-way analysis of variance (ANOVA), followed by Dunnett’s test for comparison against the control. Values of *p* ≤ 0.05 (∗) and *p* ≤ 0.01 (∗∗) were considered statistically significant.

### 4.1. Starting Materials

Synthesis of synthon 1.

Was synthesised as previously indicated [29] and was characterised by ^1^H RMN (see Supplementary Materials).

2,2-difluoro-4,6-dimethyl-2H-1,3,2-dioxaborinin-1-ium-2-uide (Synthon 1): yield 95%, solid amber, melting point 40 °C, ^1^H NMR (400 MHz, CDCl_3_, TMS): δ 5.96 (s, 1H, Methine-H), 2.27 (s, −6H, Methyl-H).

Synthesis of acetyl aldehydes.

In a 250 mL round flask, 70 mmol of aldehyde (vanillin or 4-hydroxybenzaldehyde) was dissolved in 150 mL of CH_2_Cl_2._ Subsequently, 70 mmol of anhydrous pyridine (Py) and 70 mmol of acetic anhydride were added at room temperature. The reaction was monitored by TLC (mobile phase Hex/EtOAc (n-hexane/ethyl acetate) 70::30). After the reaction was completed, the CH_2_Cl_2_ was removed, and the product was extracted in a mixture of EtOAc (150 mL) and H_2_O (3 × 150 mL). The organic layer was dried over anhydrous Na_2_SO_4_, and the solvent was removed in vacuum, yielding 95% (vanillin acetate) and 90% (4-acetoxybenzaldehyde). These raw materials were identified by ^1^H RMN (see Supplementary Materials).

4-Acetoxybenzaldehyde ^1^H NMR (400 MHz, CDCl_3_) δ 9.99 (s, 1H), 7.99–7.85 (m, 2H), 7.37–7.24 (m, 2H), 2.34 (s, 3H).

Vanillin acetate ^1^H NMR (400 MHz, CDCl_3_) δ 9.95 (s, 1H), 7.50 (d, *J* = 1.8 Hz, 1H), 7.48 (dd, *J* = 7.9, 1.8 Hz, 1H), 7.22 (d, *J* = 7.9 Hz, 1H), 3.91 (s, 3H), 2.34 (s, 3H).

### 4.2. Synthesis Curcuminoid-BF_2_

General methodology:

Mixture 1. In a 100 mL Erlenmeyer flask, 50 mmol of acetyl aldehyde (vanillin acetate or 4-acetoxybenzaldehyde) was dissolved in 50 mL of EtOAc (ethyl acetate), then 6.8 mL of tributyl borate (25 mmol) was added, and this mixture was heated until homogenised.

Mixture 2. In a 250 mL round flask, 4.07 g of synthon 1 (1.1 eq, 27 mmol) was dissolved in 25 mL of EtOAc, and the homogenised mixture 1 was added to the solution. Then, 2.7 mL of N-butylamine (27 mmol, in 25 mL of EtOAc) drop by drop was added. The mixture of reaction was left overnight in magnetic stirring at room temperature. Finally, an orange colour solid was filtered and washed with a mixture of 50 mL water/acetone 90::10, yield: 95% (compound 1) and 86% (compound 2).

((1*E*,1’*E*)-(2,2-difluoro-2*H*-1λ3,3,2λ4-dioxaborinine-4,6-diyl)bis(ethene-2,1-diyl))bis(2-methoxy-4,1-phenylene) diacetate, Compound 1, orange powder, melting point = 240 °C. ^1^H NMR (400 MHz, CDCl_3_) δ 7.94 (d, *J* = 15.6 Hz, 2H), 7.18 (dd, *J* = 8.2, 1.9 Hz, 2H), 7.11 (d, *J* = 2.0 Hz, 2H), 7.06 (d, *J* = 8.1 Hz, 2H), 6.63 (d, *J* = 15.5 Hz, 2H), 6.08 (s, 1H), 3.85 (s, 6H), 2.31 (s, 6H). ^13^C NMR (100 MHz, CDCl_3_) δ 180.24, 168.81, 151.87, 146.99, 143.03, 133.08, 123.86, 122.49, 120.90, 112.58, 102.54, 56.20, 20.87. IR (KBr) 1763 v(C=O), 1615 v(C=O), 1541 v(C=C), 1503 v(C=O, C=C), 1152 v(B-F, B-O). EI^+^-MS: *m*/*z* = 500, *m*/*z* calc. = 500.

((1*E*,1’*E*)-(2,2-difluoro-2*H*-1λ3,3,2λ4-dioxaborinine-4,6-diyl)bis(ethene-2,1-diyl))bis(4,1-phenylene) diacetate, Compound 2, orange powder, melting point = 242 °C. ^1^H NMR (400 MHz, CDCl_3_) δ 7.96 (d, *J* = 15.6 Hz, 2H), 7.60–7.55 (m, 4H), 7.14–7.08 (m, 4H), 6.64 (d, *J* = 15.6 Hz, 2H), 6.07 (s, 1H), 2.24 (s, 6H). ^13^C NMR (100 MHz, CDCl_3_) δ 180.28, 169.03, 153.50, 146.46, 131.76, 130.52, 122.66, 120.78, 102.46, 21.24. IR (KBr) 1757 v(C=O), 1615 v(C=O), 1529 v(C=C), 1158 v(B-F, B-O). DART^+^-MS: *m*/*z* = 441, *m*/*z* calc. = 440.

### 4.3. Reaction Conditions for Cleavage of BF_2_ Group and Obtention of Symmetric Curcuminoids (CUR and BDMC)

A methodology like the one reported previously was used and is described here. In a 500 mL round flask, 10 g of Curcuminoids-BF_2_ symmetric (compound 1 or 2) was dissolved in 400 mL of methanol (MeOH), 20% weight of Al_2_O_3_ (catalyst) and 10 mL of HCl were added to the solution. The reaction was left for 5 h under magnetic stirring at reflux. The reaction was quenched by filtration using a sintered glass funnel packed with celite. MeOH was evaporated in vacuum, and the reaction crude was extracted with 200 mL of EtOAc (Ethyl acetate) and water (3 × 100 mL). The organic phase was dried under Na_2_SO_4_ and concentrated in a vacuum to afford the curcuminoid product, which was purified by flash chromatography using Hexane-EtOAc 70:30, With this methodology, curcumin and bis-demethoxycurcumin were obtained with yields of 55% (curcumin, compound 3) and 70% (bis-demethoxycurcumin, compound 4).

1,7-bis-(4-hydroxy-3-methoxy-phenyl)-1,6-heptadien-3,5-dione, curcumin, compound 3, yellow-orange powder, melting point = 185 °C. ^1^H NMR (400 MHz, DMSO-*d*6): δ 16.47 (br, 1H, Enol-H), 9.66 (br, 2H, Phenol-OH), 7.55 (d, *J* = 15.8 Hz, 2H, Vinyl-H), 7.32 (d, *J* = 1.89 Hz, 2H, Aryl-H), 7.15 (dd, *J* = 8.2; 1.93 Hz, 2H, Aryl-H), 6.82 (d, *J* = 8.13 Hz, 2H, Aryl-H), 6.75 (d, *J* = 15.81 Hz, 2H, Vinyl-H), 6.06 (s, 1H, Methine-H), 3.84 (s, 6H, Methoxy-OCH_3_); ^13^C NMR (100 MHz, DMSO-*d*6): δ 183.22 (C=O), 149.36 (C-OH), 148.00 (Caryl), 140.72 (Cvinyl-H), 126.34 (Caryl), 123.14 (Caryl-H), 121.10 (Cvinyl-H), 115.70 (Caryl-H), 111.33 (Caryl-H), 100.85 (C_methine_-H), 55.69 (-OCH3). IR (KBr) 3506 ν(OH), 1628 ν(C=O), 1602 ν(C=C ring), 1509 ν(C=O, C=C), 1428 ν(C-O phenol), 1281 ν(C-O enol), 1154 ν(C-O), 1028 ν(=C-O-CH_3_) cm−1, EI-MS: *m*/*z* = 368, *m*/*z* calc. = 368.

1,7-bis(4-hydroxyphenyl)-1,6-heptadien-3,5-dione, bis-demethoxycurcumin, compound 4, red-orange powder, melting point = 215 °C. ^1^H NMR (400 MHz, DMSO-*d6*): δ 10.04 (br, 2H,-OH), 7.55 (d, J = 15.8 Hz, 2H, Vinyl-H), 7.55 (m, 4H, Aryl-H), 6.82 (m, 4H, Aryl-H), 6.69 (d, J = 15.8 Hz, 2H, Vinyl-H), 6.04 (s, 1H, Methine-H); ^13^C NMR (100 MHz, DMSO-*d6*): δ 183.20 (C=O), 159.79 (C-OH), 140.35 (Cvinyl-H), 130.31 (Caryl-H), 125.82 (Caryl), 120.78 (Cvinyl-H), 115.90 (C aryl-H), 100.93 (C methine-H). IR (KBr) 3232 ν(OH), 1622 ν(C=O), 1599 ν(C=O), 1513 ν(C=O, C=C), 1444 ν(OH), 1276 ν(C-O enol), 1140 ν(C-O) cm^−1^, DART^+^-MS: *m*/*z* = 308, *m*/*z* calc. = 308.

### 4.4. Separation of Demethoxycurcumin (DMC)

A total of 200 mg of the C-3 curcuminoid mixture enriched with demethoxycurcumin was dissolved in 1 mL of acetone and applied to a 20 × 20 cm preparative plate, then eluted with a ternary mixture of solvents (CH_2_Cl_2_: Hex: MeOH: 65:30:5) three times, the fraction intermediate was extracted with ethyl acetate, the solvent was evaporated in a vacuum, and a 50% yield was obtained.

(1*E*,4*Z*,6*E*)-5-hydroxy-1-(4-hydroxy-3-methoxyphenyl)-7-(4-hydroxyphenyl)hepta-1,4,6-trien-3-one, demethoxycurcumin, compound 5, orange powder, melting point = 165 °C. ^1^H NMR (500 MHz, DMSO-*d*_6_): δ 16.44 (s, 1H, Enol-H), 10.08 (s, 1H, Phenol-OH), 9.68 (s, 1H, Phenol-OH), 7.55 (m, 4H, Aryl-H and Vinyl-H), 7.32 (d, *J* = 1.97 Hz, 1H, Aryl-H), 7.14 (dd, *J* = 8.24, 1.98 Hz, 1H, Aryl-H), 6.82 (m, 3H, Aryl-H), 6.76 (d, *J* = 15.78 Hz, 1H, Vinyl-H), 6.69 (d, *J* = 15.85 Hz, 1H, Vinyl-H), 6.04 (s, 1H, Methine-H), 3.83 (s, 3H,Methoxy-OCH_3_). ^13^C NMR (125 MHz, 500 MHz, DMSO-*d*_6_): δ 183.31 (C=O), 183.18 (C=O), 159.84 (C-OH), 149.37 (C-OH), 148.02 (Caryl), 140.75 (Cvinyl-H), 140.41 (Cvinyl-H), 130.39 (C aryl-H), 126.36 (Caryl), 125.85 (Caryl), 123.26 (C aryl-H), 121.06 (Cvinyl-H), 120.85 (Cvinyl-H), 115.94 (C aryl-H), 115.69 (C aryl-H), 111.21(C aryl-H), 100.98 (Cmethine-H), 55.70 (-OCH_3_). IR (KBr) 3397 v(OH), 1624 v(C=O), 1574 v(C=O, 1510 ν(C=O, C=C), 1437 ν(C-O phenol), 1133 ν(C-O). EI-MS: *m*/*z* = 338, *m*/*z* calc. = 338.

### 4.5. Synthesis Curcuminoid-BF_2_ Complexes C-3 Precursors

General methodology.

The scrambling of aldehydes for experimental conditions (i) were as follows: in a 100 mL Erlenmeyer flask, 25 mmol of vanillin and 25 mmol of 4-hydroxybenzaldehyde were dissolved in 50 mL of EtOAc (ethyl acetate), then 6.8 mL of tributyl borate (25 mmol) was added, and this mixture was heated until homogeneity was reached (about 10 min).

Condensation reaction. In a 250 mL round flask, 3.7 g of synthon 1 (25 mmol) was dissolved in 50 mL of EtOAc, then the homogenate from scrambling (i) was added dropwise to the solution. Next, 2.7 mL of N-butylamine (27 mmol, in 25 mL of EtOAc) was added drop by drop, the mixture of reaction was left for 12 h in magnetic stirring at room temperature. Finally, the reaction mixture was washed with 50 mL of water (5% of HCl), the organic phase was extracted and dried under anhydrous Na_2_SO_4_, and the solvent was removed in vacuum. A violet solid was isolated with an overall yield of 90% and a melting point of 115 °C. ^1^H NMR (400 MHz, Acetone-*d_6_*): δ 8.62 (s, 3H), 7.98–7.90 (m, 12H), 7.46 (d, J = 2.0 Hz, 3H), 7.35 (dd, J = 8.3, 2.0 Hz, 3H), 6.97–6.93 (m, 9H), 6.93–6.89 (m, 6H), 6.35 (s, 3H), 3.94 (s, 9H). ^13^C NMR (100 MHz, Acetone-*d_6_*): δ 180.63, 151.95, 151.82, 149.04, 149.00, 147.68, 127.70, 125.83, 119.15, 116.62, 116.55, 112.64, 102.07, 56.43.

The scrambling of aldehydes for experimental condition (ii) was as follows: in a 100 mL Erlenmeyer flask, 39 mmol of vanillin and 11 mmol of 4-hydroxybenzaldehyde were dissolved in 50 mL of EtOAc (ethyl acetate), then 6.8 mL of tributyl borate (25 mmol) was added, and this mixture was heated until homogenised for 10 min.

Condensation reaction. In a 250 mL round flask, 3.7 g of synthon 1 (25 mmol) was dissolved in 50 mL of EtOAc. Then, the homogenised aldehydes from scrambling (ii) were added dropwise to the solution, and later, 2.7 mL of n-butylamine (27 mmol in 25 mL of EtOAc) was added drop by drop. The mixture of the reaction was left for 12 h in magnetic stirring at room temperature. Finally, the reaction mixture was washed with 50 mL of water (5% of HCl), and the organic phase was extracted and dried under anhydrous Na_2_SO_4_, and the solvent was removed in a vacuum. A red solid was isolated with an overall yield of 92% and a melting point of 232 °C. ^1^H NMR (400 MHz, Acetone-*d_6_*): δ 8.62 (s, 3H), 7.98–7.90 (m, 12H), 7.46 (d, J = 2.0 Hz, 3H), 7.35 (dd, J = 8.3, 2.0 Hz, 3H), 6.97–6.93 (m, 9H), 6.93–6.89 (m, 6H), 6.35 (s, 3H), 3.94 (s, 9H). ^13^C NMR (101 MHz, Acetone-*d_6_*): δ 180.63, 151.95, 151.82, 149.04, 149.00, 147.68, 127.70, 125.83, 119.15, 116.62, 116.55, 112.64, 102.07, 56.43.

Scrambling of aldehydes for experimental condition (iii). In a 100 mL Erlenmeyer flask, 25 mmol of vanillin acetate and 25 mmol of 4-acetoxybenzaldehyde was dissolved in 50 mL of EtOAc (ethyl acetate); then, 6.8 mL of tributyl borate (25 mmol) was added and this mixture was heated until homogenised for 10 min.

Condensation reaction: in a 250 mL round flask 3.7 g of synthon 1 (25 mmol) was dissolved in 50 mL of EtOAc, then homogenised from scrambling (iii), and added dropwise to the solution. Next, 2.7 mL of N-butylamine (27 mmol, in 25 mL of EtOAc) was added drop by drop. The mixture of reaction was left for 12 h in magnetic stirring at room temperature. Finally, the reaction mixture was filtered off and washed with 10 mL a mixture of acetone/water 1:1. A yellow-orange solid was obtained with overall yield of 95% and melting point of 215 °C. ^1^H NMR (400 MHz, Acetone-*d_6_*): δ 8.09–8.01 (m, 6H), 7.92–7.87 (m, 6H), 7.63–7.59 (m, 3H), 7.48–7.43 (m, 3H), 7.30–7.24 (m, 6H), 7.21–7.15 (m, 3H), 7.15–7.08 (m, 6H), 6.55 (s, 1H), 6.53 (s, 1H), 6.52 (s, 1H), 3.92 (s, 9H), 2.29 (s, 9H), 2.27 (s, 9H). ^13^C NMR (100 MHz, Acetone-*d_6_*): δ 181.64, 181.60, 169.48, 168.86, 154.72, 152.96, 147.10, 146.66, 146.63, 144.01, 134.21, 132.89, 131.56, 124.59, 123.67, 123.52, 123.45, 122.46, 122.34, 113.80, 113.75, 103.16, 103.09, 103.01, 56.55, 21.08, 20.57.

### 4.6. Reaction Conditions for Cleavage of BF_2_ Group and Obtention of C-3 Complexes (CUR, DMC and BDMC)

A methodology like the one reported previously was used [29] and is described here. In a 500 mL round flask, 5 g of Curcuminoids-BF_2_ precursors (from experimental conditions (i), (ii), and (iii)) was dissolved in 400 mL of methanol (MeOH), 20% weight of Al_2_O_3_ (catalyst) and 5 mL of HCl was added to the solution. The mixture of reaction was left for 5 h under magnetic stirring at reflux. The reaction was quenched by filtration using a sintered glass funnel packed with celite. MeOH was evaporated in a vacuum, and the reaction crude was extracted with 200 mL of EtOAc (ethyl acetate) and water (3 × 100 mL). The organic phase was dried under Na_2_SO_4_ and concentrated in a vacuum to afford C-3 mixture products, which were purified by flash chromatography using Hexane-EtOAc 1:1 (this mixture of solvents does not afford resolution of the individual curcuminoids from C-3 complexes). The mixtures in enol form (C-3 curcuminoids) were obtained in 55% (experimental condition (i)), 70% (experimental condition (ii)), and 74% (experimental condition (iii)). All products were characterised by spectroscopic methods (see Supplementary Materials), though NMR spectra were not assigned due to the heavy overlap of signals.

C-3 complex from experimental condition (i), orange powder, melting point = 158 °C. ^1^H NMR (400 MHz, Acetone-*d_6_*) δ 16.43 (s, 3H), 8.95 (s, 3H), 8.21 (s, 3H), 7.64–7.54 (m, 12H), 7.34 (dd, J = 3.3, 2.0 Hz, 3H), 7.18 (dd, J = 8.3, 2.0 Hz, 3H), 6.90 (t, J = 8.2 Hz, 9H), 6.73 (m, 2H), 6.69 (m, 2H), 6.65 (m, 2H), 5.97 (s, 3H), 3.92 (s, 9H). ^13^C NMR (100 MHz, Acetone-*d_6_*) δ 184.59, 160.59, 150.11, 148.86, 141.49, 141.13, 131.06, 128.24, 127.78, 123.97, 123.91, 122.36, 122.13, 116.87, 116.31, 111.60, 111.54, 101.81, 101.75, 56.37. For mass spectrometry (DART^+^) the expected molecular ions at *m*/*z* = 308 + 1, *m*/*z* = 338 + 1 and *m*/*z* = 368 + 1 were identified.

C-3 complex from experimental condition (ii), orange powder, melting point = 175 °C. ^1^H NMR (400 MHz, Acetone-*d_6_*) δ 16.43 (s, 3H), 8.96 (s, 3H), 8.22 (s, 3H), 7.65–7.54 (m, 12H), 7.33 (d, J = 2.1 Hz, 3H), 7.18 (dd, J = 8.2, 2.0 Hz, 3H), 6.93–6.85 (m, 9H), 6.75–6.63 (m, 6H), 5.97 (s, 3H), 3.92 (s, 9H). ^13^C NMR (100 MHz, Acetone-*d_6_*) δ 184.60, 160.58, 150.11, 148.86, 141.49, 141.13, 131.06, 128.24, 127.78, 123.97, 123.92, 122.37, 122.13, 116.86, 116.31, 111.60, 111.54, 101.80, 101.75, 56.37. For mass spectrometry (EI^+^) the expected molecular ions at *m*/*z* = 308, *m*/*z* = 338 and *m*/*z* = 368 were identified.

C-3 complex from experimental condition (iii), red-orange powder, melting point = 151 °C. ^1^H NMR (400 MHz, DMSO-*d_6_*) δ 16.41 (s, 3H), 9.84 (s, 3H), 7.61–7.50 (m, 12H), 7.35–7.29 (m, 3H), 7.18–7.12 (m, 3H), 6.85–6.80 (m, 9H), 6.79–6.73 (m, 4H), 6.69 (d, J = 15.9 Hz, 2H), 6.06 (s, 1H), 6.04 (s, 1H), 6.04 (s, 1H), 3.84 (s, 9H). ^13^C NMR (100 MHz, DMSO- *d_6_*) δ 183.27, 183.21, 183.15, 159.81, 149.36, 148.00, 140.72, 140.38, 130.35, 126.34, 125.83, 123.20, 123.14, 121.09, 121.04, 120.82, 120.79, 115.92, 115.70, 111.33, 111.24, 100.94, 100.86, 55.69. For mass spectrometry (DART^+^) the expected molecular ions at *m*/*z* = 308 +1, *m*/*z* = 338 +1 and *m*/*z* = 368 +1 were identified.

## 5. Conclusions

We have successfully applied the aldehyde scrambling method followed by aldolic condensation for the synthesis of the main C-3 curcuminoids isolated from turmeric. The synthesis of the single unsymmetric curcuminoid demethoxycurcumin cannot be obtained following this approach. However, it can be isolated by chromatographic means starting from an enriched mixture obtained using the scrambling approach.

It should be noted that an interesting contribution of this methodology is that the proportion found in the natural sources of the three main curcuminoids in the relative proportion CUR > DMC > BDMC can be closely mimicked by a synthetic pathway. An additional advantage that stands out is that agronomic restrictions and the usual content of the components that are present in the natural source, such as fibers, essential oils, and other secondary metabolites, are avoided. The present synthetic approach also requires smaller amounts of solvents compared to the extractive process used with plant material, which lends it a green chemistry approach.

The nearly unlimited availability of the C-3 complex of curcuminoids is likely to enhance the performance of numerous acute and chronic biological assays in vivo. This expansion could further stimulate research into its anti-inflammatory and antioxidant effects, significantly advancing its development as a nutraceutical and a potential preventive measure for diverse chronic human ailments.

## 6. Patents

An application for a patent is underway in the country of the authors.

## Figures and Tables

**Figure 5:**
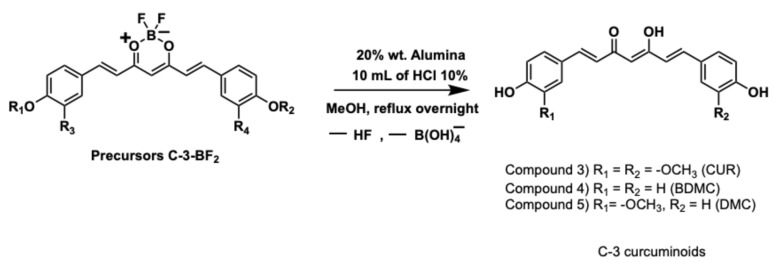
Removal of BF_2_ group and synthesis of C-3 curcuminoids.

**Table 1:** Percentage of curcuminoids by HPLC.

Mixture C-3	CUR %	DMC %	BDMC %
C-3 from turmeric	74.52	17.36	8.12
C-3-Condition i	42.79	49.09	8.12
C-3 Condition ii	64.10	33.49	2.32
C-3 Condition iii	31.21	51.59	17.20

**Table 2:** Capture of DPPH radical and TBAR inhibition by curcuminoids.

Curcuminoid	DPPH IC_50_ (µM)	TBARS IC_50_ (µM)
CUR	29.97 ± 1.73	1.12 ± 0.08
DMC	46.04 ± 1.70	1.51 ± 0.19
BDMC	>100	3.71 ± 0.14
α-tocopherol	41.15 ± 0.14	-
Quercetin	10.87 ± 0.40	-

**Table 3:** Capture of radical DPPH and TBARS inhibition by C-3 mixtures.

Mixture C-3	DPPH IC_50_ (µg/mL)	TBARS IC_50_ (µg/mL)
Curcuminoids from turmeric	8.75 ± 0.17	1.24 ± 0.04
C-3 Condition (i)	13.19 ± 0.13	1.87 ± 0.11
C-3 Condition (ii)	9.21 ± 0.05	1.43 ± 0.10
C-3 Condition (iii)	13.17 ± 0.24	1.50 ± 0.02
α-tocopherol	17.72 ± 0.06	-
Quercetin	3.67 ± 0.13	-

## Data Availability

The data are contained within the article and Supplementary Materials. Further inquiries can be directed to the corresponding authors.

## Supplementary Materials

The supporting information can be downloaded at: https://www.mdpi.com/article/10.3390/ijms26199599/s1.
