# Supplementary material for: One Pot Synthesis of the C-3 Complex (Curcumin, Demethoxycurcumin, and Bis-Demethoxycurcumin): Their Joint and Independent Biological Actions
Source: Int J Mol Sci. 2025 Oct 1;26(19):9599. doi: 10.3390/ijms26199599 (PMC12525456; doi:10.3390/ijms26199599)
Supplement: Supplementary file 1 [file ijms-26-09599-s001.zip › ijms-3861943-supplementary.pdf]

One Pot Synthesis of the C-3 complex (curcumin, demethoxycurcumin and bis-demethoxycurcumin): their joint and independent biological actions.

Marco A. Obregón-Mendoza [1], Rubén Sánchez-Obregón [1], Rosario Tavera-Hernández[1], Leidys L. Pérez-González[1], Antonio Nieto-Camacho[1], Rogelio Rodríguez-Sotres[2], and Raúl G. Enríquez [1,*]

[1] Instituto de Química, Universidad Nacional Autónoma de México, Circuito Exterior, Ciudad Universitaria, Ciudad de México 04510, Mexico.

[2] Facultad de Medicina, Universidad Nacional Autónoma de México, Mexico City CDMX 04510, Mexico.

* Correspondence: enriquezhabib@gmail.com; Tel.: 55 5622 4404

Figure S1. ^{1}H-NMR spectrum of synthon 1, $CDCl_3$, 400MHz. 5
Figure S2. ^{1}H-NMR spectrum of 4-acetoxybenzaldehyde, $CDCl_3$, 400 MHz. 6
Figure S3. ^{1}H-NMR spectrum of vanillin acetate, $CDCl_3$, 400MHz. 7
Figure S4. ^{1}H-NMR spectrum of compound 1, $CDCl_3$, 400MHz. 8
Figure S5. ^{1}H-NMR spectrum of compound 1 (aromatic region), CDCl3, 400MHz. 9
Figure S6. ^{13}C-NMR spectrum of compound 1, $CDCl_3$, 100MHz. 10
Figure S7. HSQC spectrum of compound 1, $CDCl_3$, 400MHz. 11
Figure S8. HMBC spectrum of compound 1, $CDCl_3$, 400MHz. 12
Figure S9. Mass spectrum (IE^+) of compound 1. 13
Figure S10. IR spectrum of compound 1. 14
Figure S11. ^{1}H-NMR spectrum of compound 2, $CDCl_3$ + Acetone-d6, 400MHz. 15
Figure S12. ^{13}C-NMR spectrum of compound 2, $CDCl_3$ + Acetone-d6, 100MHz. 16
Figure S13. HSQC spectrum of compound 2, $CDCl_3$ + Acetone-d6, 400MHz. 17
Figure S14. HMBC spectrum of compound 2, $CDCl_3$ + Acetone-d6, 400MHz. 18
Figure S15. Mass spectrum (IE+) of compound 2. 19
Figure S16. IR spectrum of compound 2. 20
Figure S17. ^{1}H-NMR spectrum of compound 3 (Curcumin, CUR), DMSO-d6, 400MHz. 21
Figure S18. ^{1}H-NMR spectrum (aromatic region) of compound 3, DMSO-d6, 400MHz. 22
Figure S19. ^{13}C-NMR spectrum of compound 3, DMSO-d6, 100MHz. 23
Figure S20. HSQC spectrum of compound 3, DMSO-d6, 400MHz. 24
Figure S21. HMBC spectrum of compound 3, DMSO-d6, 400MHz. 25
Figure S22. Mass spectrum (EI+) of compound 3. 26
Figure S23. IR spectrum of compound 3. 27
Figure S24. ^{1}H-NMR spectrum of compound 4 (Bis-demethoxycurcumin, BDMC), DMSO-*d6*, 400MHz. 28
Figure S25. ^{1}H-NMR spectrum (aromatic region) of compound 4, DMSO-*d6*, 400MHz. 29
Figure S26. ^{13}C-NMR spectrum of compound 4, DMSO-*d6*, 100 MHz. 30
Figure S27. HSQC spectrum of compound 4, DMSO-*d6*, 400MHz. 31
Figure S28. HMBC spectrum of compound 4, DMSO-*d6*, 400MHz. 32
Figure S29. Mass spectrum (DART+) of compound 4. 33
Figure S30. IR spectrum of compound 4. 34
Figure S31. ^{1}H-NMR spectrum of compound 5 (Demethoxycurcumin, DMC), DMSO-d6, 400MHz. 35
Figure S32. ^{1}H-NMR spectrum (aromatic region) of compound 5, DMSO-*d6*, 400MHz. 36
Figure S33. ^{13}C-NMR spectrum of compound 5, DMSO-d6, 100 MHz. 37
Figure S34. HSQC spectrum of compound 5, DMSO-d6, 400MHz 38

Figure S35. HMBC spectrum of compound 5, DMSO-d6, 400MHz. 39
Figure S36. Mass spectrum (EI^+) of compound 5. 40
Figure S37. IR spectrum of compound 5. 41
Figure S38. ^{1}H-NMR spectrum of precursors C-3-BF_2 experimental condition i), Acetone-*d6*, 400MHz. 42
Figure S39. ^{13}C-NMR spectrum of precursors C-3-BF_2 experimental condition i), Acetone-*d6*, 100MHz. 43
Figure S40. IR spectrum of precursors C-3-BF2 experimental condition i). 44
Figure S41. Mass spectrum ($DART^+$) of precursors C-3-BF_2 experimental condition i). 45
Figure S42. ^{1}H-NMR spectrum of precursors C-3-BF_2 experimental condition ii), Acetone-d6, 400MHz. 46
Figure S43. ^{13}C-NMR spectrum of precursors C-3-BF_2 experimental condition ii), Acetone-d6, 100MHz. 47
Figure S44. Mass spectrum ($DART^+$) of precursors C-3-BF_2 experimental condition ii). 48
Figure S45. IR spectrum of precursors C-3-BF_2 experimental condition ii). 49
Figure S46. ^{1}H-NMR spectrum of precursors C-3-BF_2 experimental condition iii), Acetone-*d6*, 400MHz. 50
Figure S47. ^{13}C-NMR spectrum of precursors C-3-BF_2 experimental condition iii), Acetone-*d6*, 100MHz. 51
Figure S48. Mass spectrum (DART+) of precursors C-3-BF_2 experimental condition iii). 52
Figure S49. IR spectrum of precursors C-3-BF_2 experimental condition iii). 53
Figure S50. ^{1}H-NMR spectrum of curcuminoids C-3 (CUR, DMC, BDMC) from experimental condition i), Acetone-d6, 400MHz. 54
Figure S51. ^{13}C-NMR spectrum of curcuminoids C-3 (CUR, DMC, BDMC) from experimental condition i), Acetone-d6, 100MHz. 55
Figure S52. Mass spectrum of curcuminoids C-3 (DART+) from experimental condition i). 56
Figure S53. IR spectrum of curcuminoids C-3 from experimental condition i). 57
Figure S54. ^{1}H-NMR spectrum of curcuminoids C-3 (CUR, DMC, BDMC) from experimental condition ii), Acetone-d6, 400MHz. 58
Figure S55. ^{13}C-NMR spectrum of curcuminoids C-3 (CUR, DMC, BDMC) from experimental condition ii), Acetone-d6, 100MHz. 59
Figure S56. Mass spectrum of curcuminoids C-3 (EI^+) from experimental condition ii). 60
Figure S57. IR spectrum of curcuminoids C-3 from experimental condition ii). 61
Figure S58. ^{1}H-NMR spectrum of curcuminoids C-3 (CUR, DMC, BDMC) from experimental condition iii), Acetone-d6, 400MHz. 62
Figure S59. ^{13}C-NMR spectrum of curcuminoids C-3 (CUR, DMC, BDMC) from experimental condition iii), Acetone-d6, 100MHz. 63
Figure S60. Mass spectrum (DART+) of curcuminoids C-3 from experimental condition iii). 64
Figure S61. IR spectrum of curcuminoids C-3 from experimental condition iii). 65
Table S1. DPPH Inhibition (%) of pure compounds CUR, DMC and BDMC. 66
Figure S62. Plot dose-response DPPH Inhibition (%) of pure curcuminoids (CUR, DMC and BDMC). 68
Table S2. TBARS Inhibition (%) of pure compounds CUR, DMC and BDMC. 69
Figure S63. Plot dose-response TBARS Inhibition (%) of pure curcuminoids (CUR, DMC and BDMC). 70

Table S3. DPPH Inhibition (%) of C-3 mixtures. 71
Figure S64. Plot dose-response DPPH Inhibition (%) of C-3 mixtures. 74

Table S4. TBARS Inhibition (%) of C-3 mixtures. 75
Figure S65. Plot dose-response TBARS Inhibition (%) of C-3 mixtures. 77

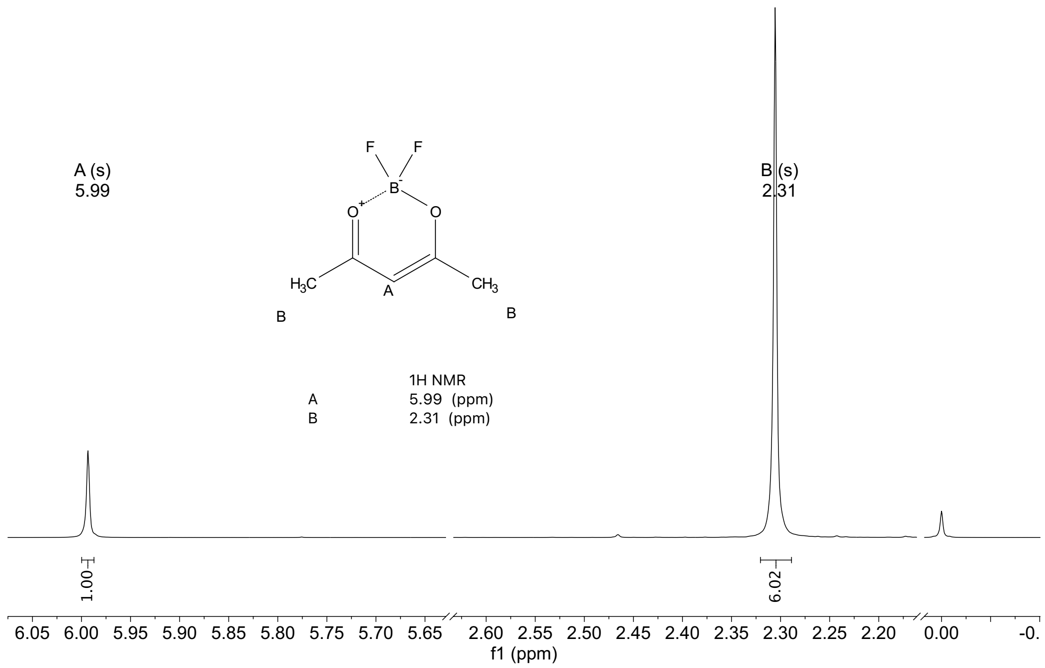

Figure S1. ^{1}H-NMR spectrum of synthon 1, $CDCl_3$, 400MHz.

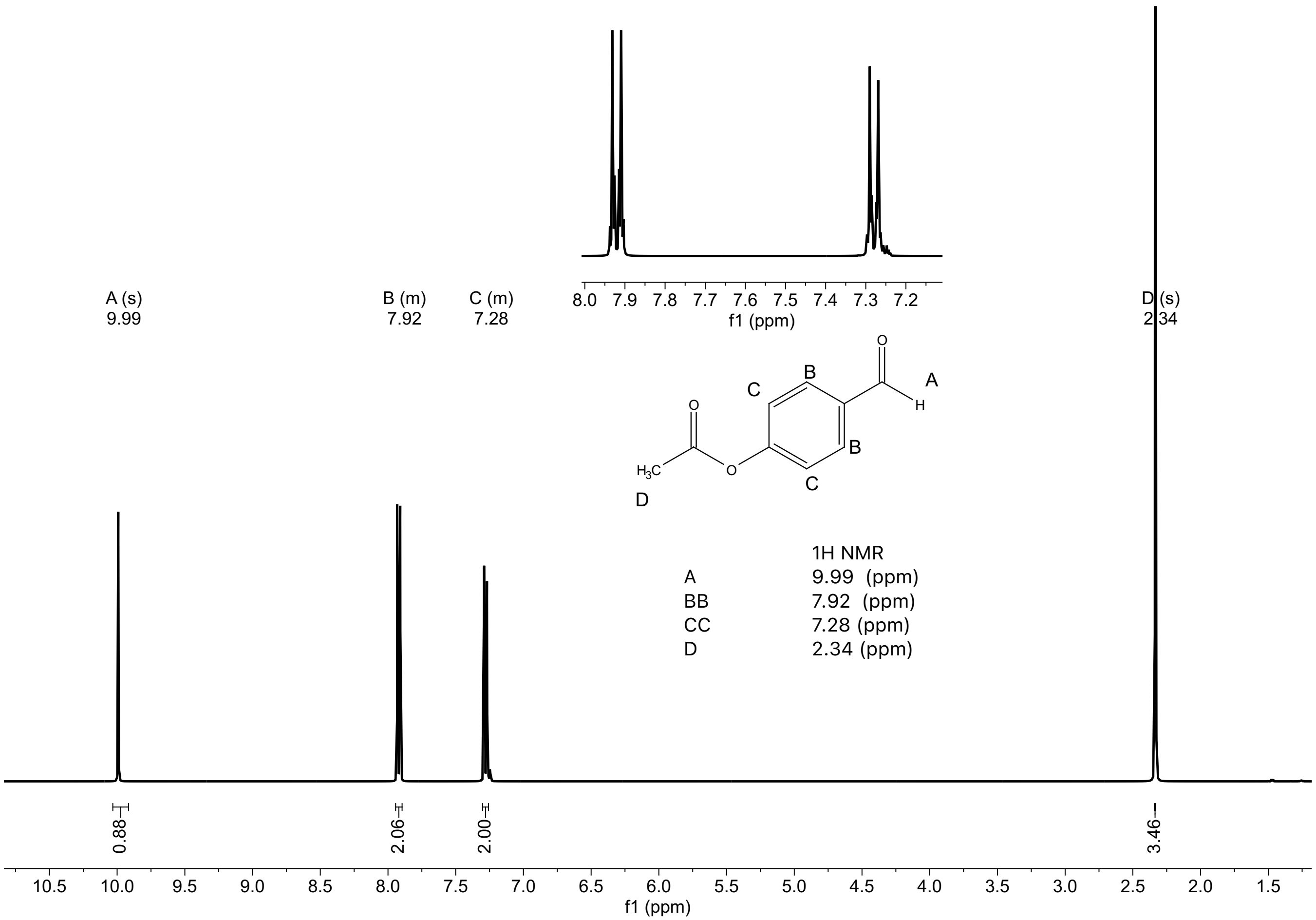

Figure S2. **^{1}H-NMR spectrum of 4-acetoxybenzaldehyde, $CDCl_3$, 400 MHz.**

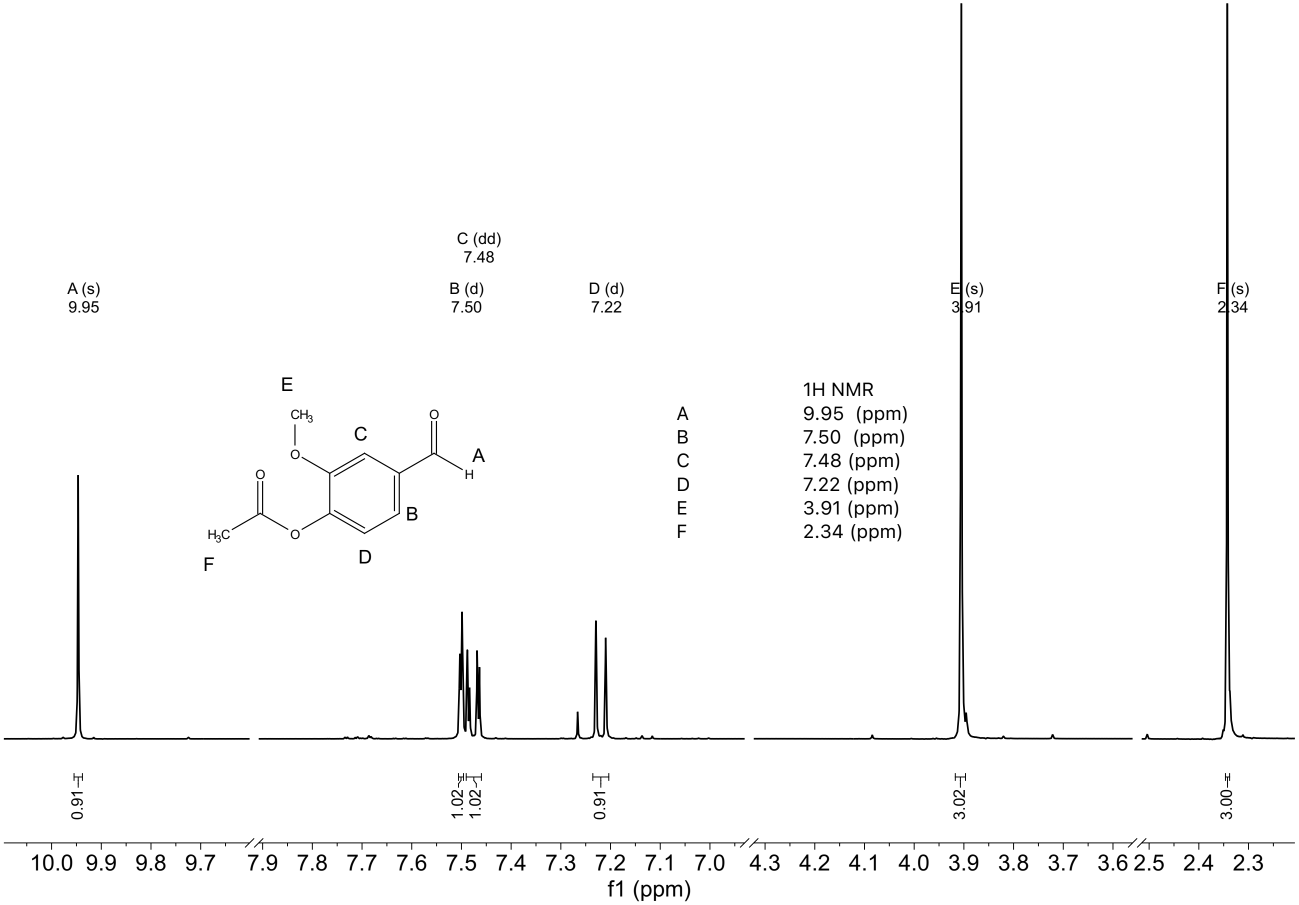

Figure S3. ^{1}H-NMR spectrum of vanillin acetate, $CDCl_3$, 400MHz.

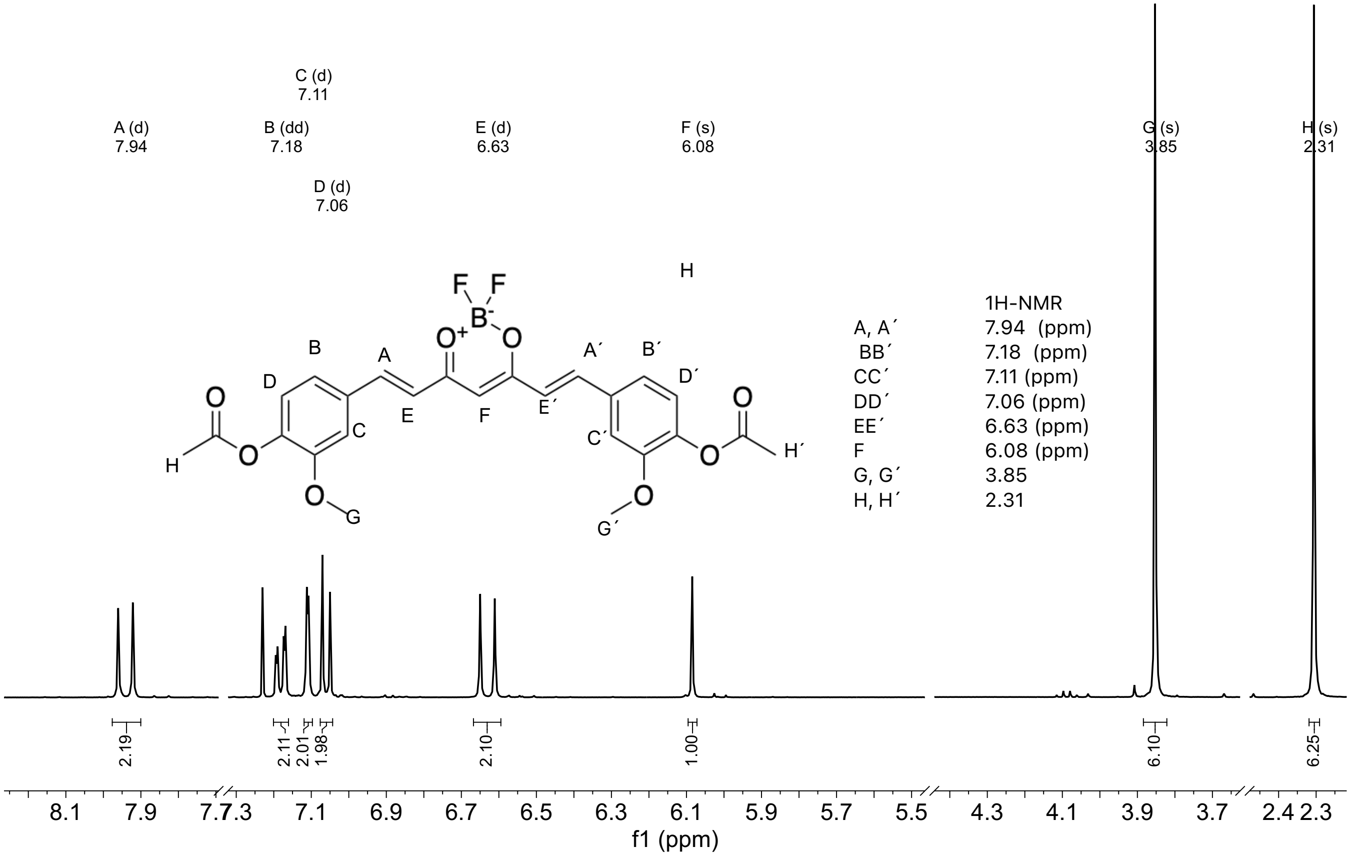

Figure S4. ^{1}H-NMR spectrum of compound 1, $CDCl_3$, 400MHz.

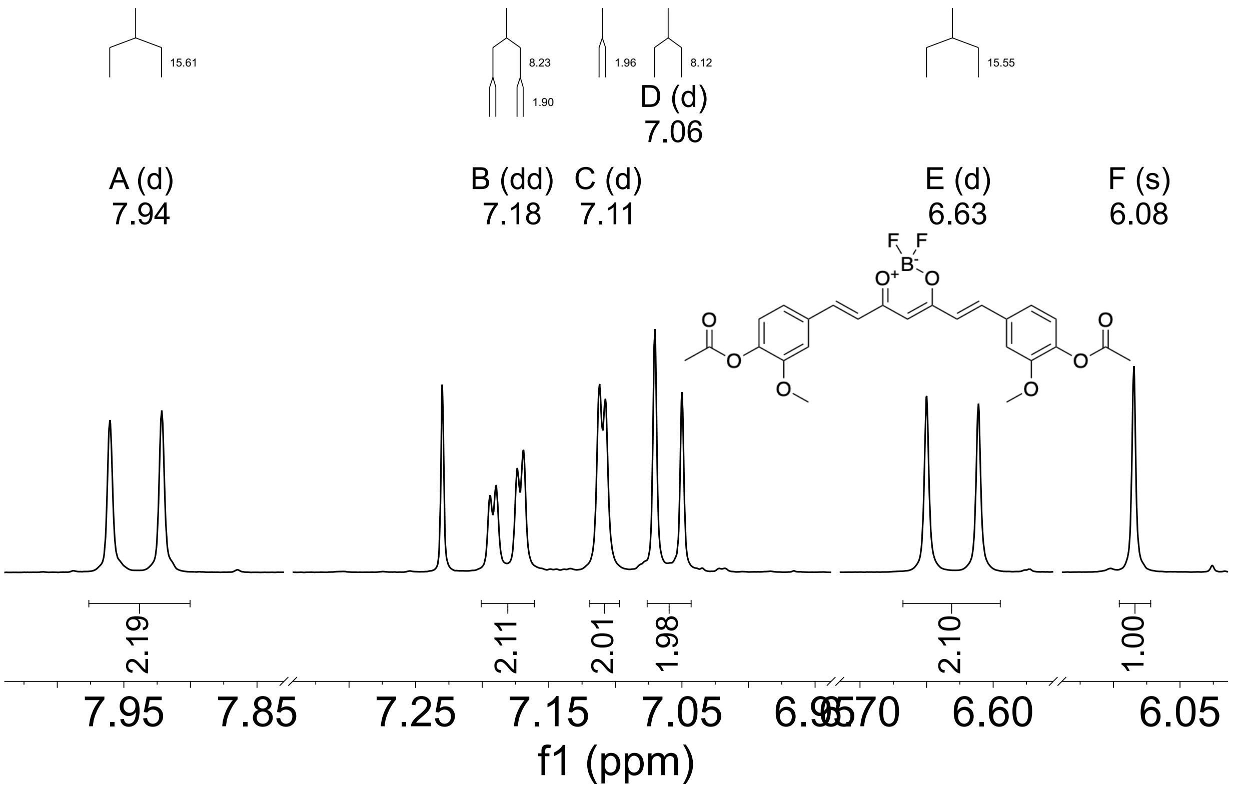

Figure S5. ¹H-NMR spectrum of compound 1 (aromatic region), $CDCl_3$, 400MHz.

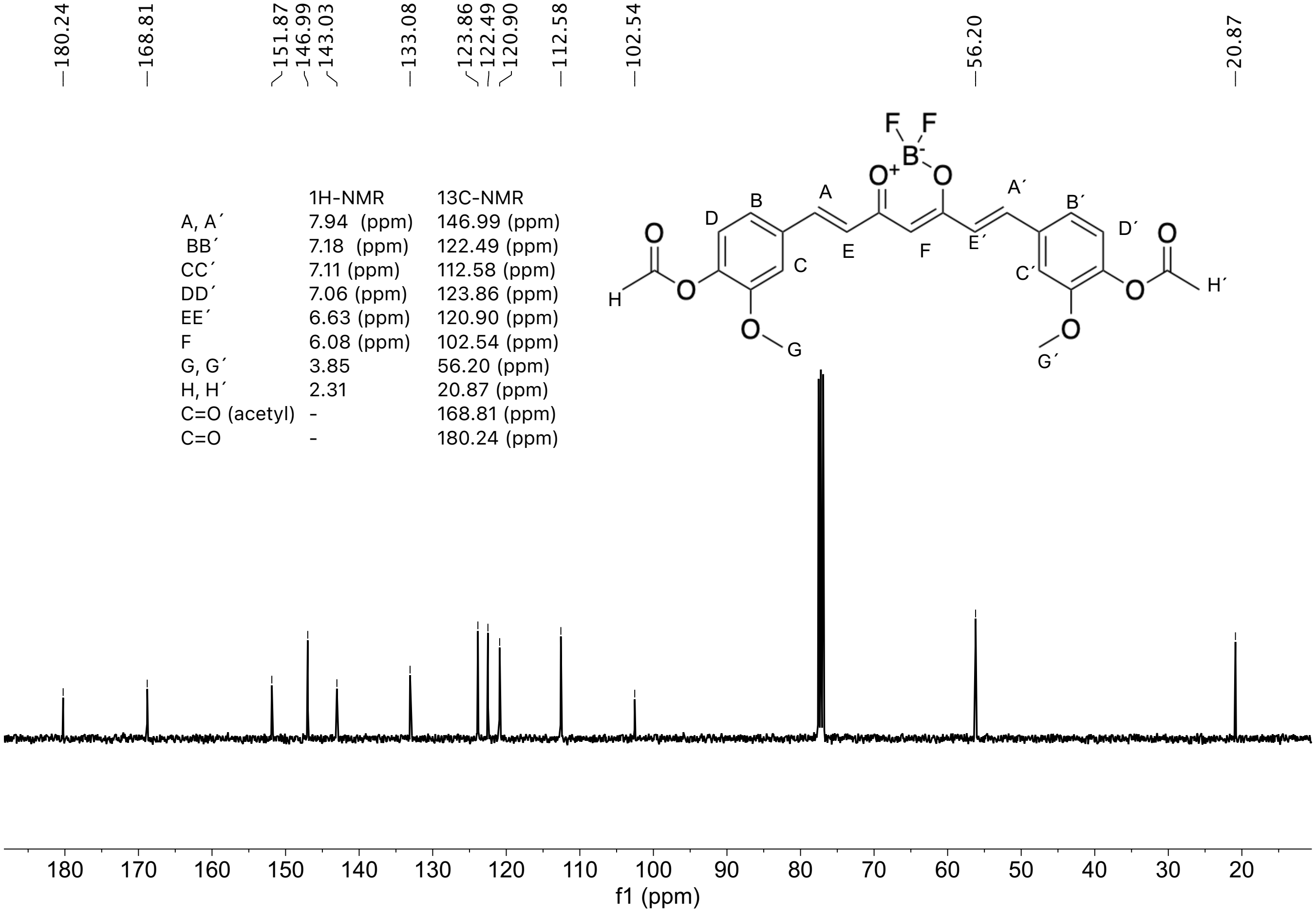

Figure S6. ^{13}C-NMR spectrum of compound 1, $CDCl_3$, 100MHz.

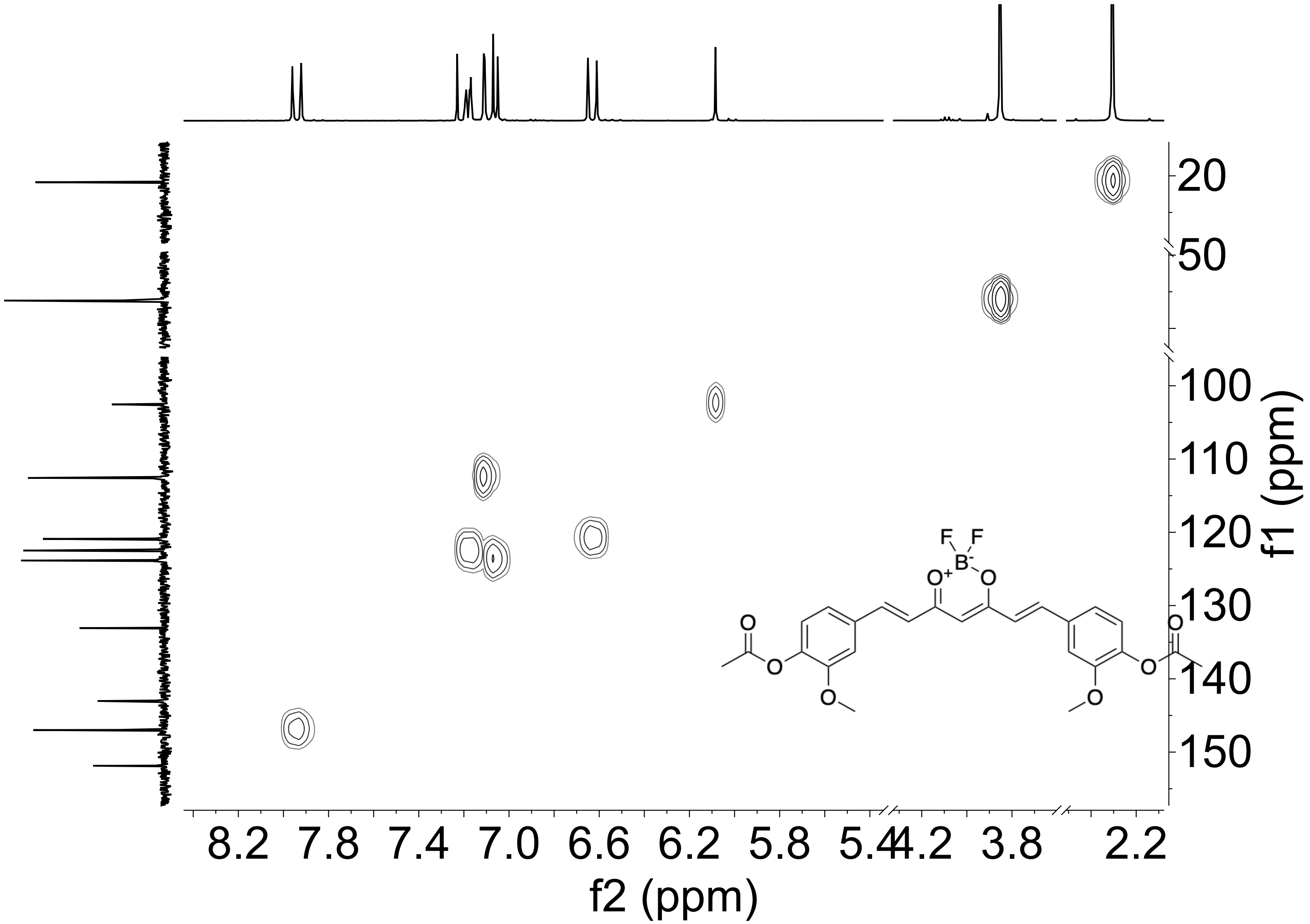

Figure S7. HSQC spectrum of compound 1, $CDCl_3$, 400MHz.

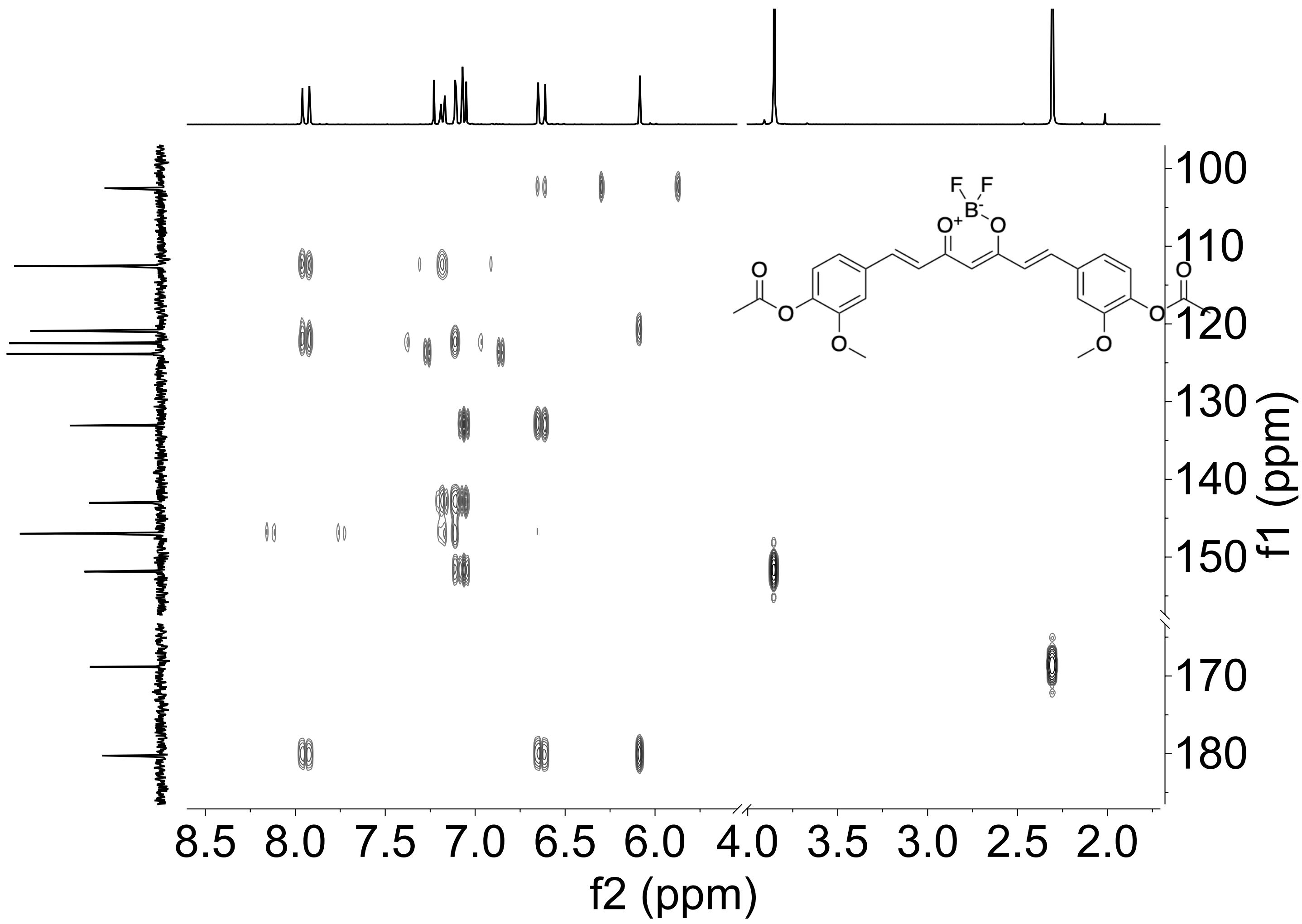

Figure S8. HMBC spectrum of compound 1, $CDCl_3$, 400MHz.

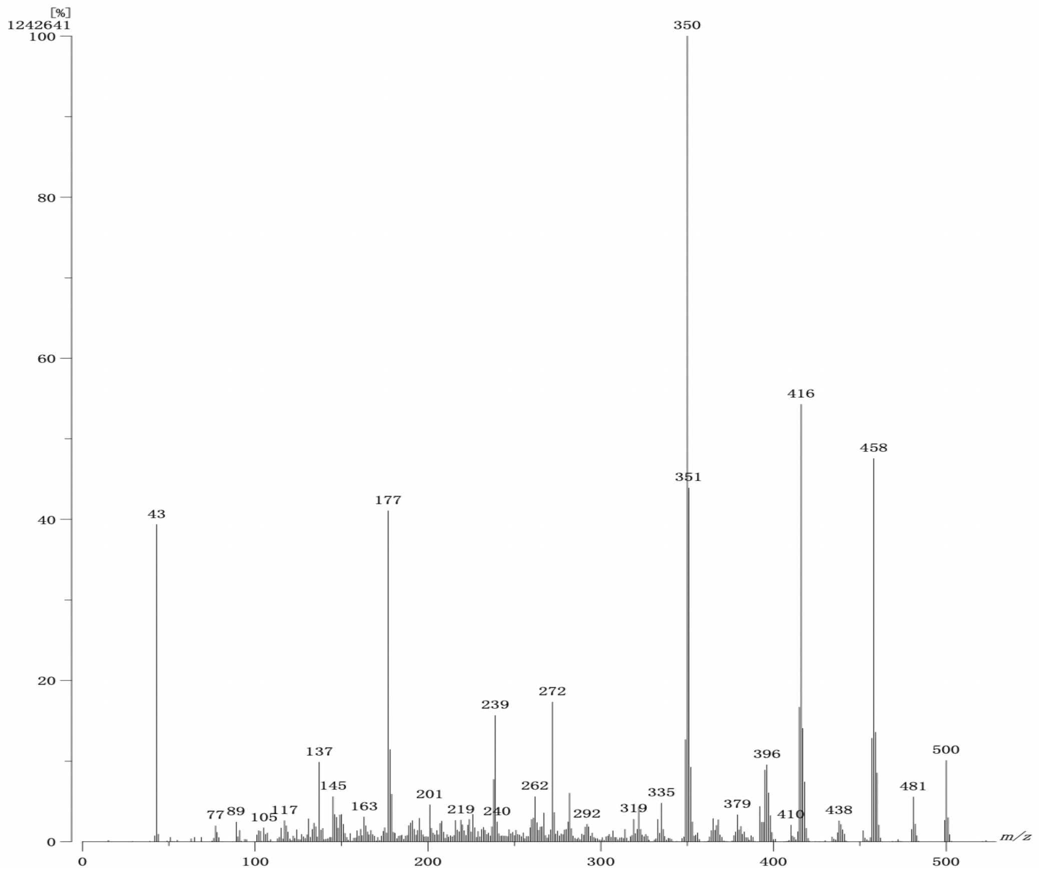

Figure S9. Mass spectrum (IE⁺) of compound 1.

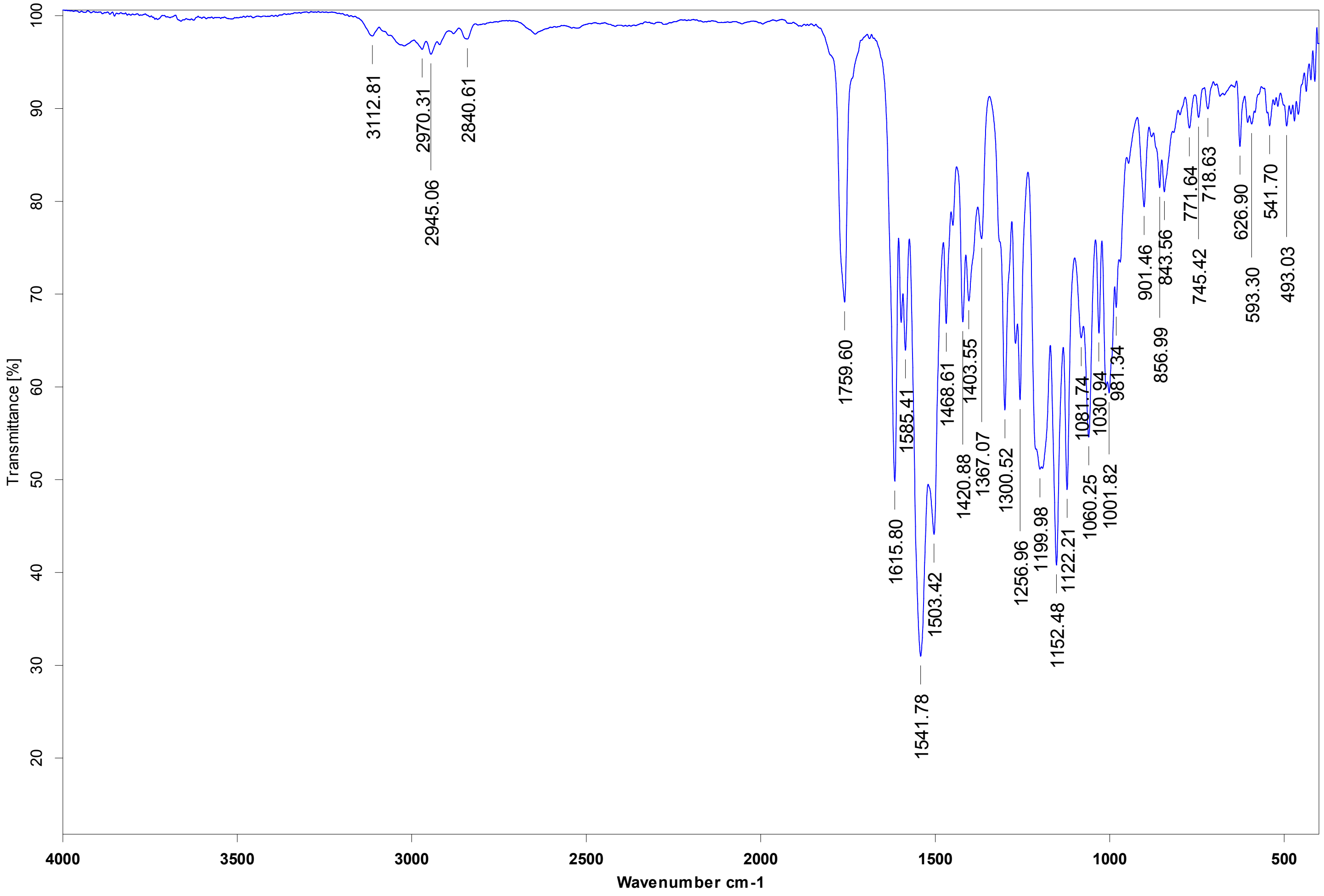

Figure S10. IR spectrum of compound 1.

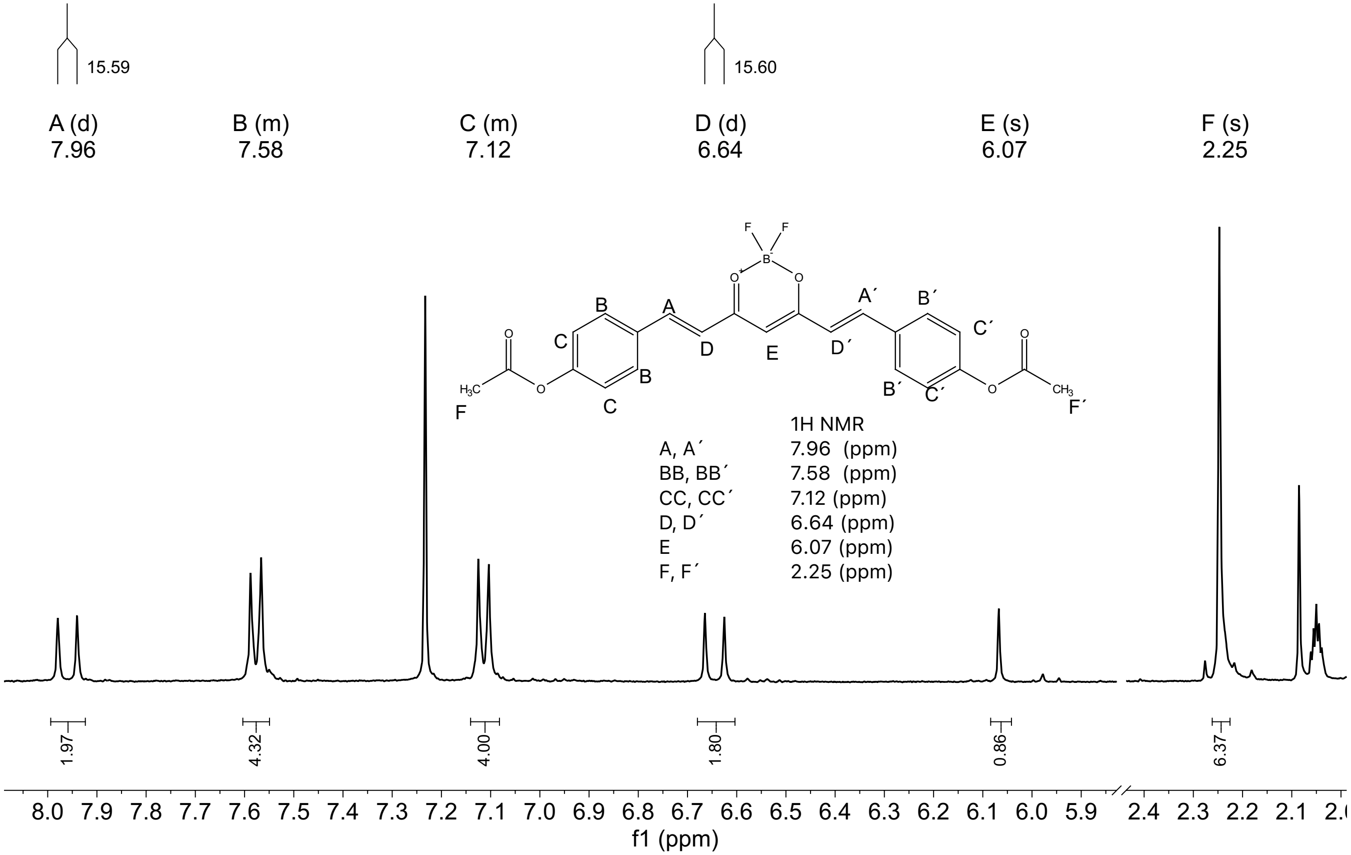

Figure S11. ^{1}H-NMR spectrum of compound 2, $CDCl_3$ + Acetone-*d6*, 400MHz.

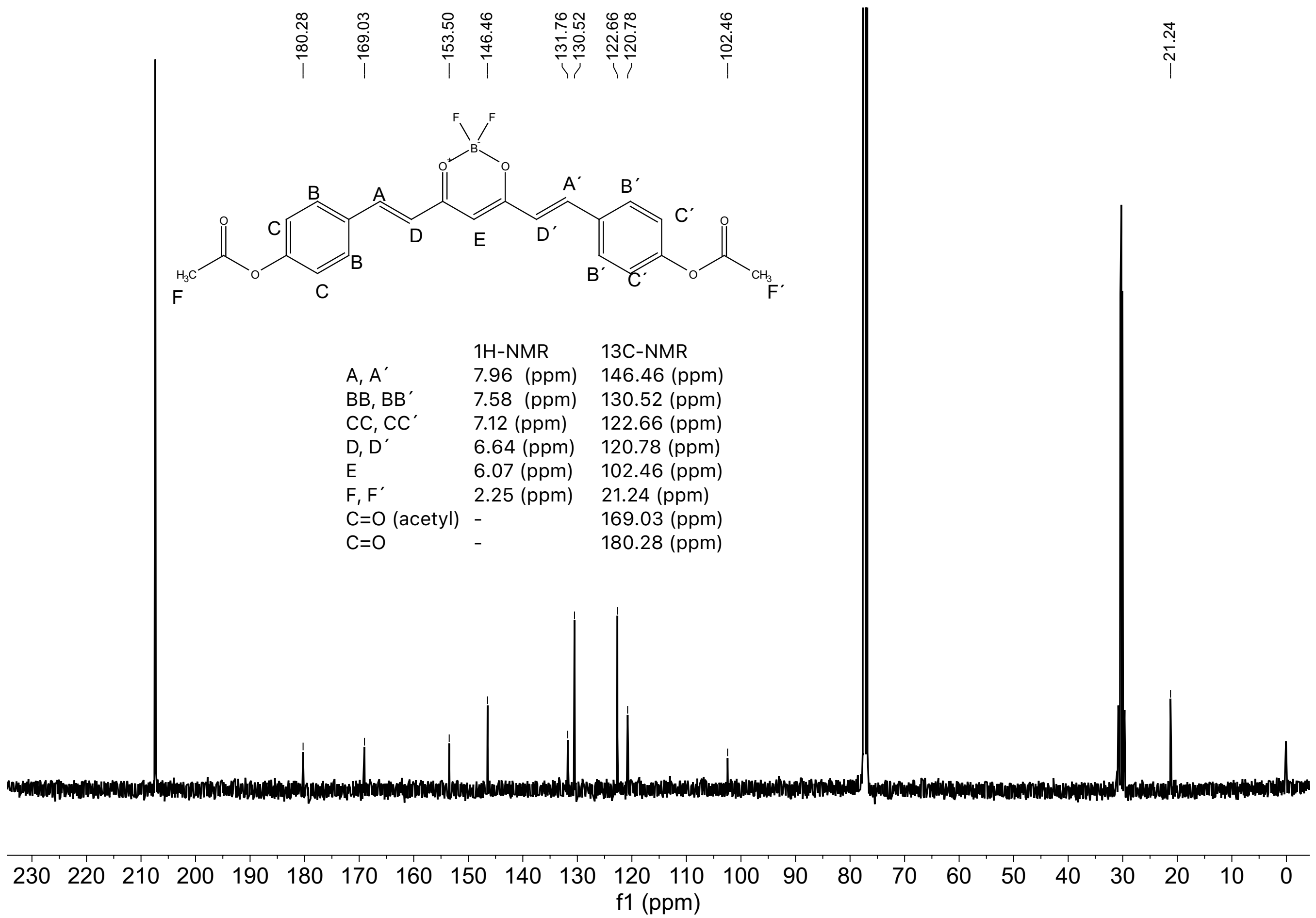

Figure S12. ^{13}**C-NMR spectrum of compound 2,** $CDCl_3$ **+ Acetone-*d6*, 100MHz.**

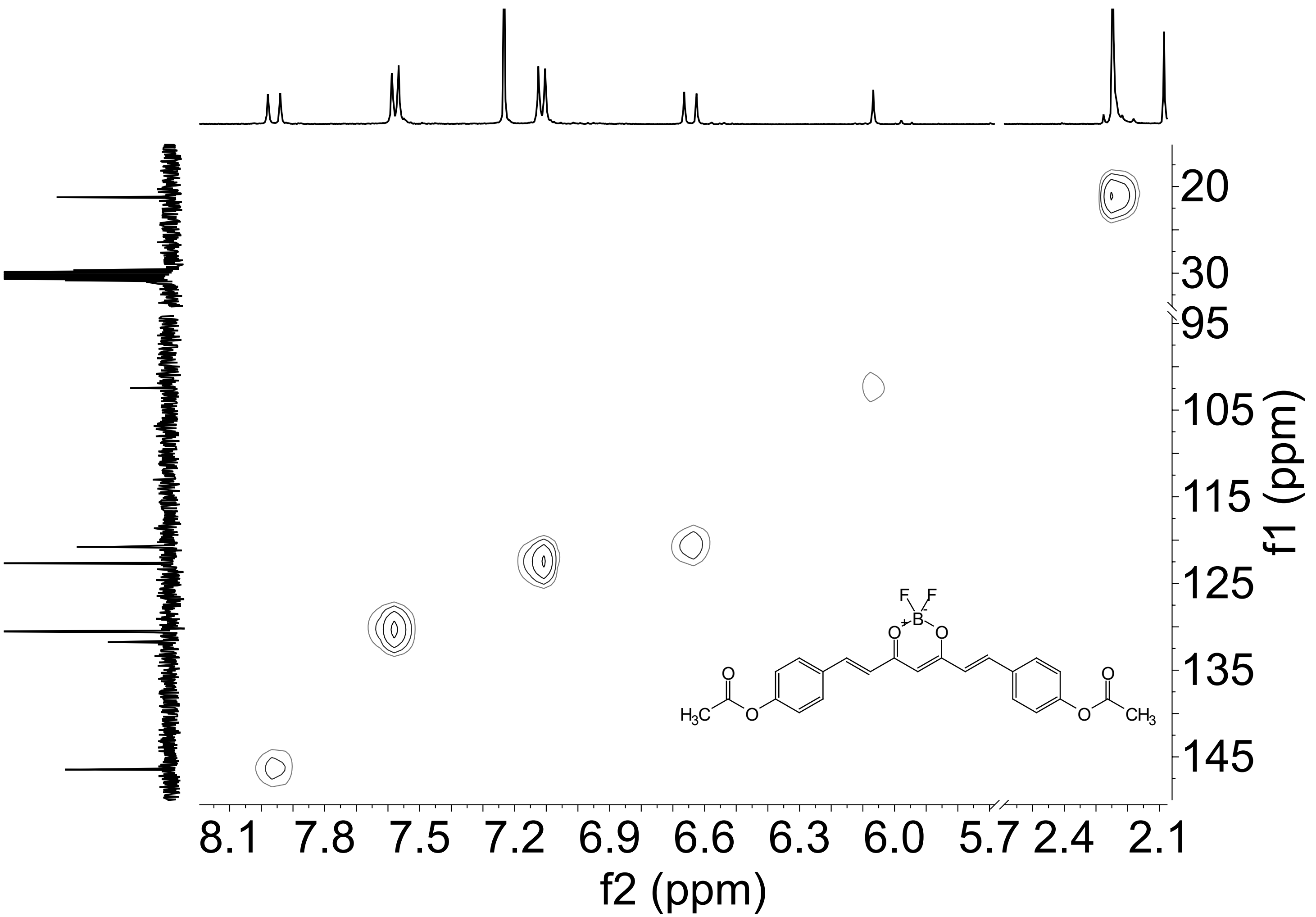

Figure S13. HSQC spectrum of compound 2, $CDCl_3$ + Acetone-*d6*, 400MHz.

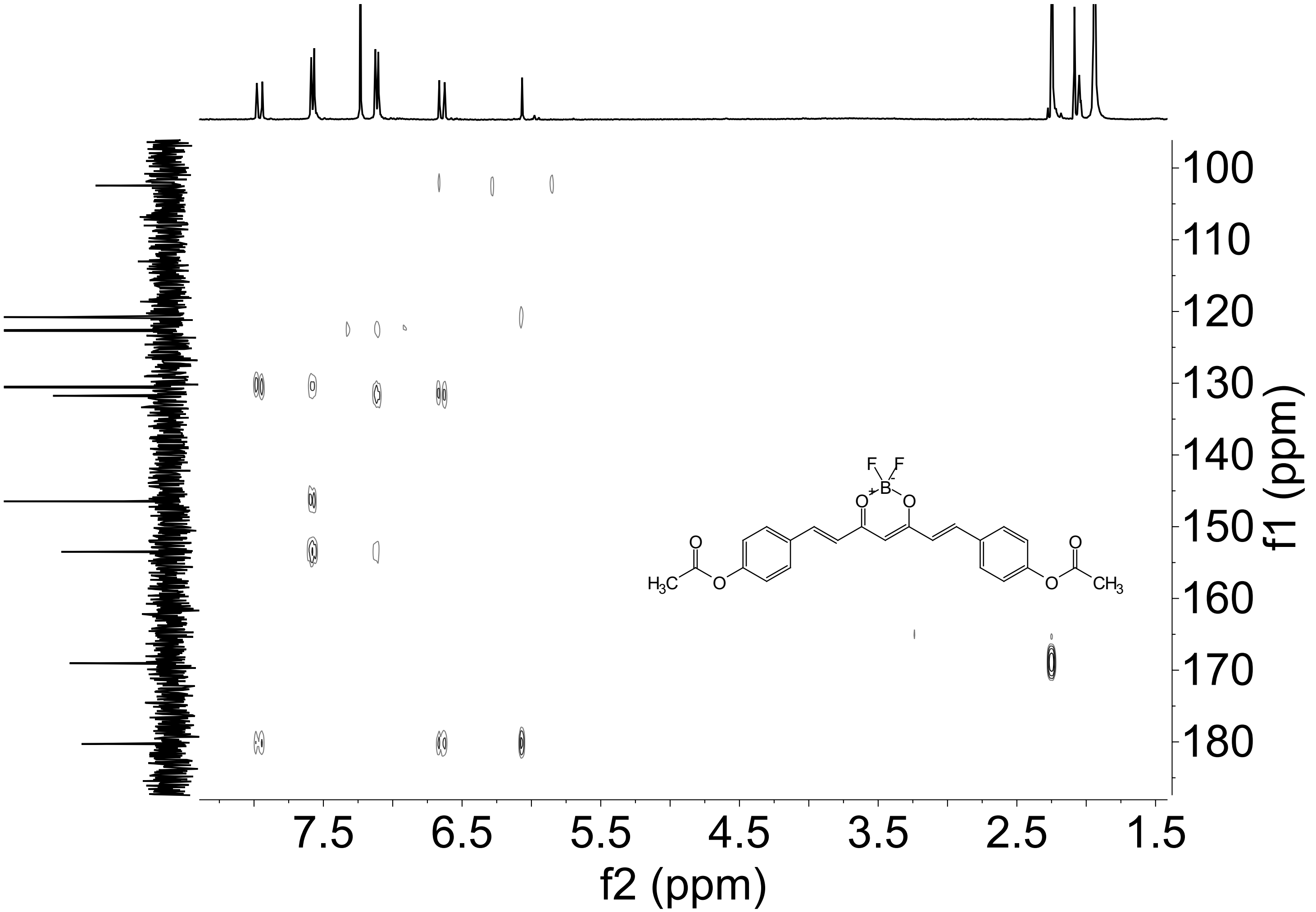

Figure S14. HMBC spectrum of compound 2, CDCl3 + Acetone-*d6*, 400MHz.

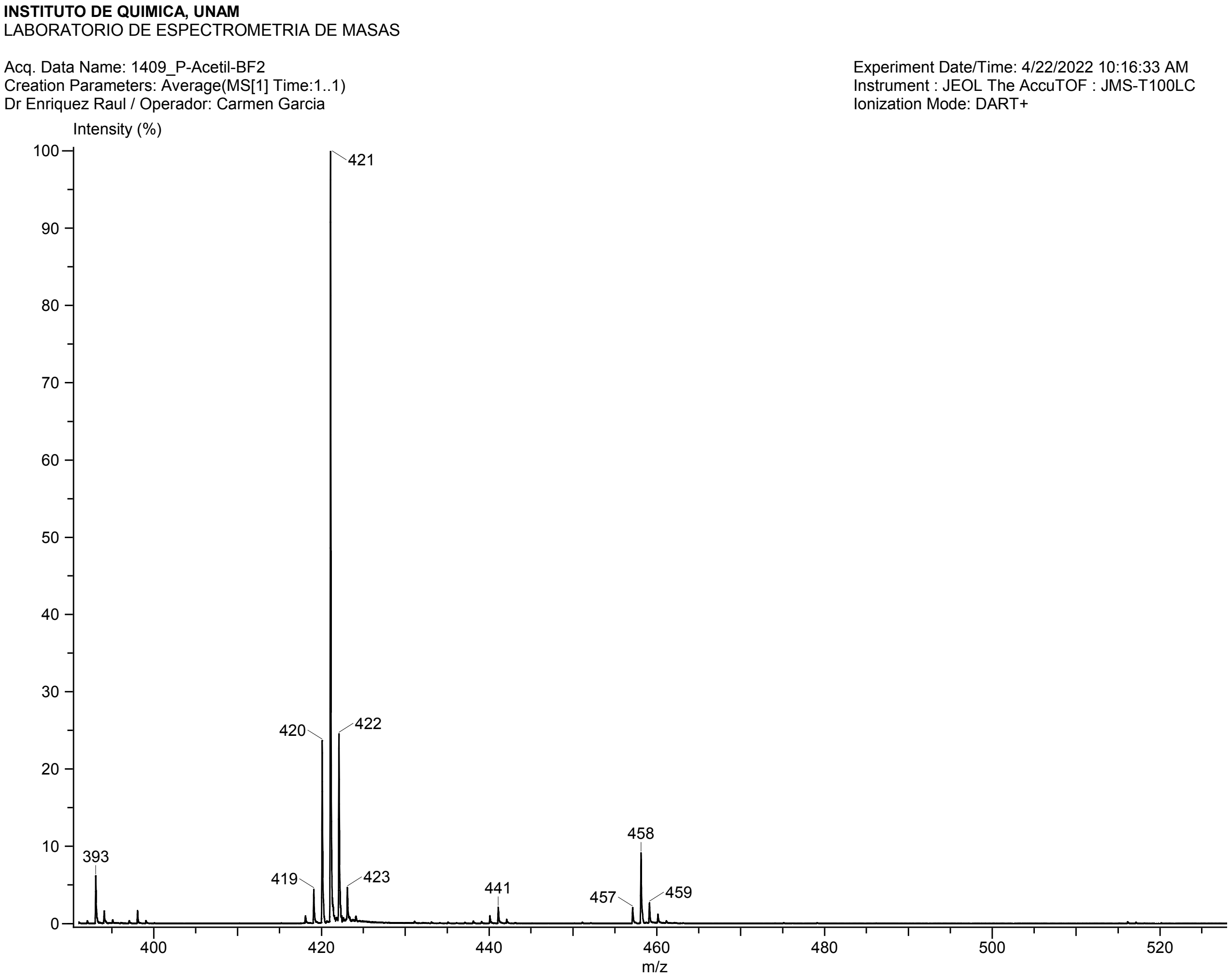

Figure S15. Mass spectrum (IE$^+$) of compound 2.

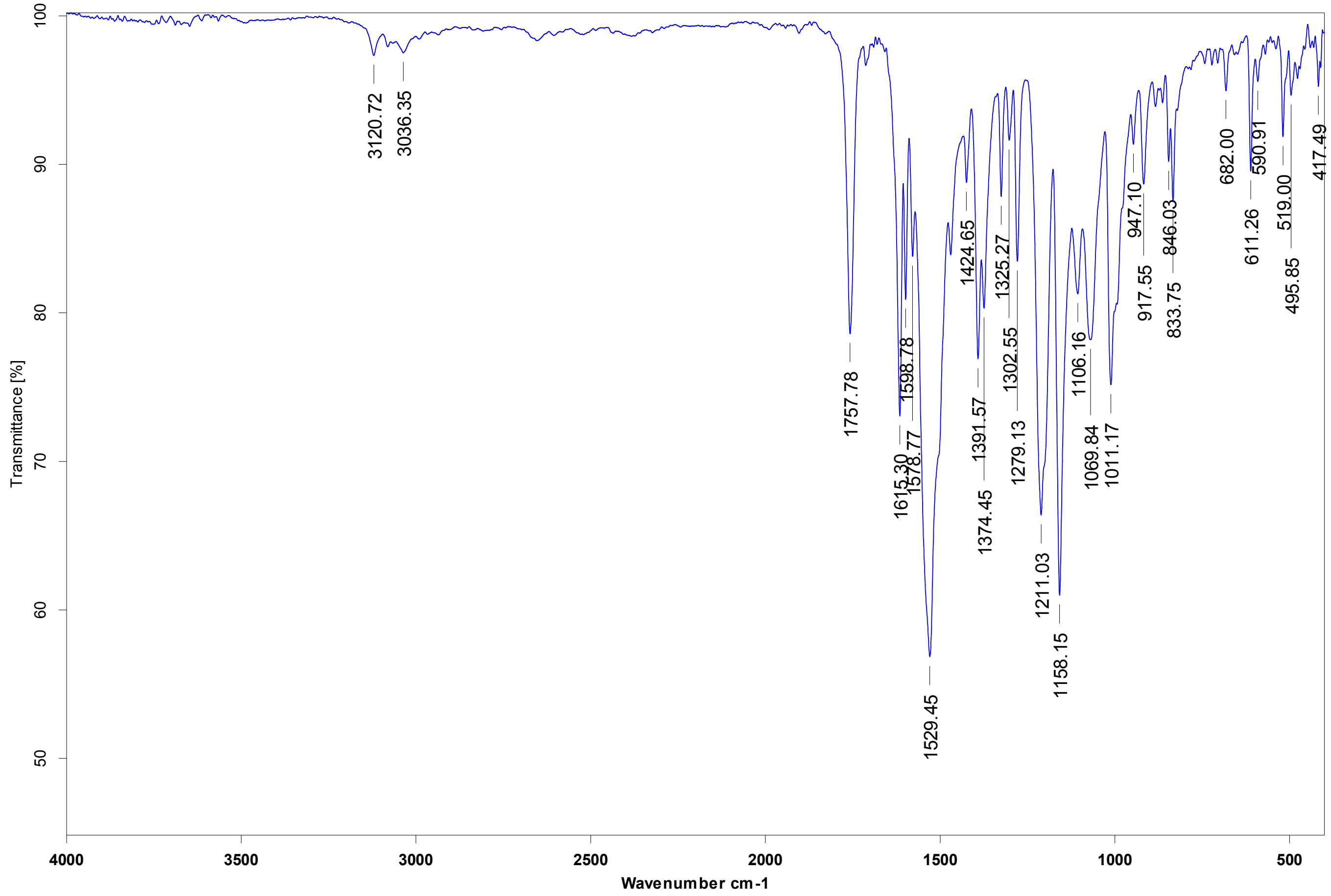

Figure S16. IR spectrum of compound 2.

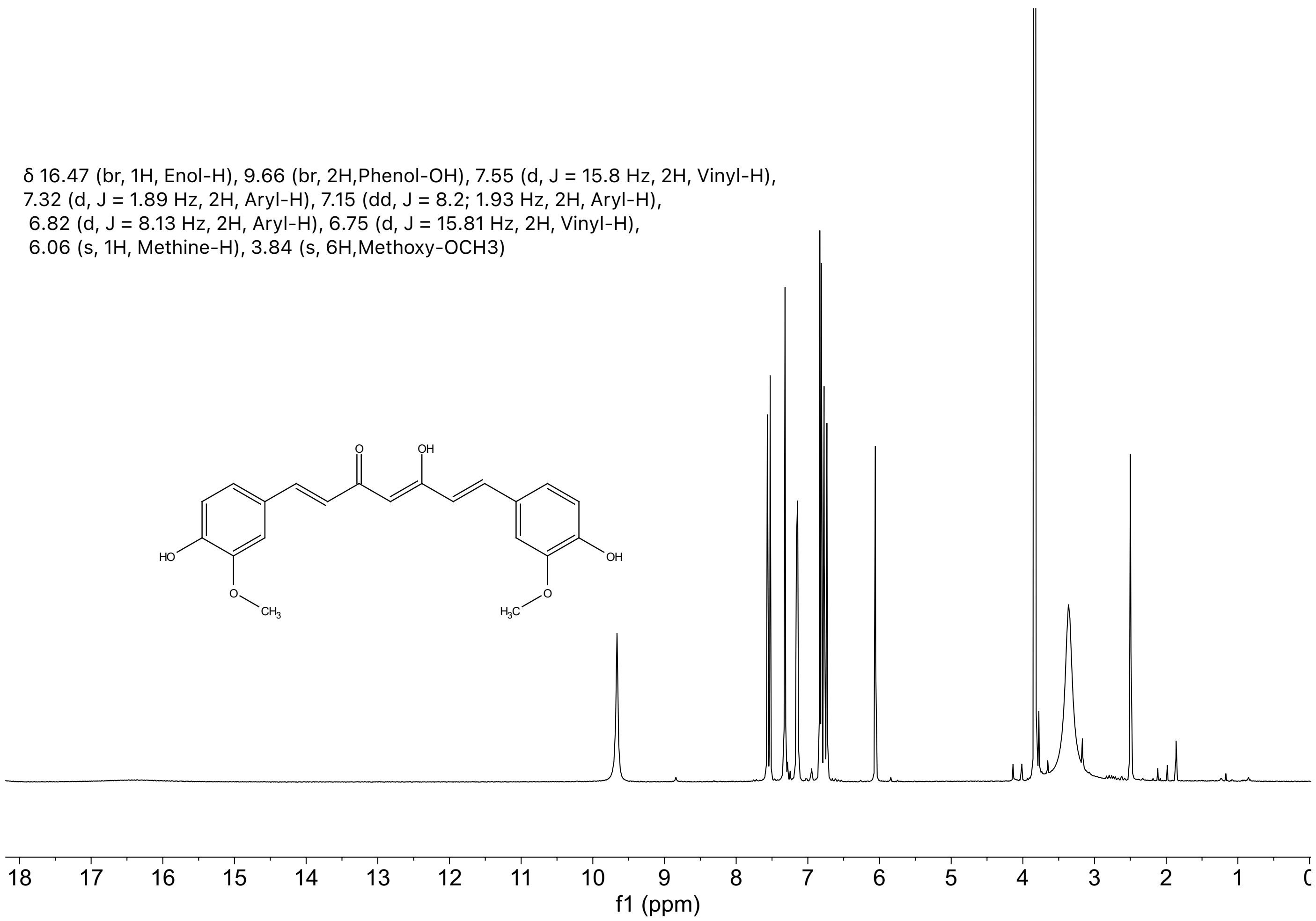

Figure S17. ^{1}H-NMR spectrum of compound 3 (Curcumin, CUR), DMSO-*d6*, 400MHz.

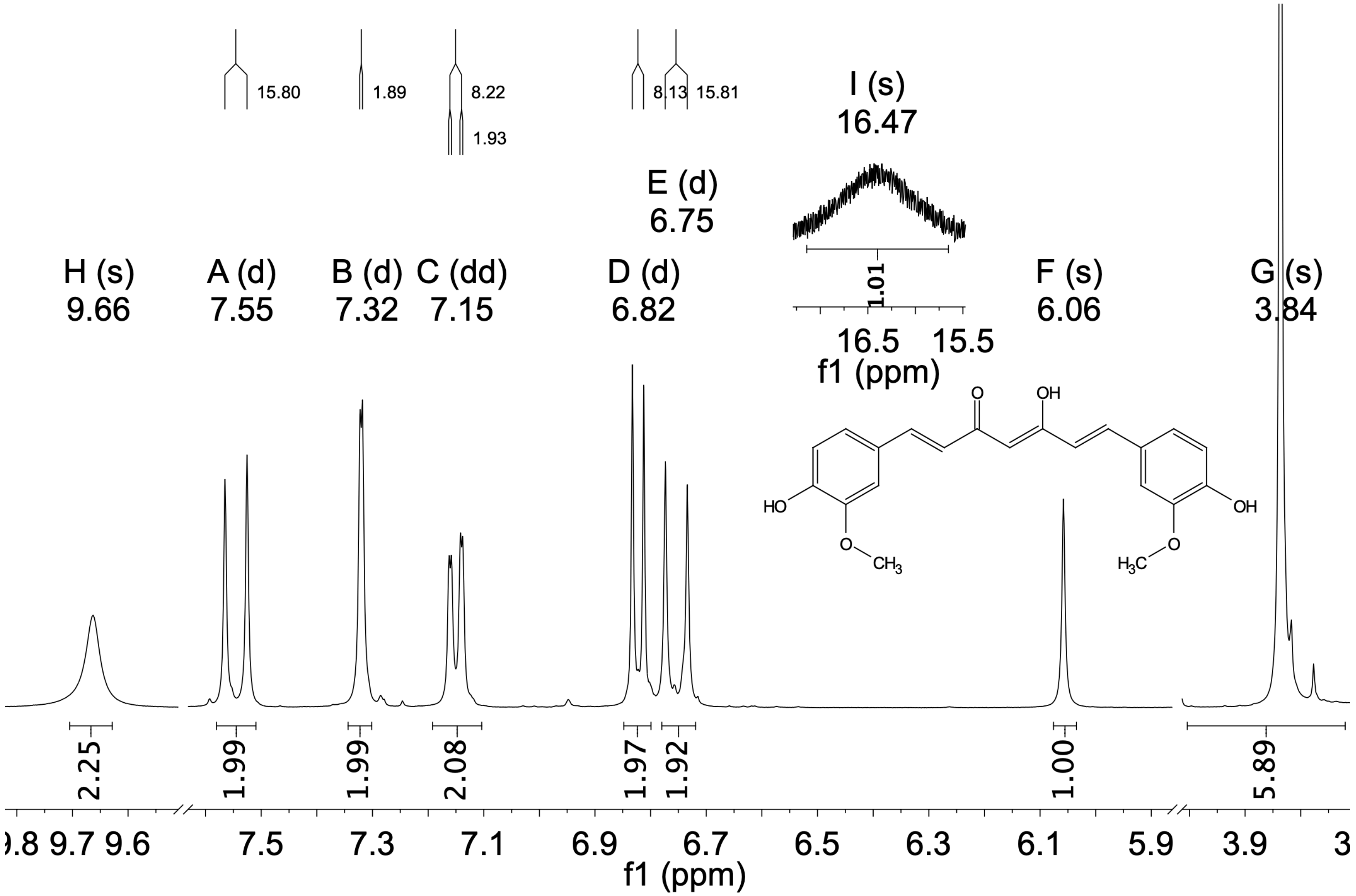

Figure S18. **^{1}H-NMR spectrum (aromatic region) of compound 3, DMSO-*d6*, 400MHz.**

δ 16.47 (br, 1H, Enol-H), 9.66 (br, 2H,Phenol-OH), 7.55 (d, J = 15.8 Hz, 2H, Vinyl-H),
7.32 (d, J = 1.89 Hz, 2H, Aryl-H), 7.15 (dd, J = 8.2; 1.93 Hz, 2H, Aryl-H),
6.82 (d, J = 8.13 Hz, 2H, Aryl-H), 6.75 (d, J = 15.81 Hz, 2H, Vinyl-H),
6.06 (s, 1H, Methine-H), 3.84 (s, 6H,Methoxy-OCH3);
13C NMR (100 MHz, DMSO-d6):
δ 183.22 (C=O), 149.36 (C-OH), 148.00 (Caryl), 140.72 (Cvinyl-H),
126.34 (Caryl), 123.14 (Caryl-H), 121.10 (Cvinyl-H), 115.70 (Caryl-H),
111.33 (Caryl-H), 100.85 (Cmethine-H), 55.69 (-OCH3).

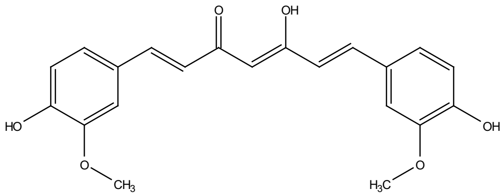

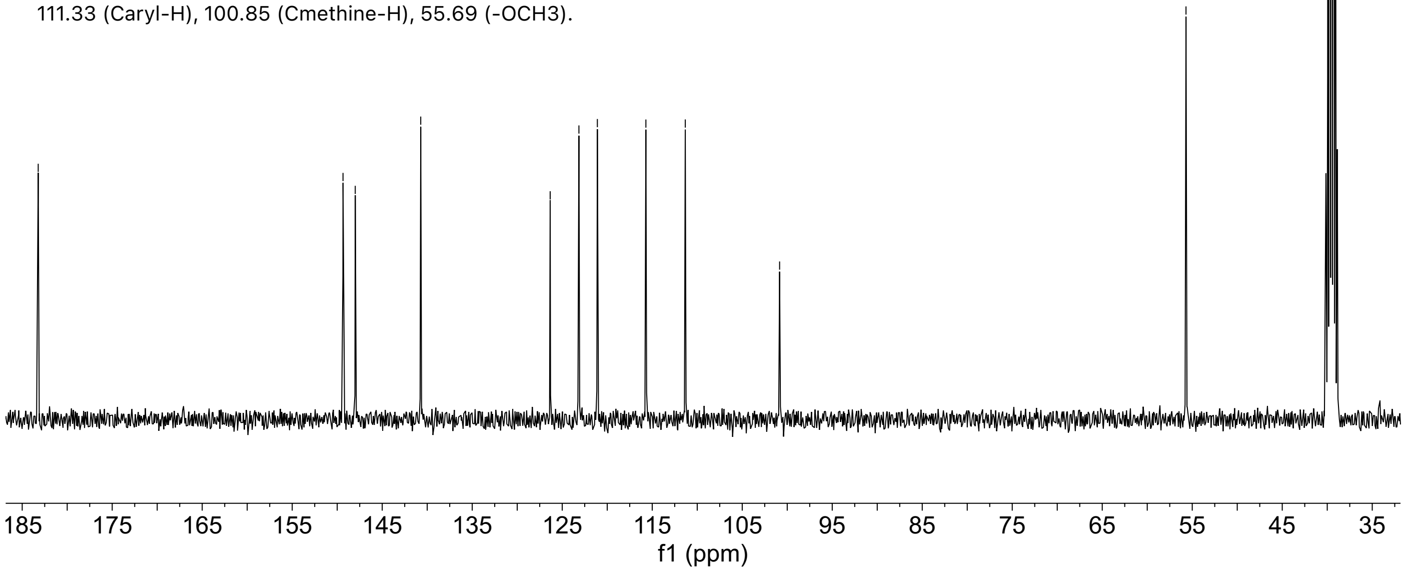

Figure S19. ^{13}C-NMR spectrum of compound 3, DMSO-*d6*, 100MHz.

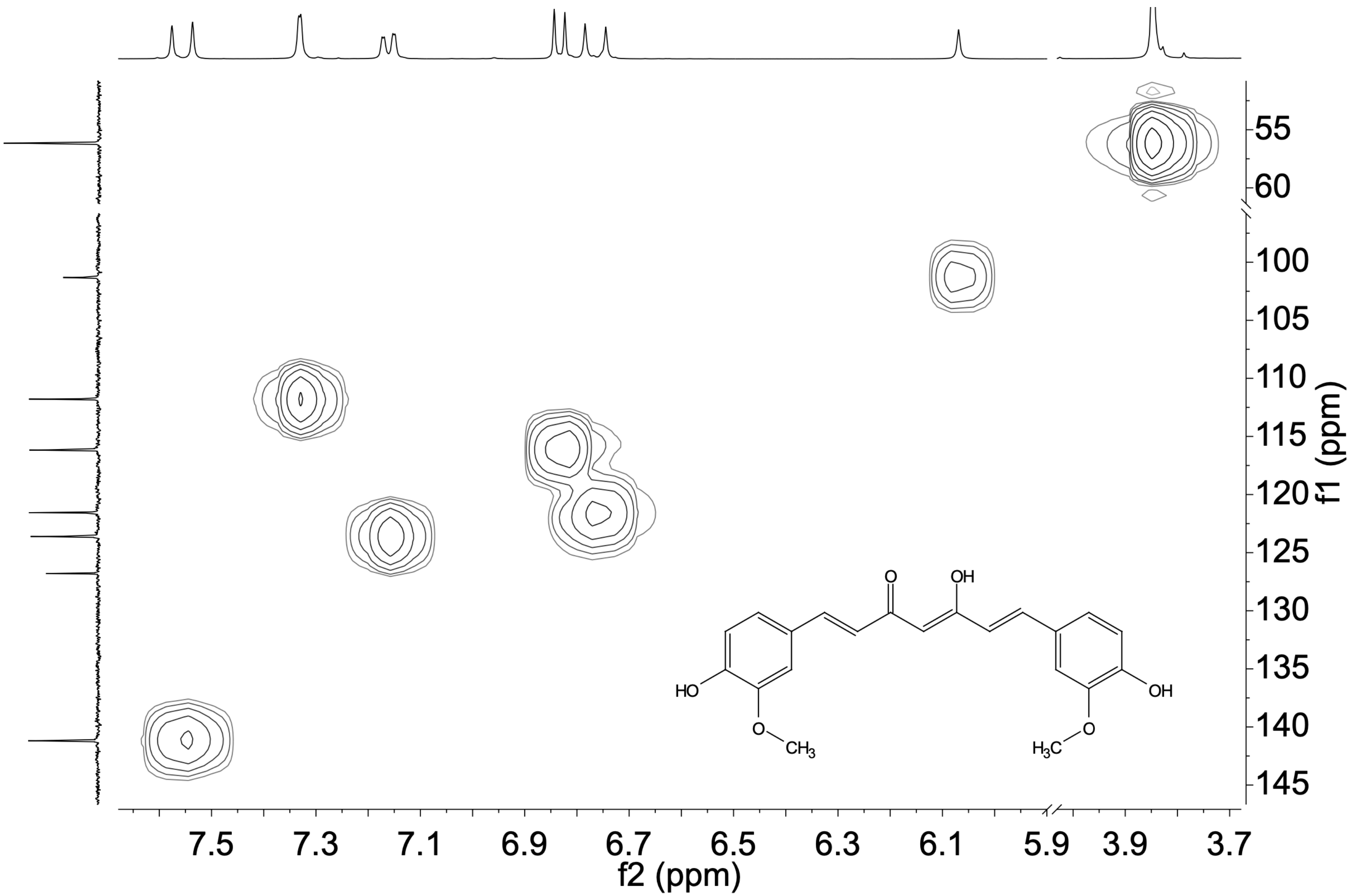

Figure S20. HSQC spectrum of compound 3, DMSO-*d6*, 400MHz.

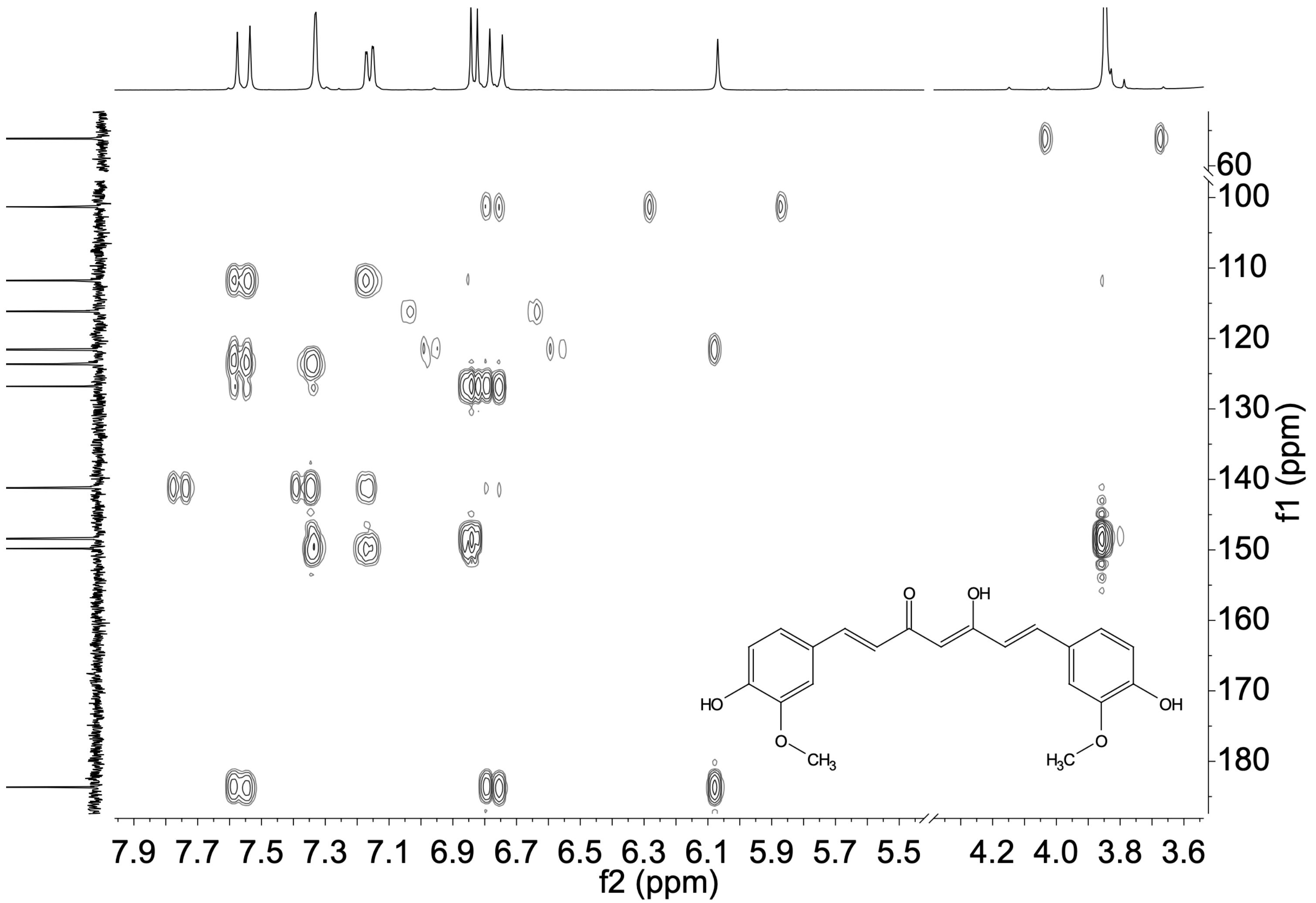

Figure S21. HMBC spectrum of compound 3, DMSO-*d6*, 400MHz.

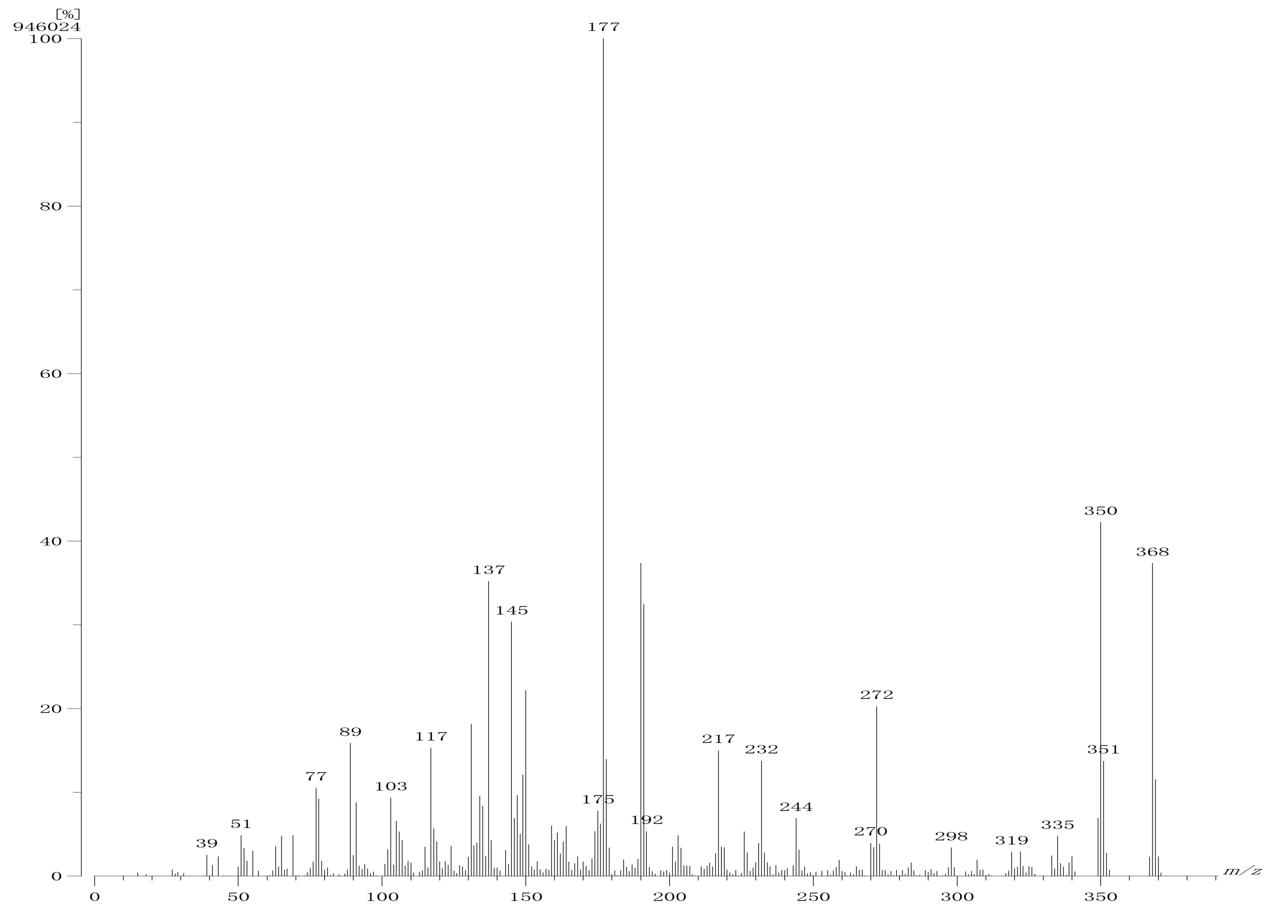

Figure S22. Mass spectrum (EI$^+$) of compound 3.

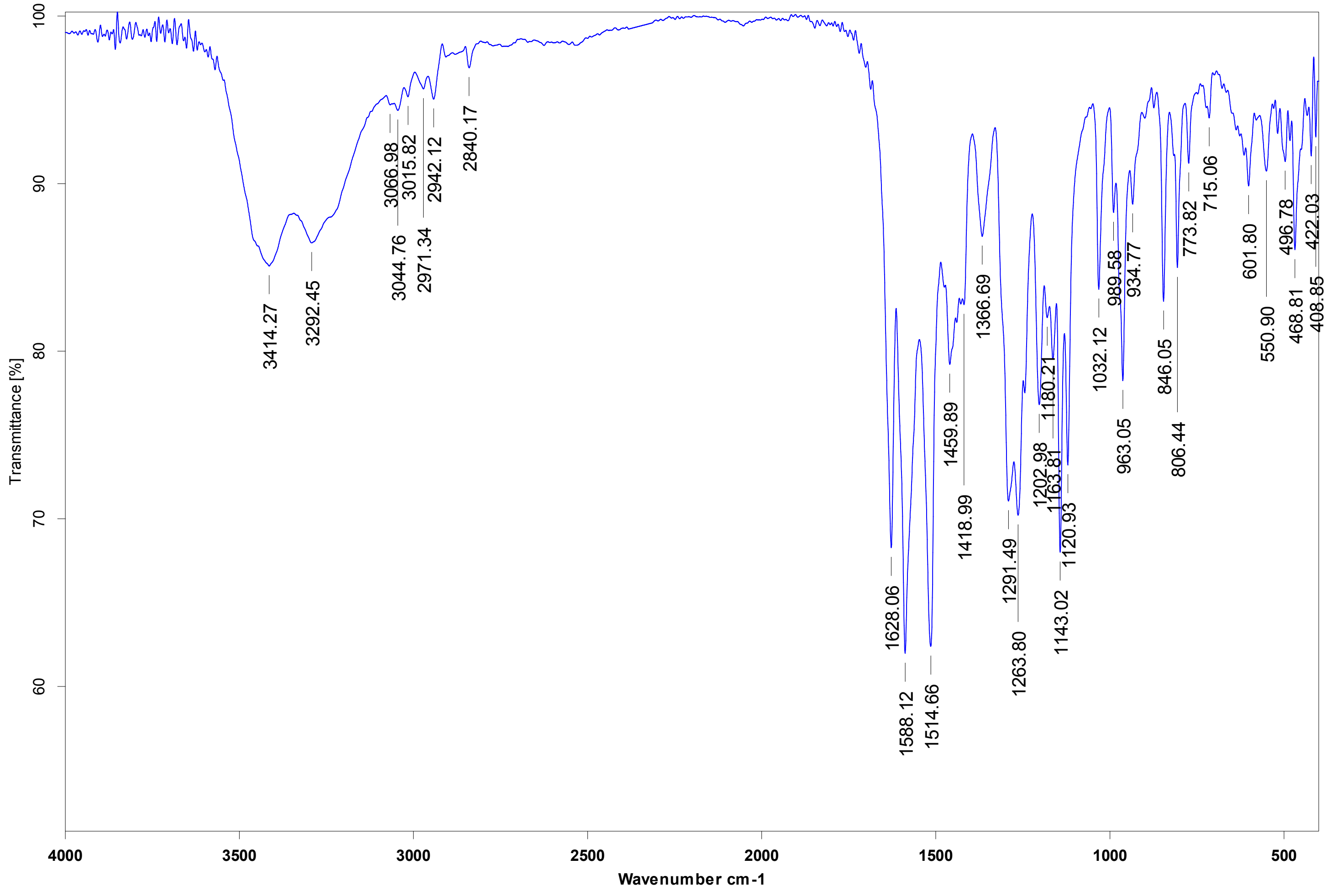

Figure S23. IR spectrum of compound 3.

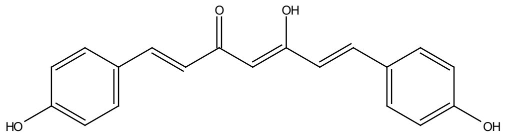

1H NMR (400 MHz, DMSO-d6): δ 10.04 (br, 2H,-OH), 7.55 (d, J = 15.8 Hz, 2H, Vinyl-H), 7.55 (m, 4H, Aryl-H), 6.82 (m, 4H, Aryl-H), 6.69 (d, J = 15.8 Hz, 2H, Vinyl-H), 6.04 (s, 1H, Methine-H);

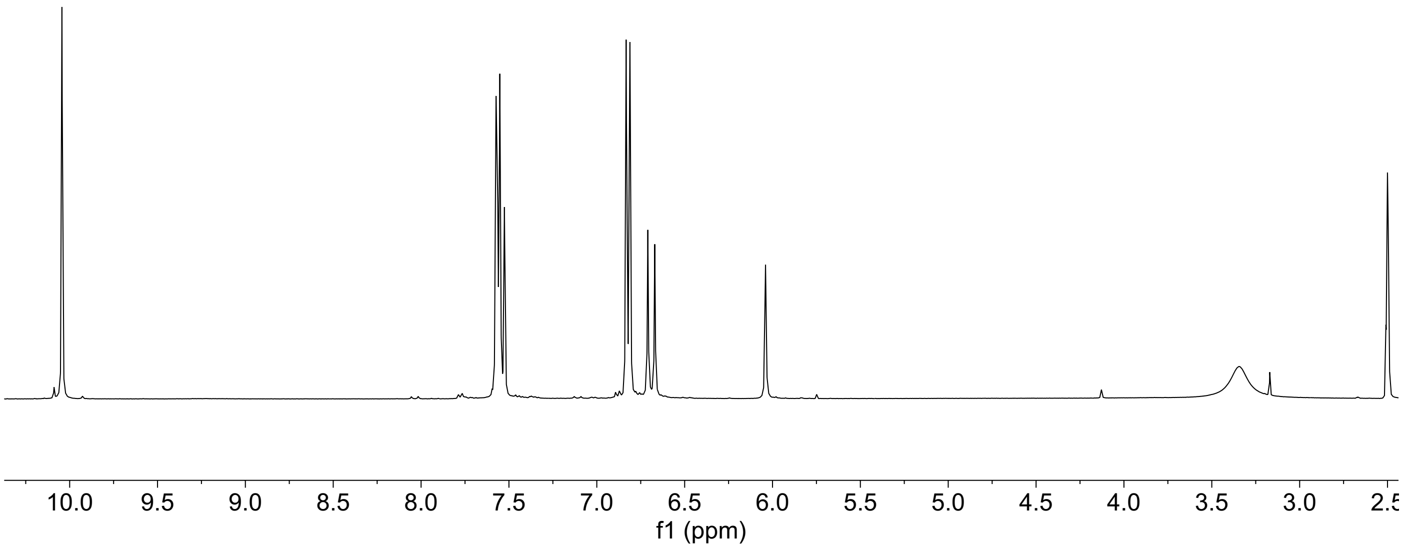

Figure S24. ^{1}H-NMR spectrum of compound 4 (Bis-demethoxycurcumin, BDMC), DMSO-*d6*, 400MHz.

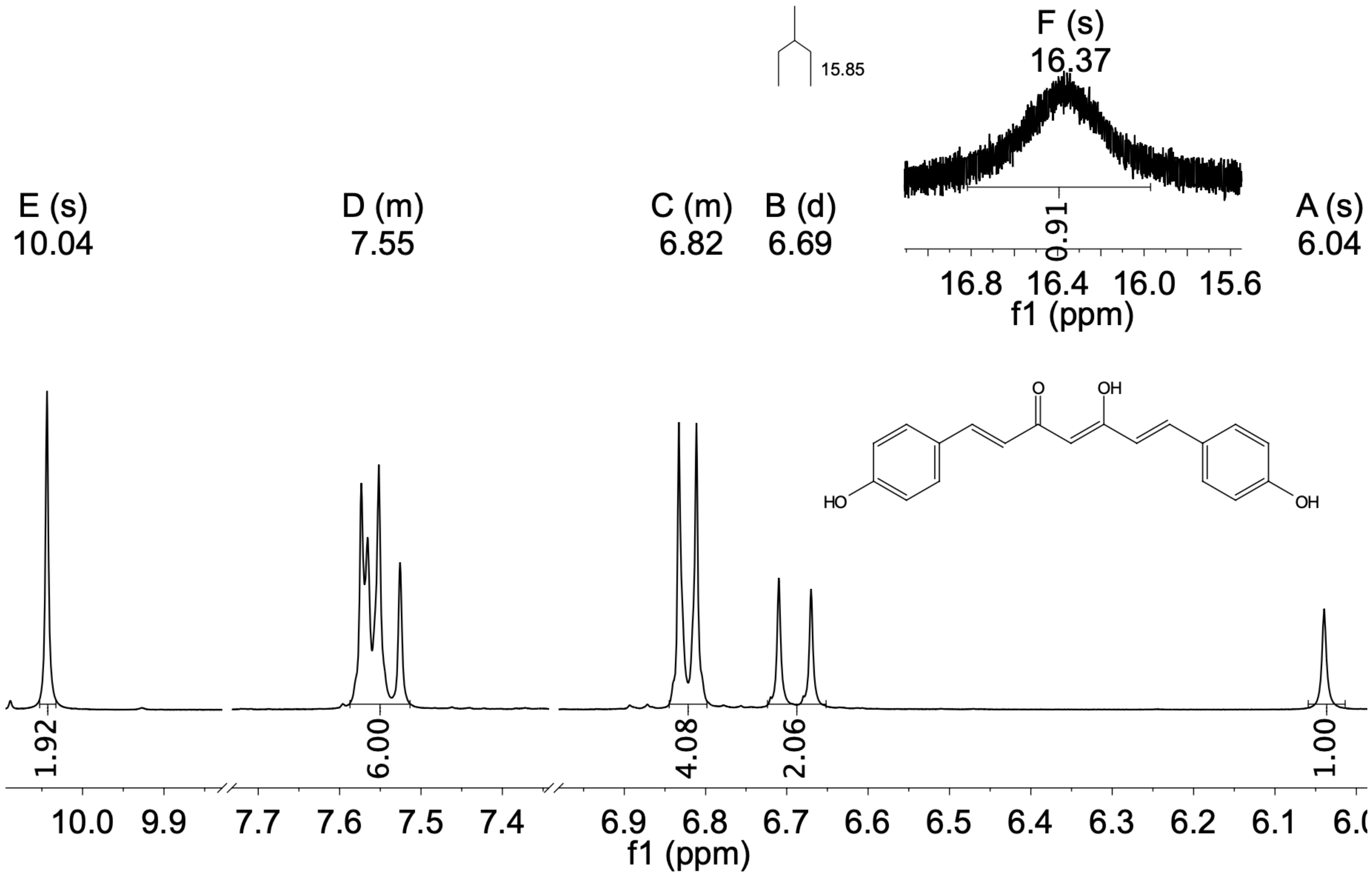

Figure S25. ^{1}H-NMR spectrum (aromatic region) of compound 4, DMSO-*d6*, 400MHz.

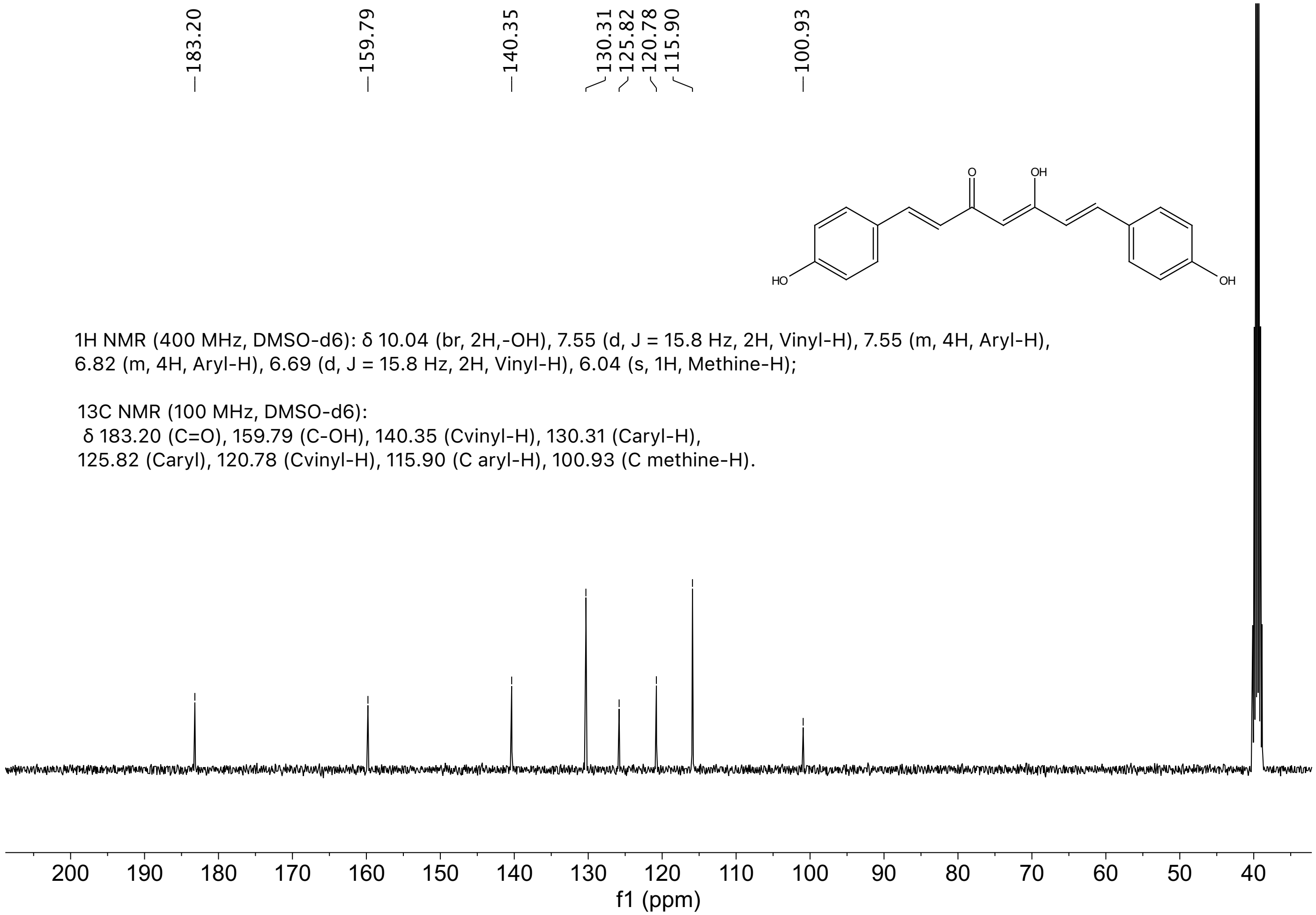

1H NMR (400 MHz, DMSO-d6): δ 10.04 (br, 2H,-OH), 7.55 (d, J = 15.8 Hz, 2H, Vinyl-H), 7.55 (m, 4H, Aryl-H), 6.82 (m, 4H, Aryl-H), 6.69 (d, J = 15.8 Hz, 2H, Vinyl-H), 6.04 (s, 1H, Methine-H);

13C NMR (100 MHz, DMSO-d6):
δ 183.20 (C=O), 159.79 (C-OH), 140.35 (Cvinyl-H), 130.31 (Caryl-H), 125.82 (Caryl), 120.78 (Cvinyl-H), 115.90 (C aryl-H), 100.93 (C methine-H).

Figure S26. 13C-NMR spectrum of compound 4, DMSO-*d6*, 100 MHz.

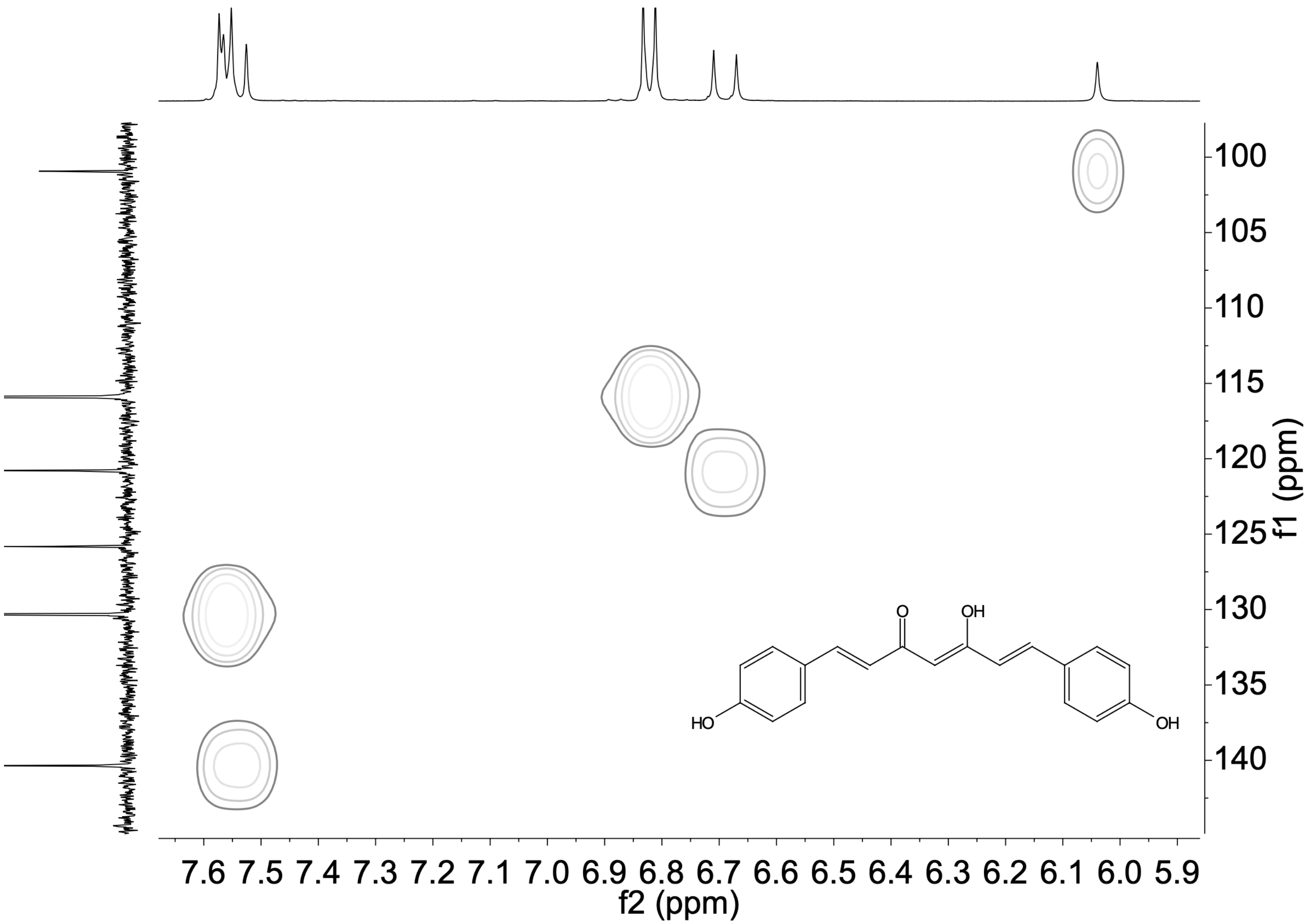

Figure S27. HSQC spectrum of compound 4, DMSO-*d6*, 400MHz.

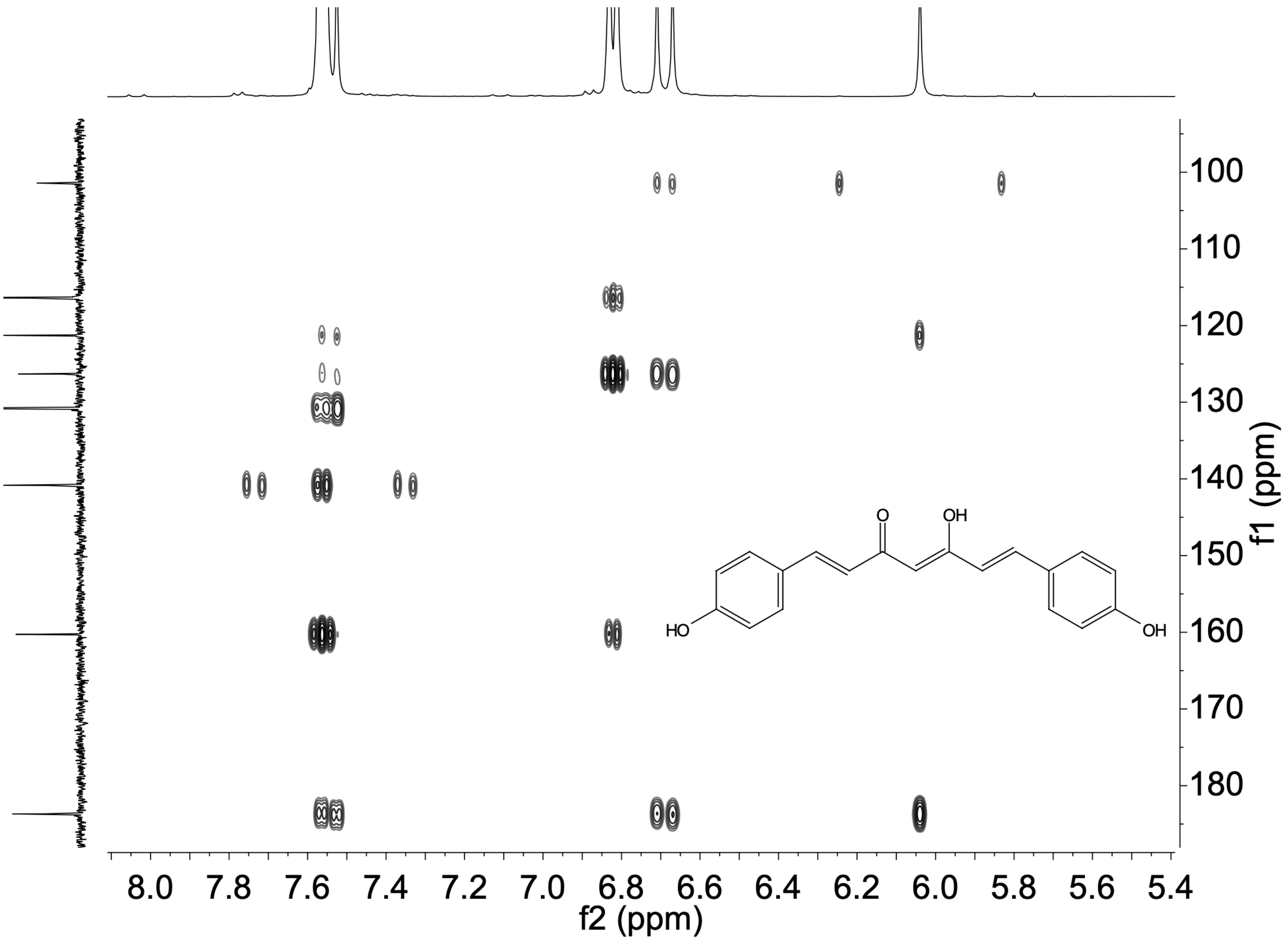

Figure S28. HMBC spectrum of compound 4, DMSO-*d6*, 400MHz.

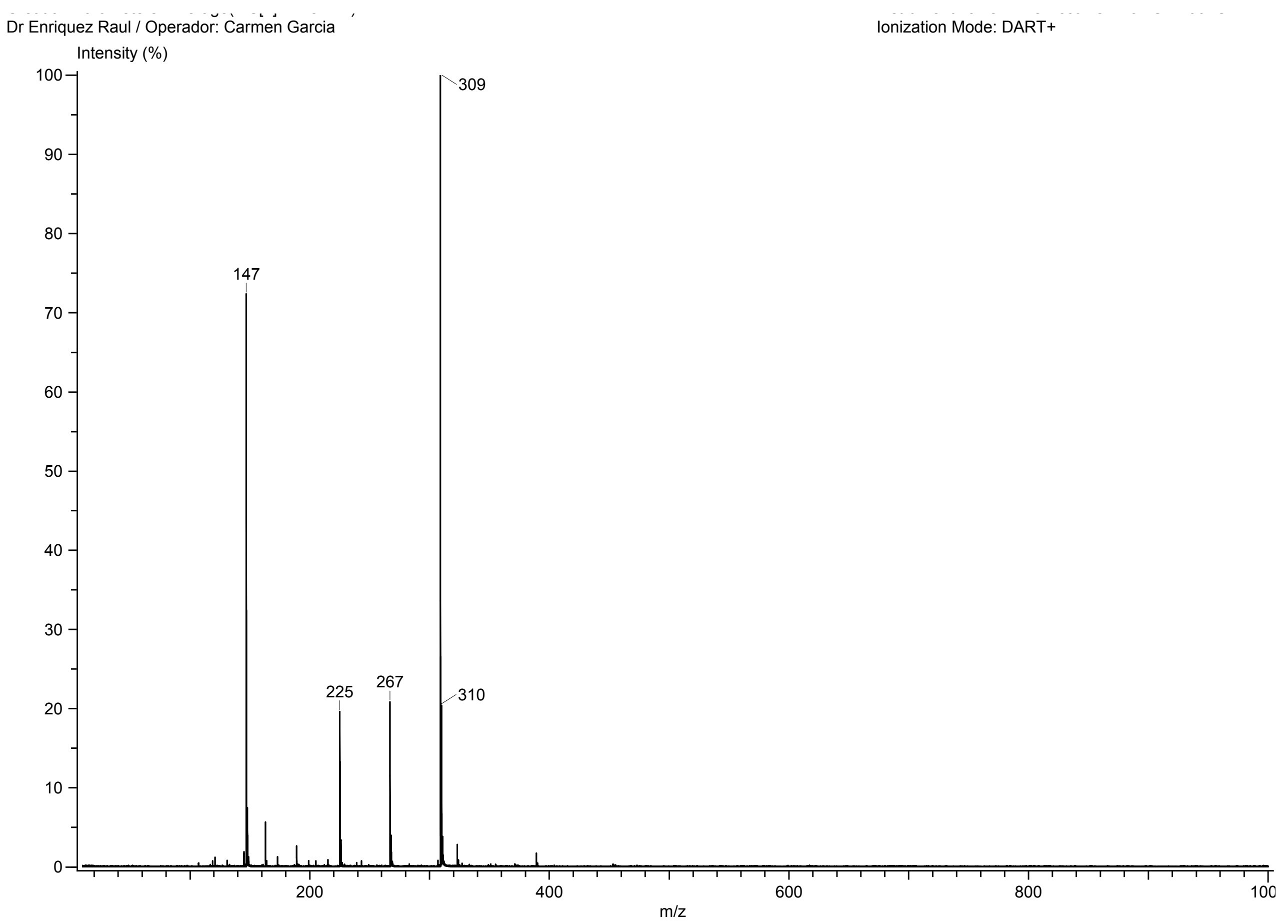

Figure S29. Mass spectrum (DART$^+$) of compound 4.

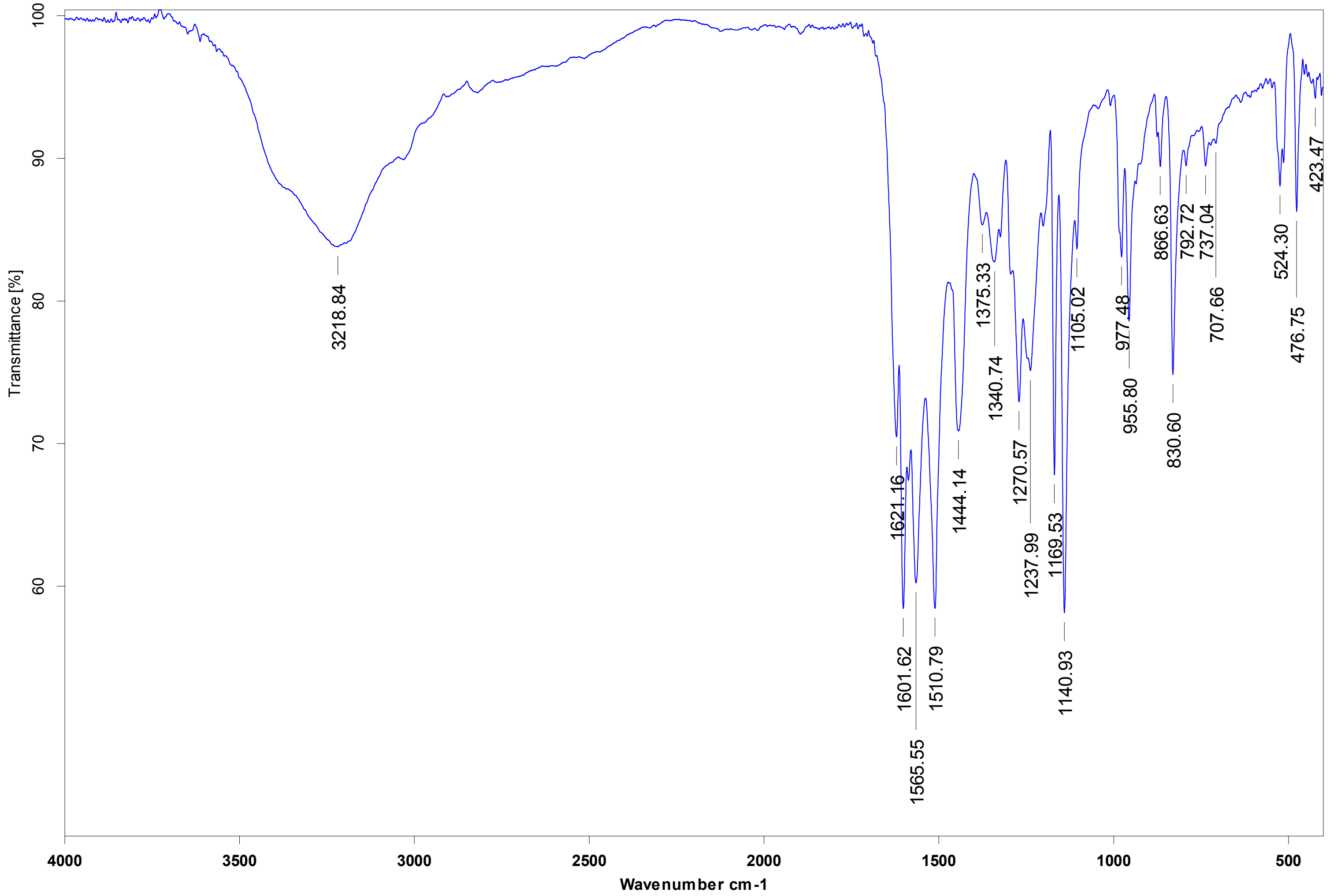

Figure S30. IR spectrum of compound 4.

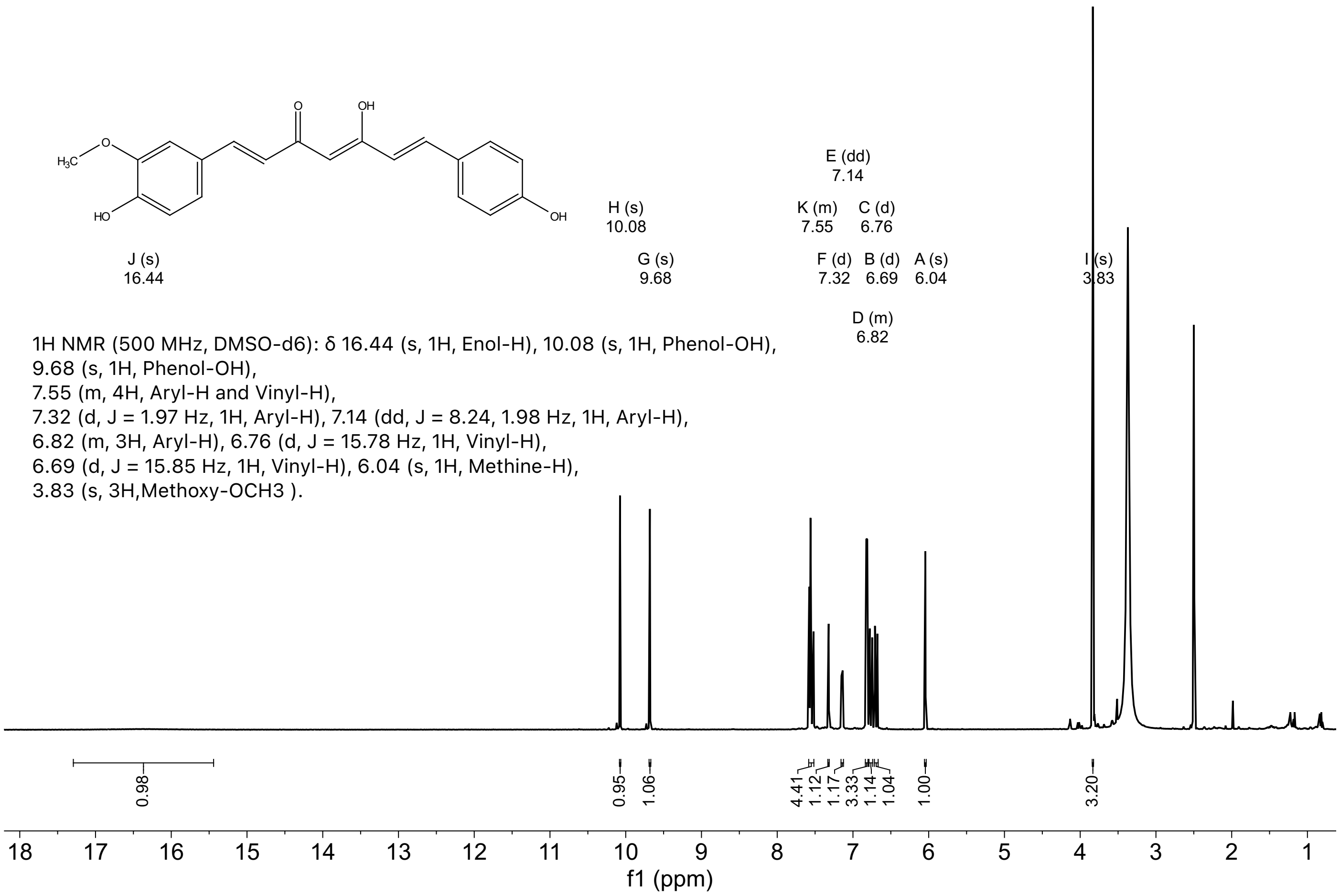

Figure S31. ^{1}H-NMR spectrum of compound 5 (Demethoxycurcumin, DMC), DMSO-*d6*, 400MHz.

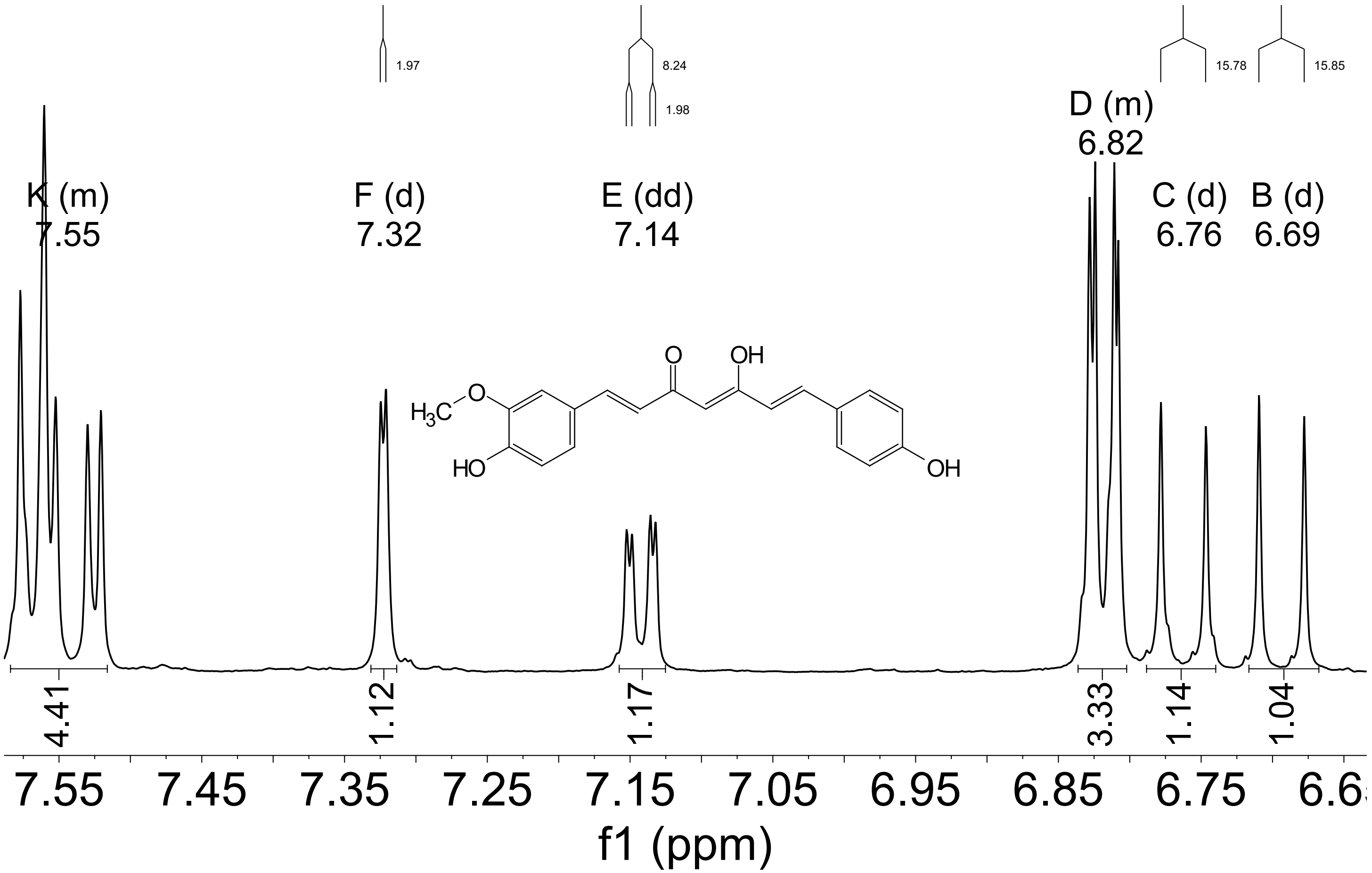

Figure S32. ^{1}H-NMR spectrum (aromatic region) of compound 5, DMSO-*d6*, 400MHz.

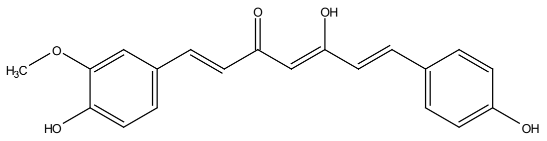

1H NMR (500 MHz, DMSO-d6): δ 16.44 (s, 1H, Enol-H), 10.08 (s, 1H, Phenol-OH),
9.68 (s, 1H, Phenol-OH),
7.55 (m, 4H, Aryl-H and Vinyl-H),
7.32 (d, J = 1.97 Hz, 1H, Aryl-H), 7.14 (dd, J = 8.24, 1.98 Hz, 1H, Aryl-H),
6.82 (m, 3H, Aryl-H), 6.76 (d, J = 15.78 Hz, 1H, Vinyl-H),
6.69 (d, J = 15.85 Hz, 1H, Vinyl-H), 6.04 (s, 1H, Methine-H),
3.83 (s, 3H,Methoxy-OCH3).

13C NMR (125 MHz, 500 MHz, DMSO-d6): δ 183.31 (C=O), 183.18 (C=O), 159.84 (C-OH),
149.37 (C-OH), 148.02 (Caryl), 140.75 (Cvinyl-H), 140.41 (Cvinyl-H),
130.39 (C aryl-H), 126.36 (Caryl), 125.85 (Caryl), 123.26 (C aryl-H),
121.06 (Cvinyl-H), 120.85 (Cvinyl-H), 115.94 (C aryl-H),
115.69 (C aryl-H),111.21(C aryl-H), 100.98 (Cmethine-H),
55.70 (-OCH3).

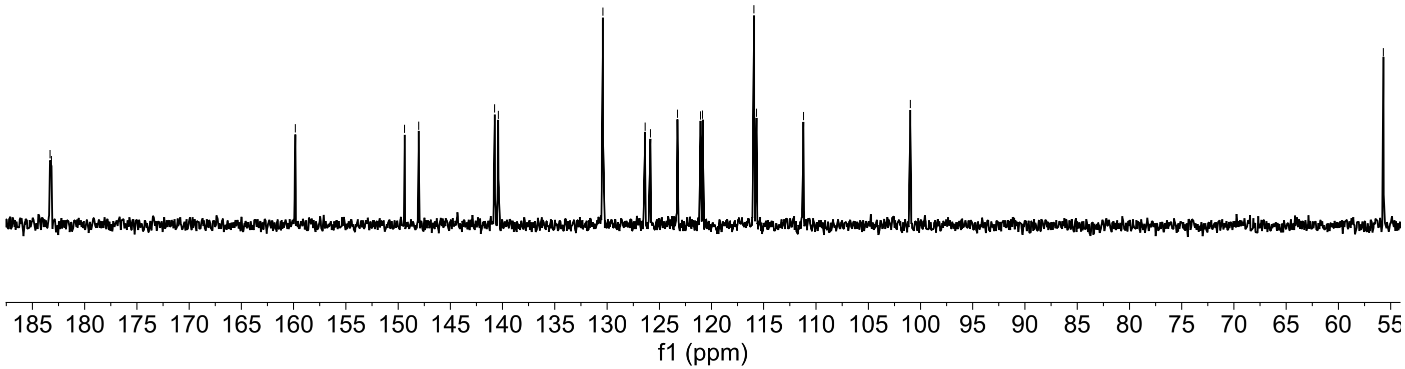

Figure S33. 13C-NMR spectrum of compound 5, DMSO-*d6*, 100 MHz.

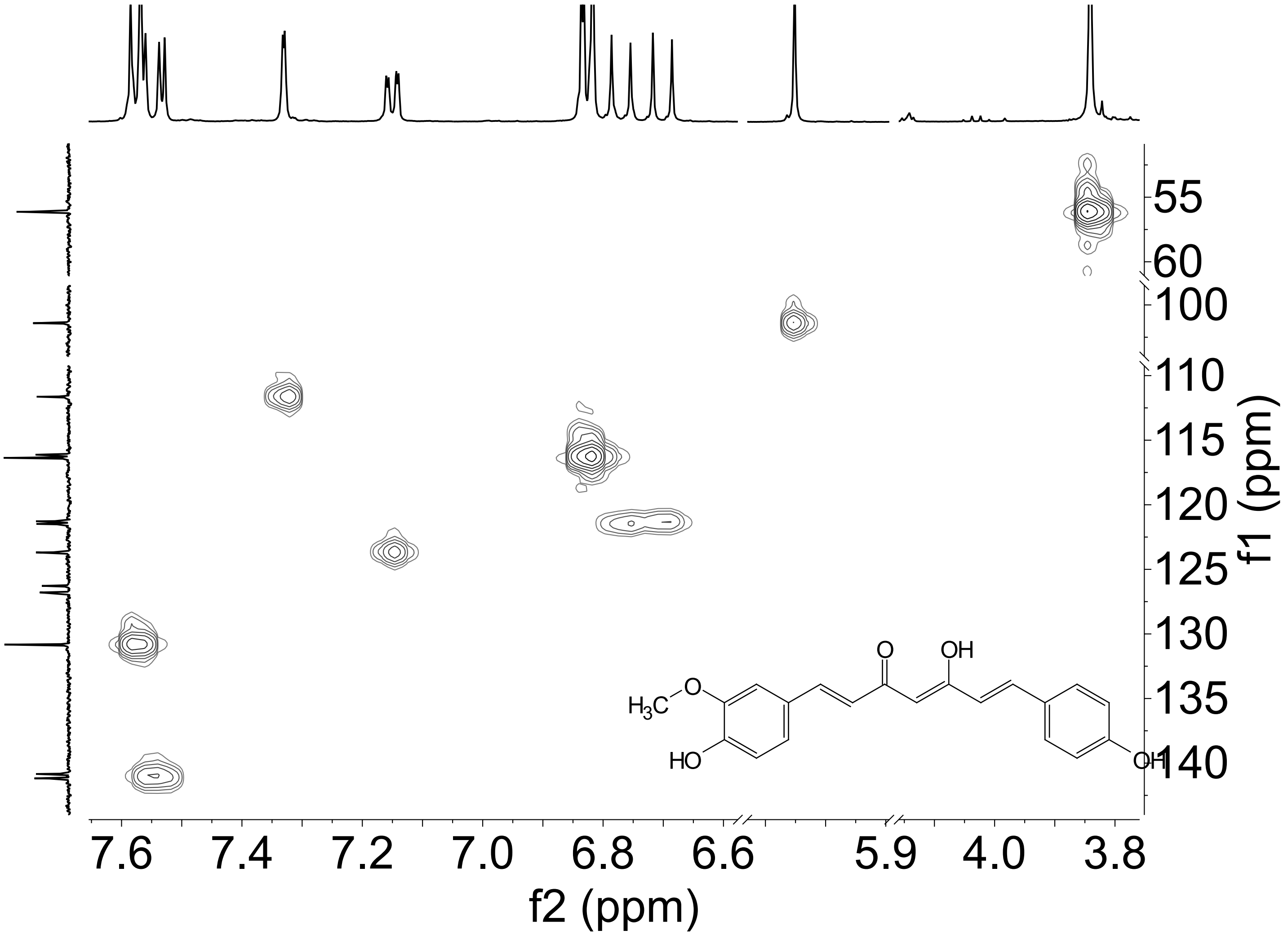

Figure S34. HSQC spectrum of compound 5, DMSO-*d6*, 400MHz.

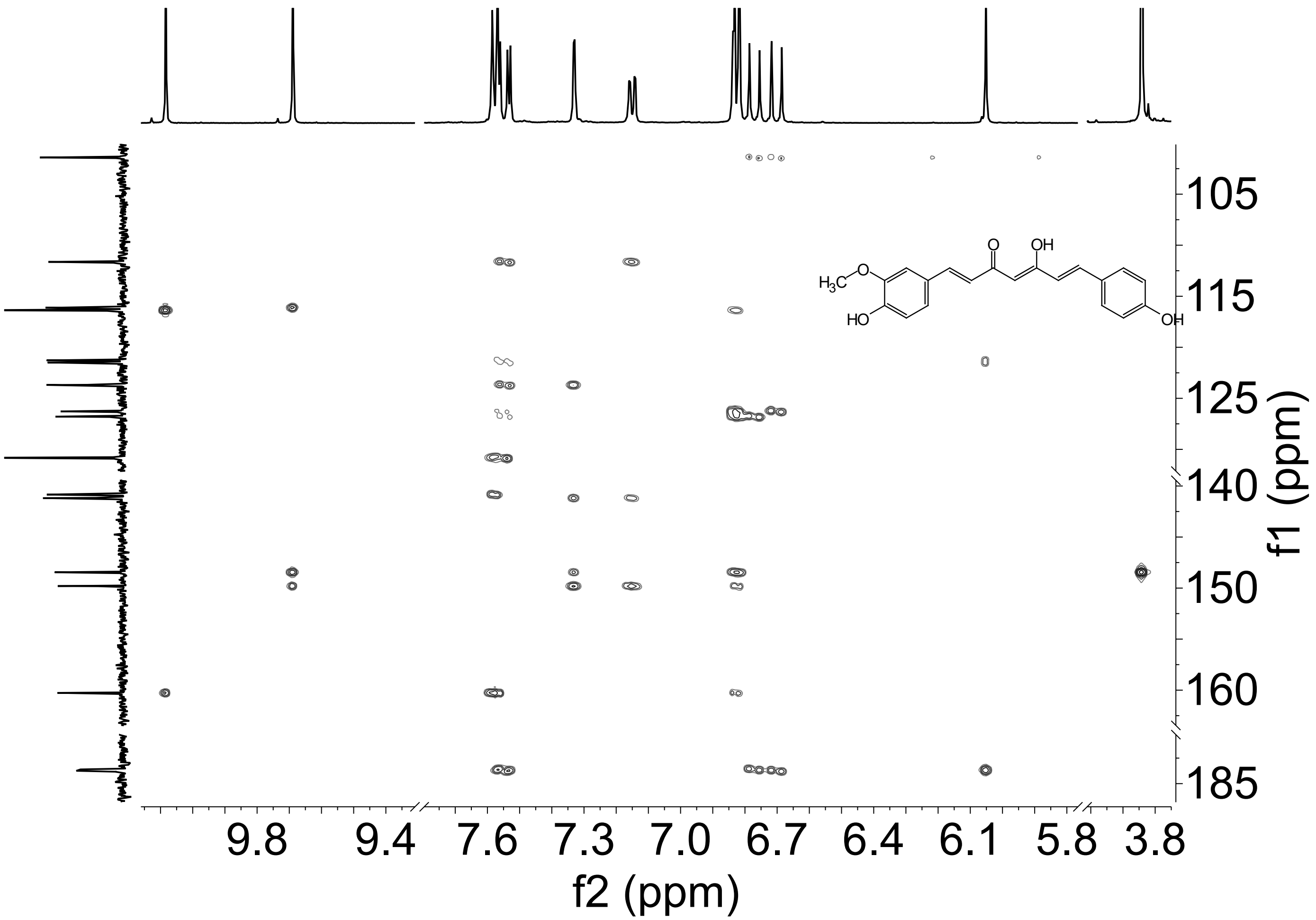

Figure S35. HMBC spectrum of compound 5, DMSO-*d6*, 400MHz.

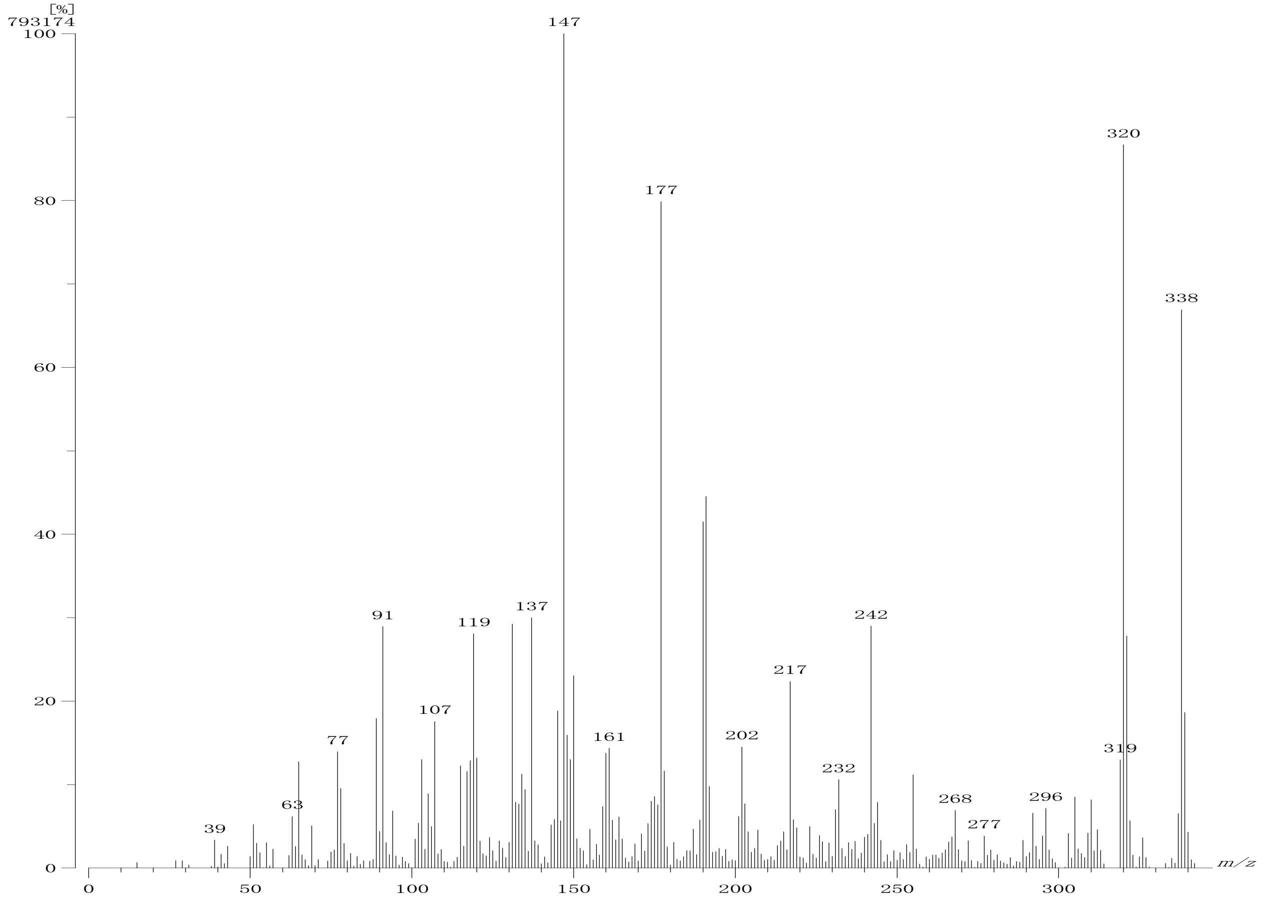

Figure S36. Mass spectrum (EI$^+$) of compound 5.

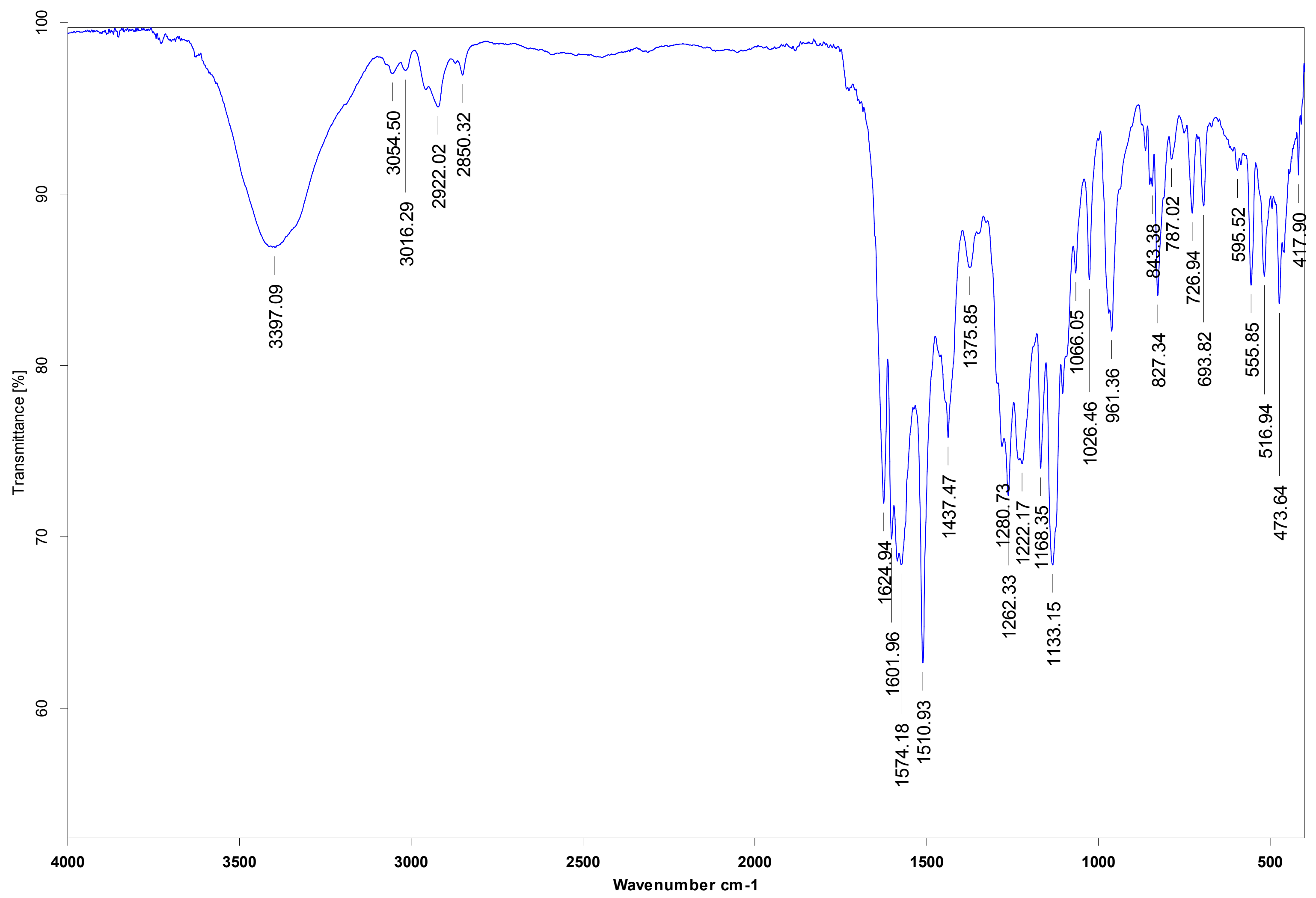

Figure S37. IR spectrum of compound 5.

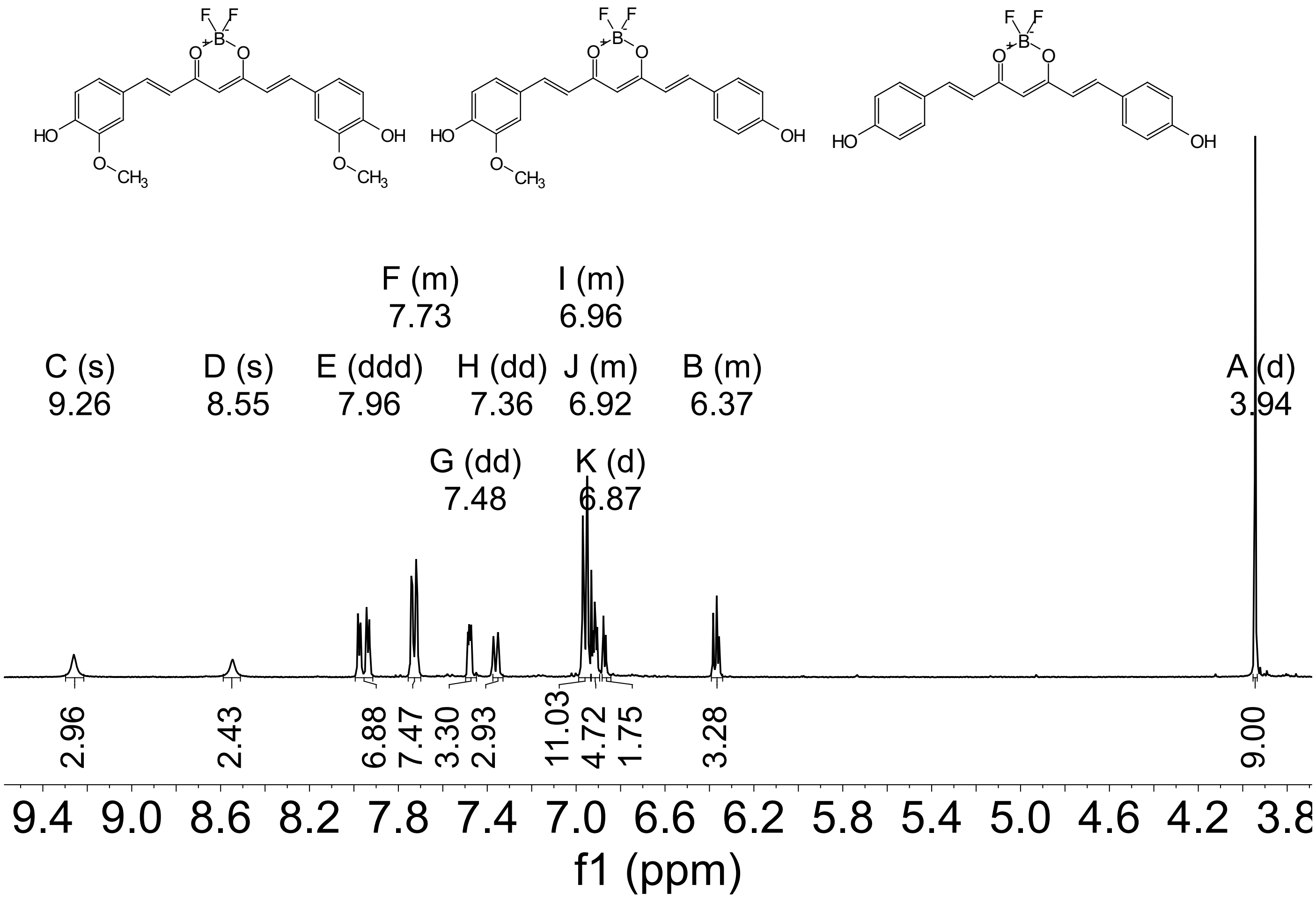

Figure S38. ^{1}**H-NMR spectrum of precursors C-3-BF$_2$ experimental condition i), Acetone-*d6*, 400MHz.**

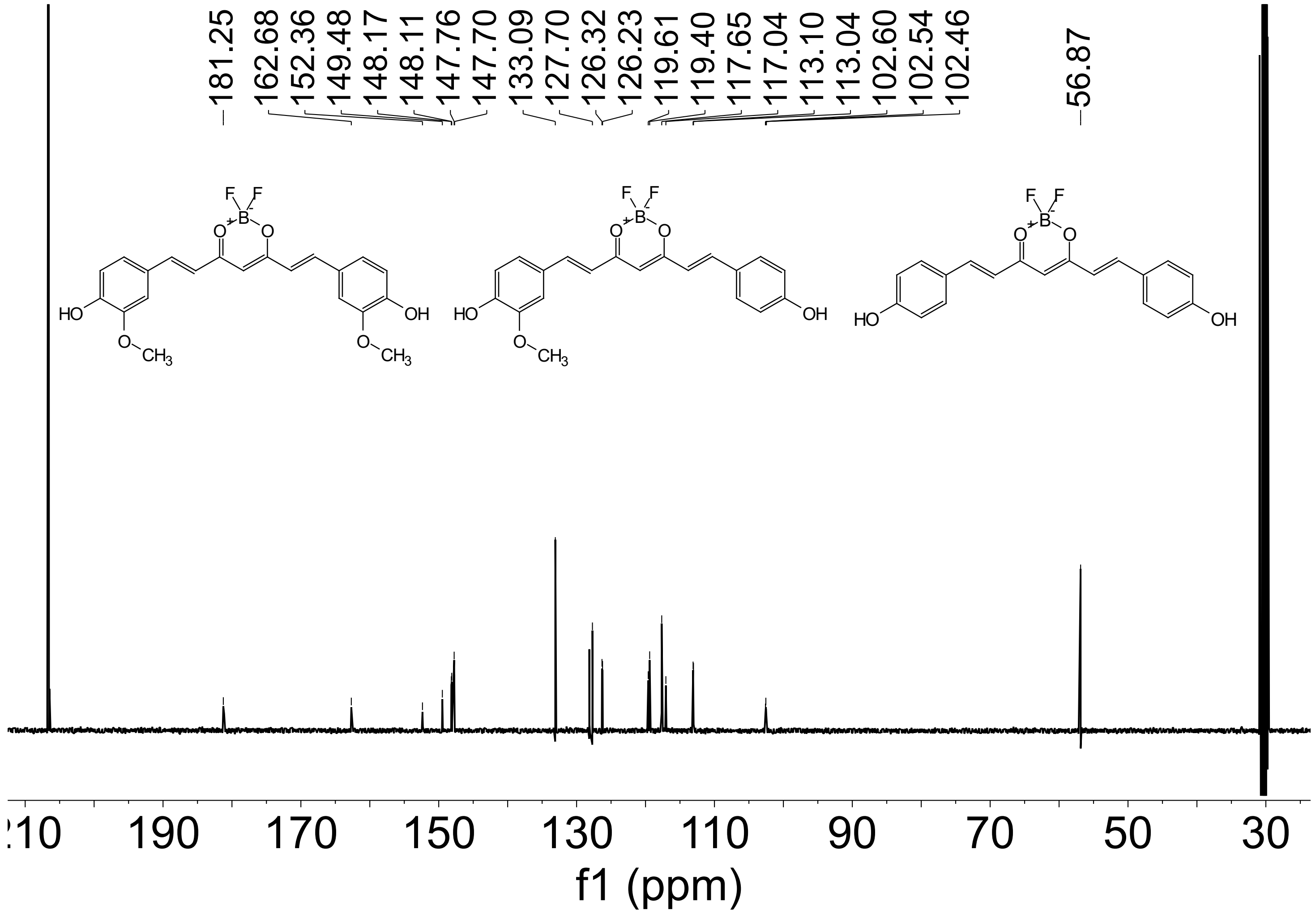

Figure S39. ^{13}C-NMR spectrum of precursors C-3-BF$_2$ experimental condition i), Acetone-*d6*, 100MHz.

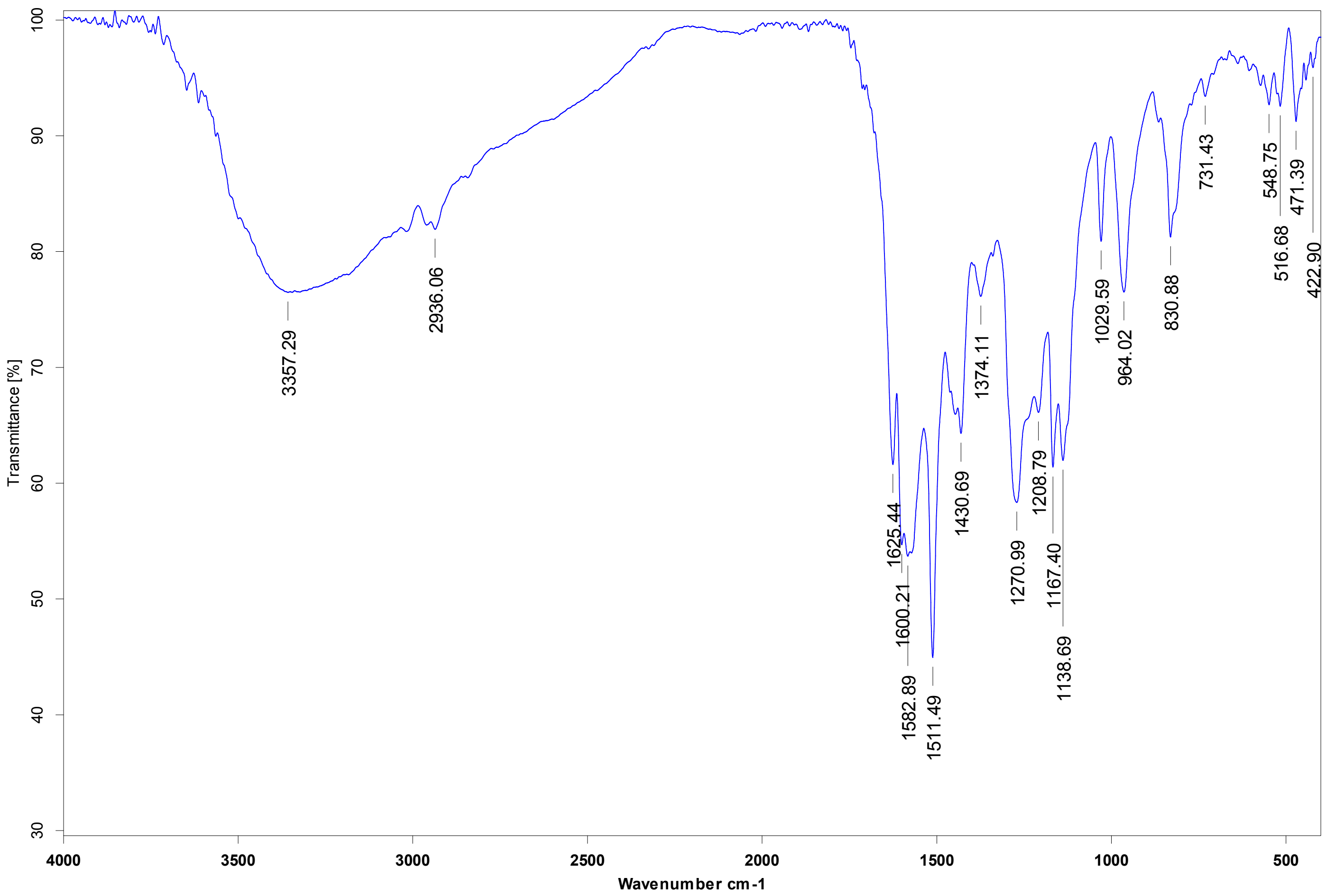

Figure S40. IR spectrum of precursors C-3-BF_2 experimental condition i).

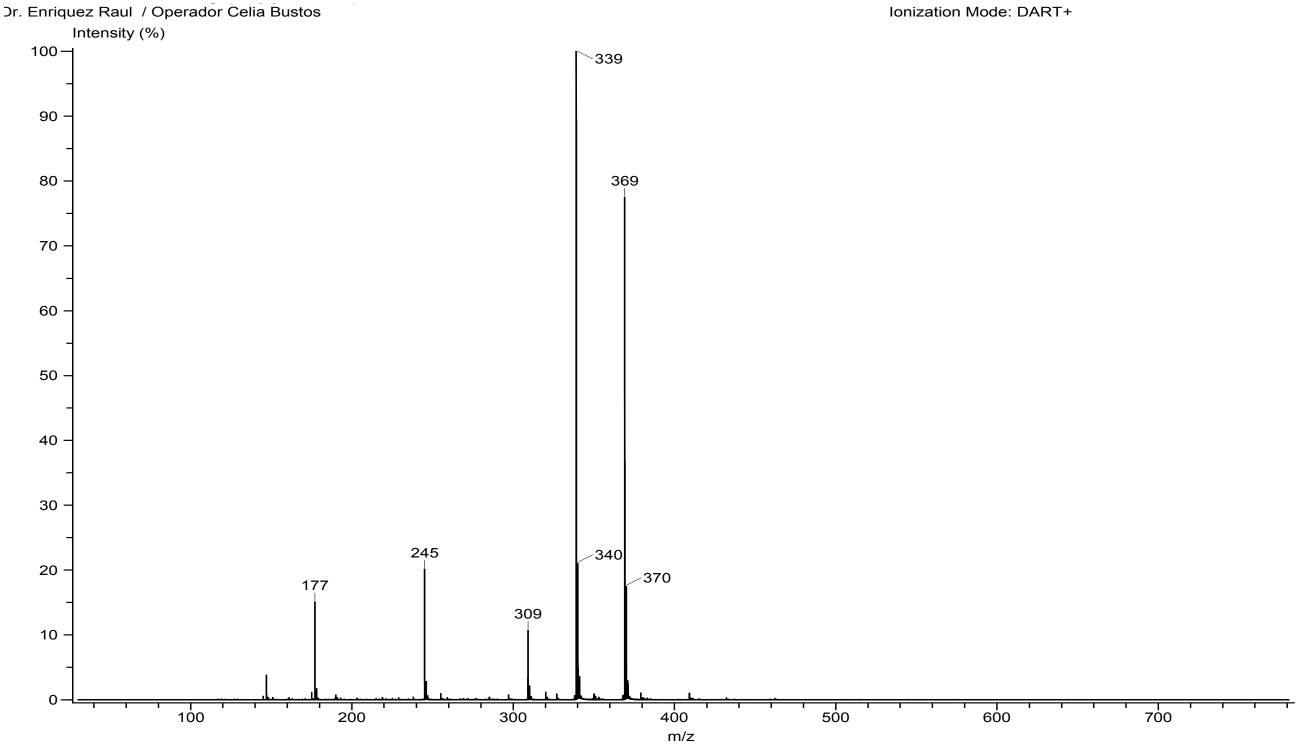

Figure S41. Mass spectrum (DART$^+$) of precursors C-3-BF_2 experimental condition i).

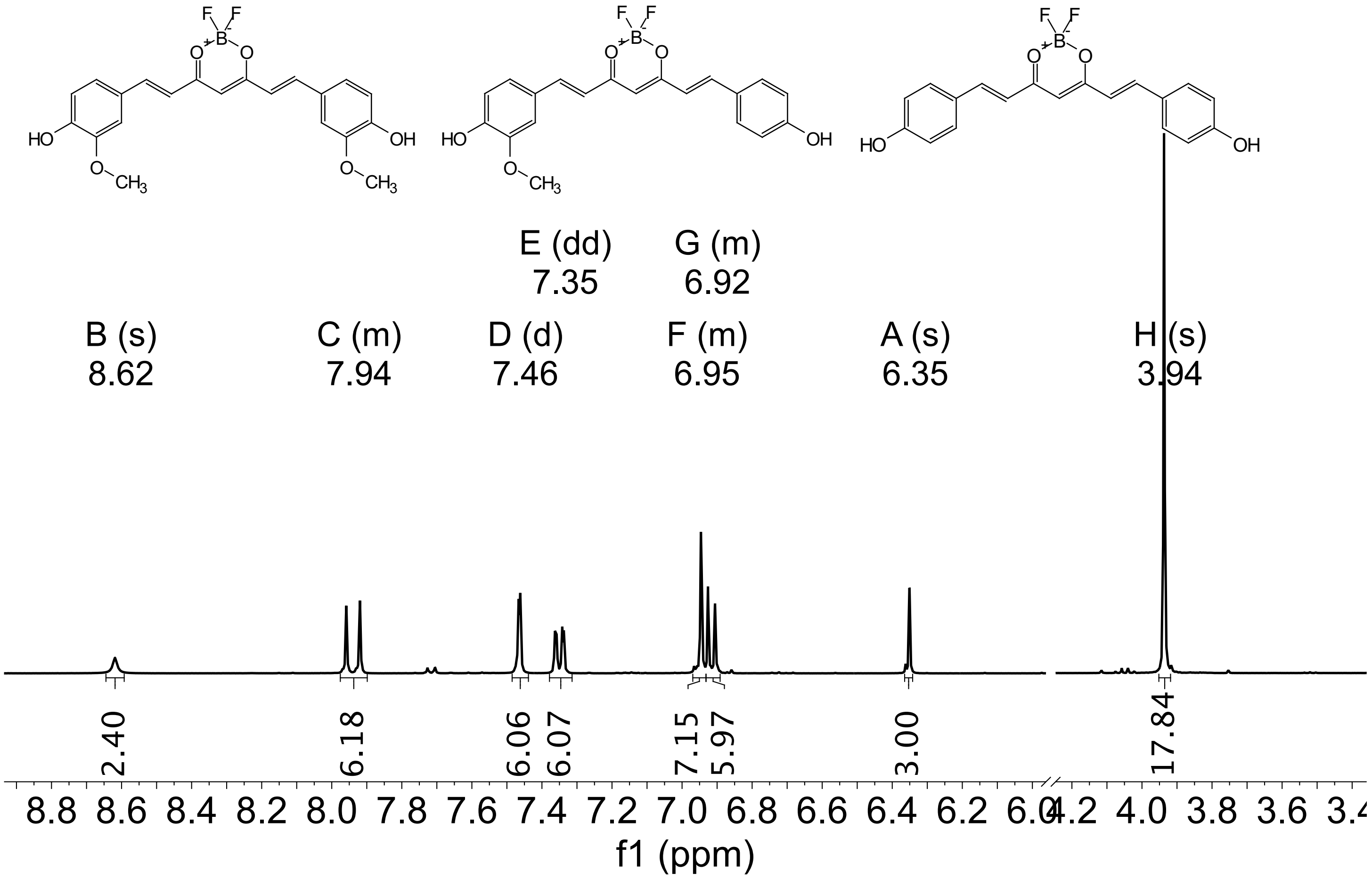

Figure S42. ^{1}H-NMR spectrum of precursors C-3-BF_2 experimental condition ii), Acetone-*d6*, 400MHz.

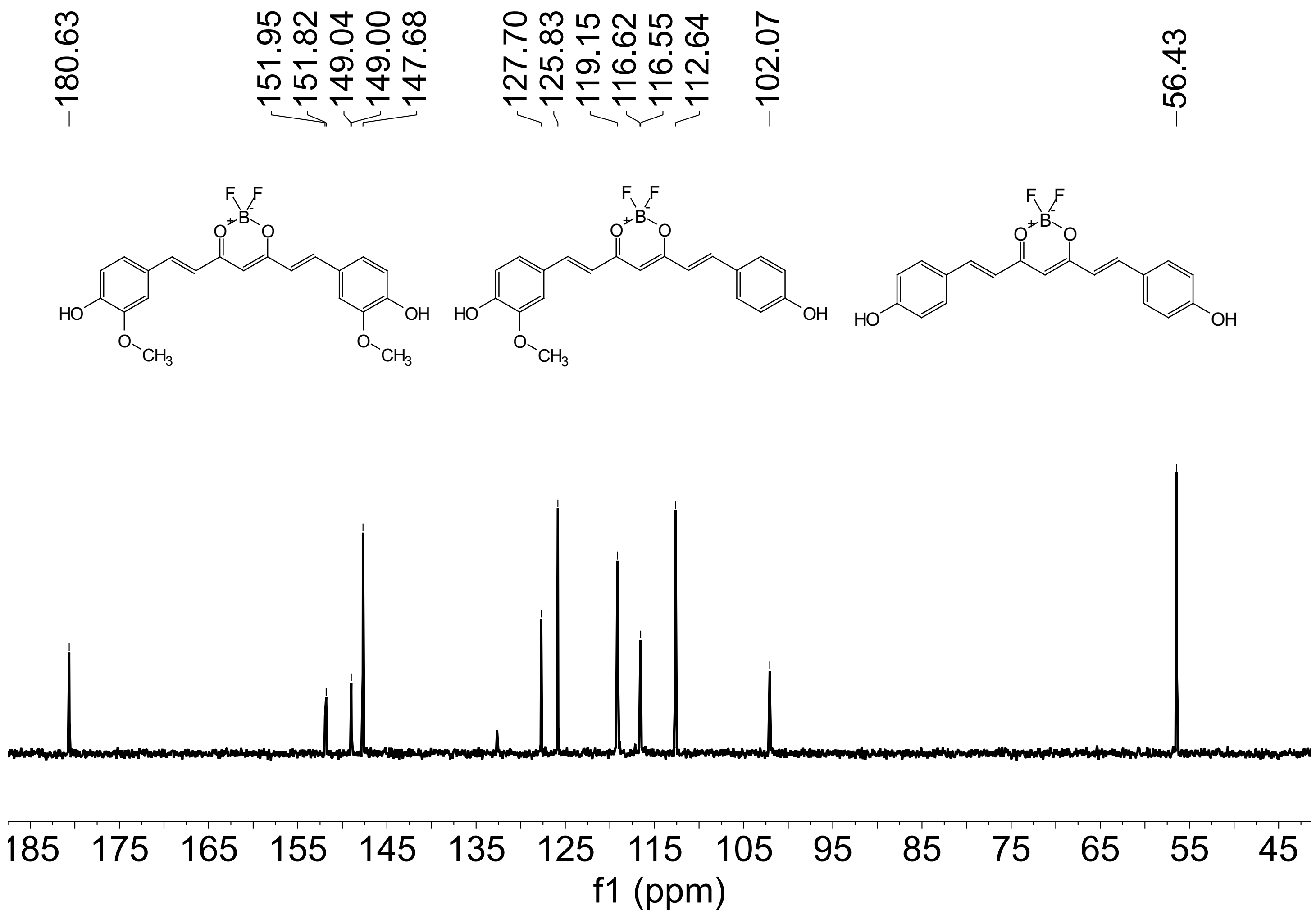

Figure S43. ^{13}C-NMR spectrum of precursors C-3-BF_2 experimental condition ii), Acetone-*d6*, 100MHz.

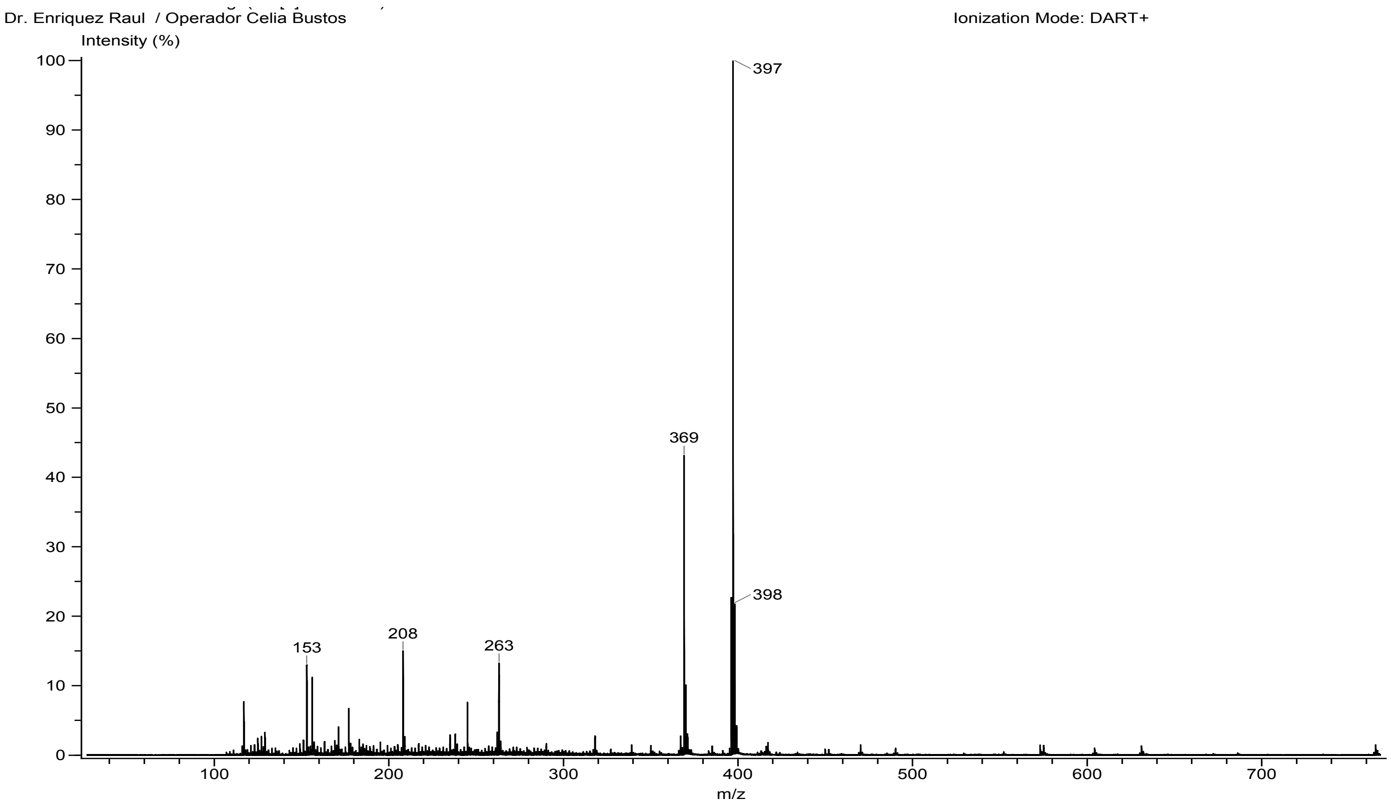

Figure S44. Mass spectrum (DART$^+$) of precursors C-3-BF_2 experimental condition ii).

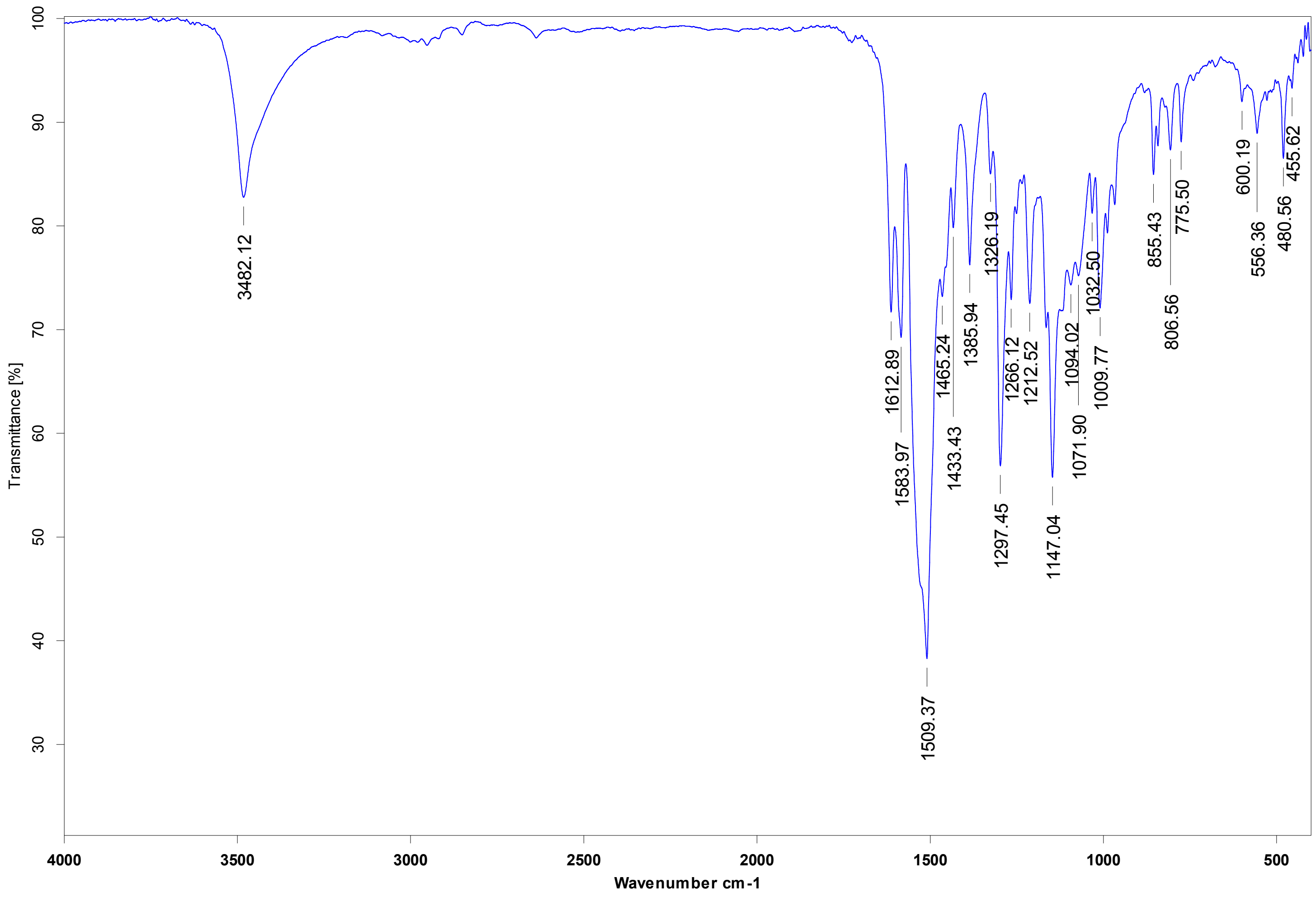

Figure S45. IR spectrum of precursors C-3-BF_2 experimental condition ii).

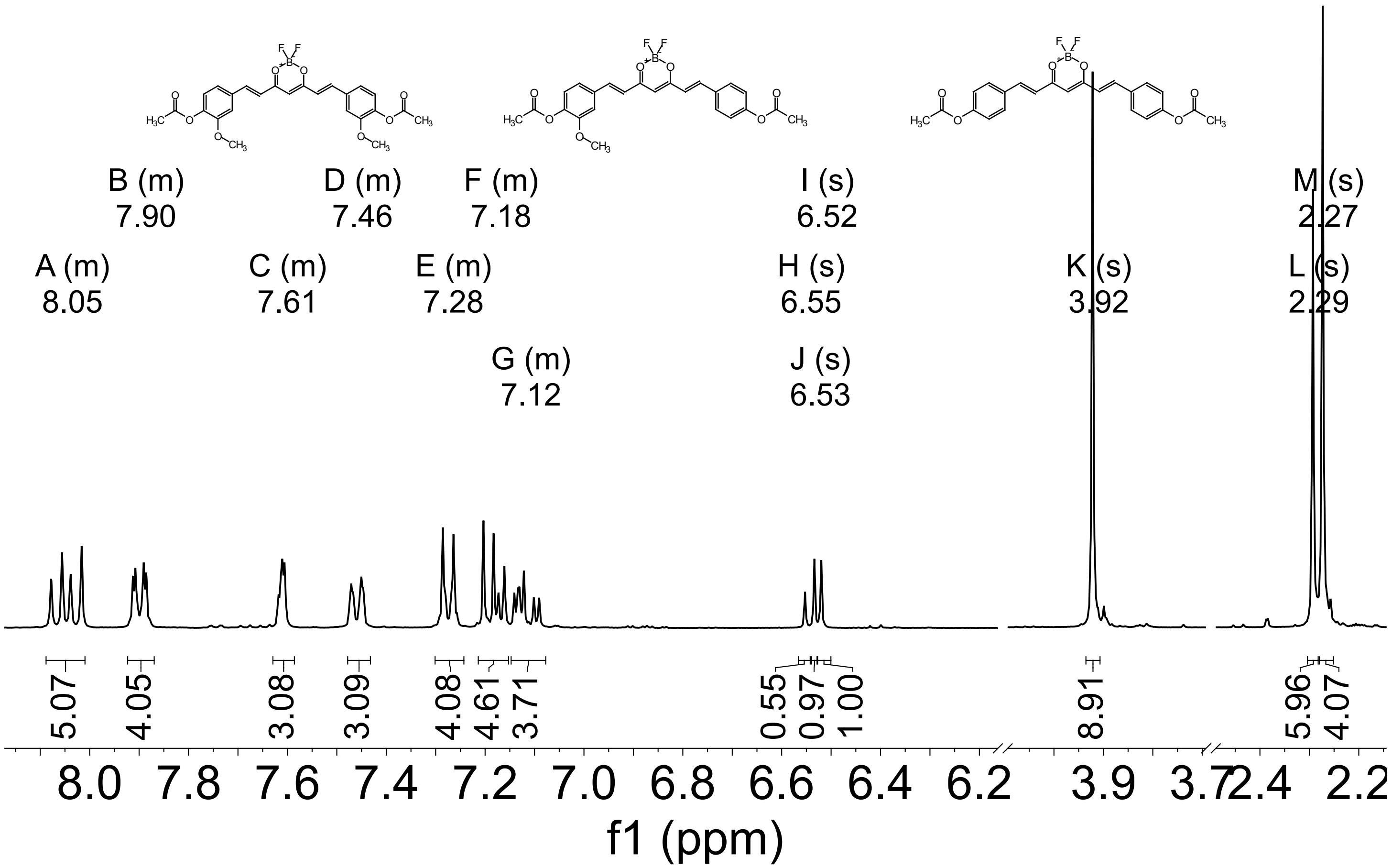

Figure S46. ^{1}H-NMR spectrum of precursors C-3-BF_2 experimental condition iii), Acetone-*d6*, 400MHz.

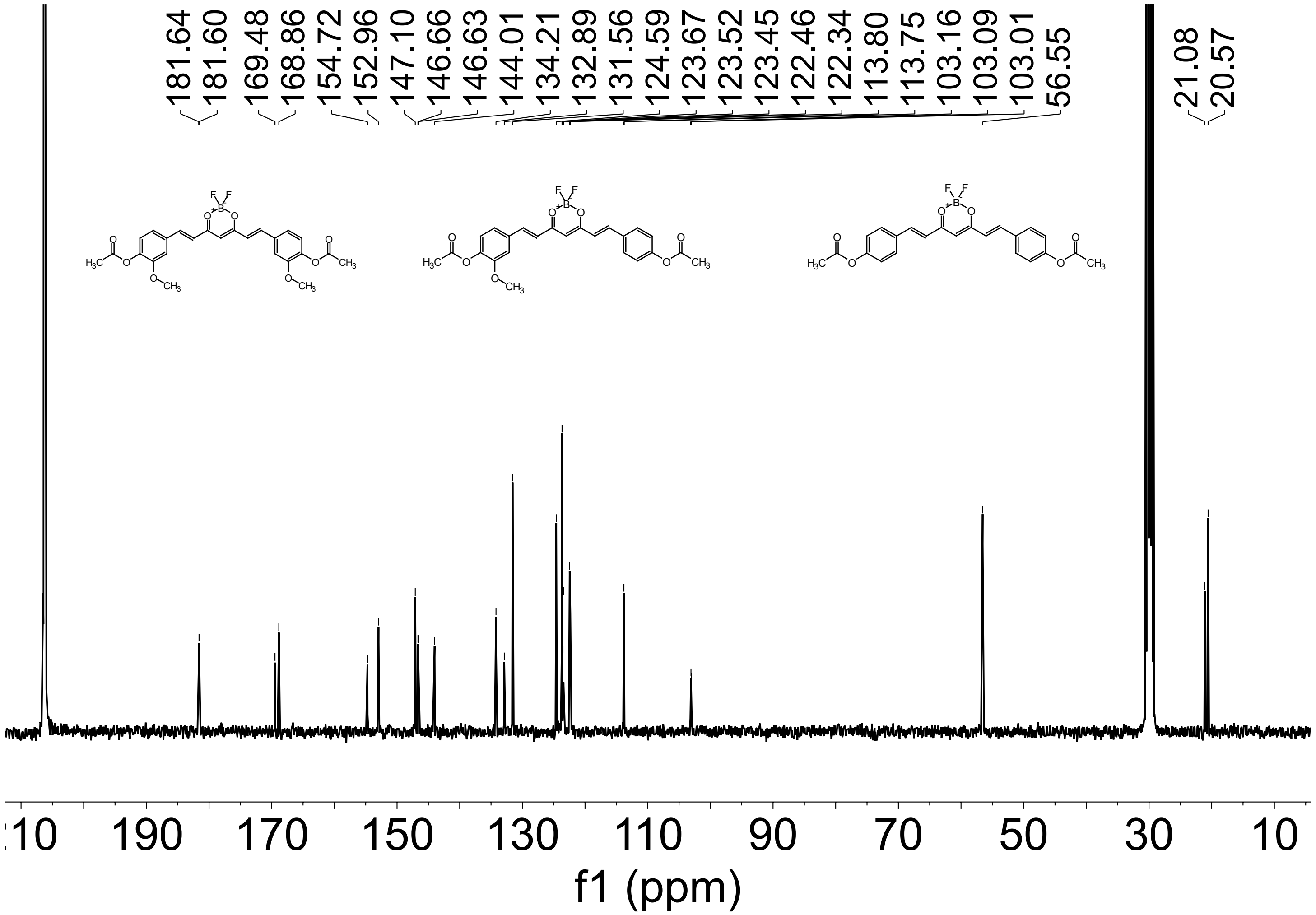

Figure S47. ^{13}C-NMR spectrum of precursors C-3-BF_2 experimental condition iii), Acetone-*d6*, 100MHz.

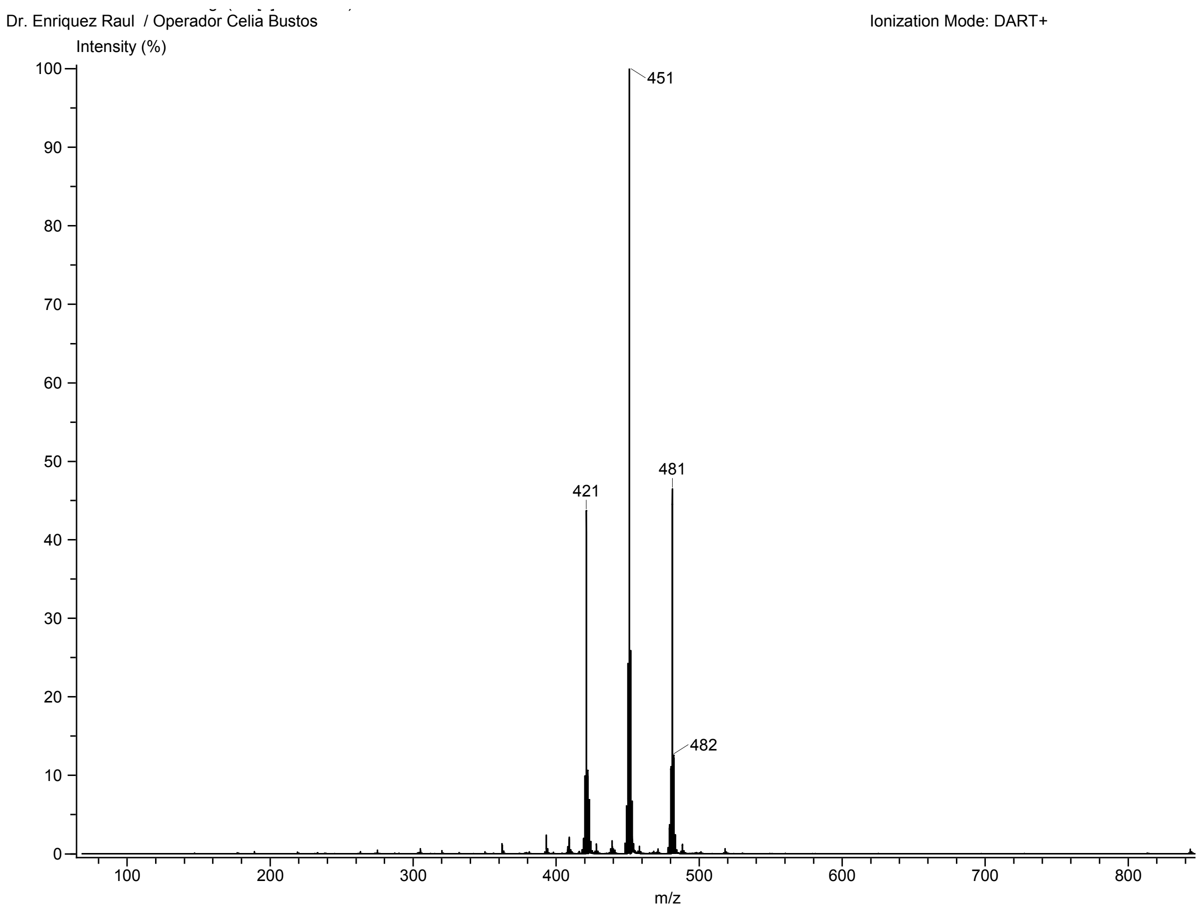

Figure S48. Mass spectrum (DART^{+}) of precursors C-3-BF_2 experimental condition iii).

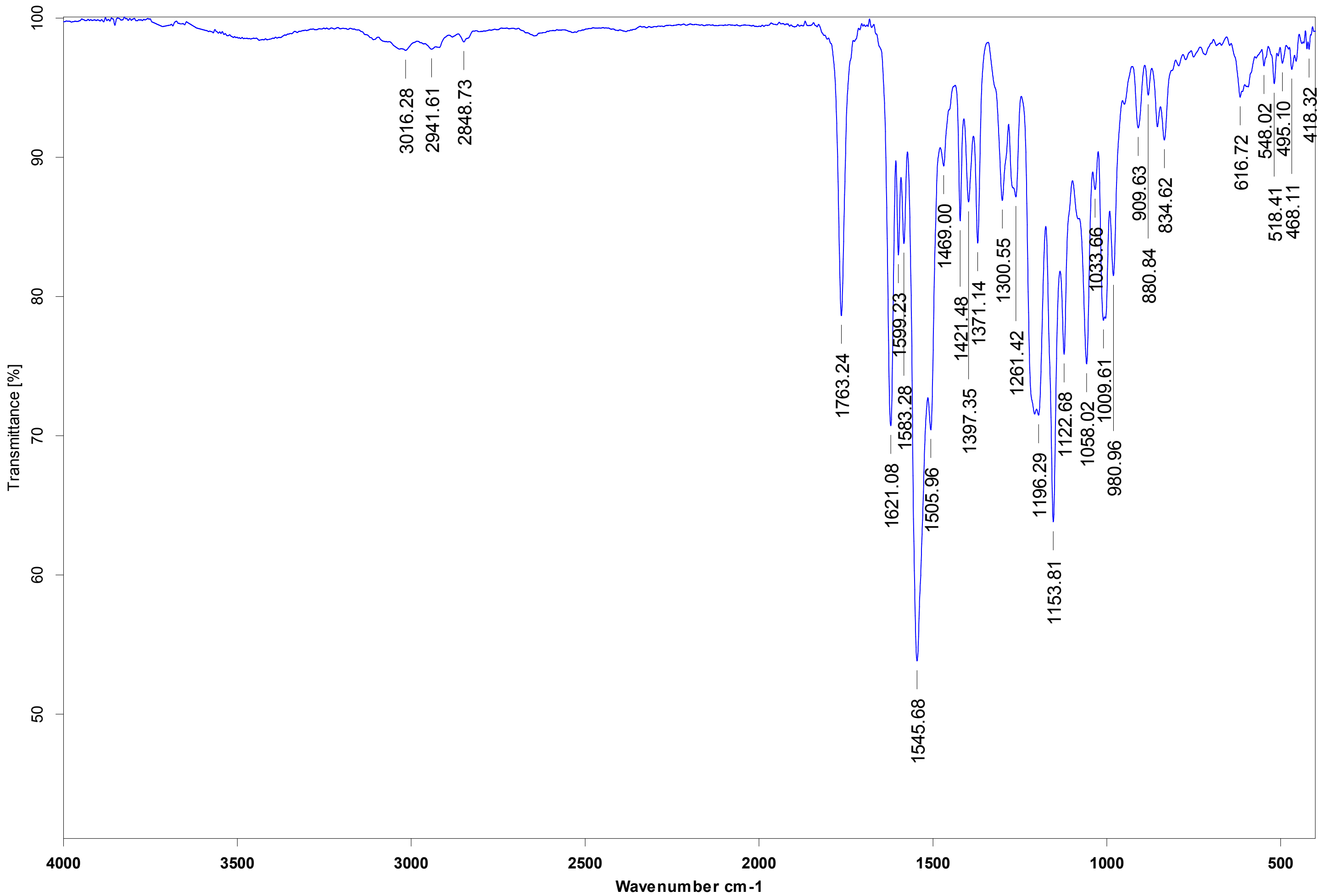

Figure S49. IR spectrum of precursors C-3-BF_2 experimental condition iii).

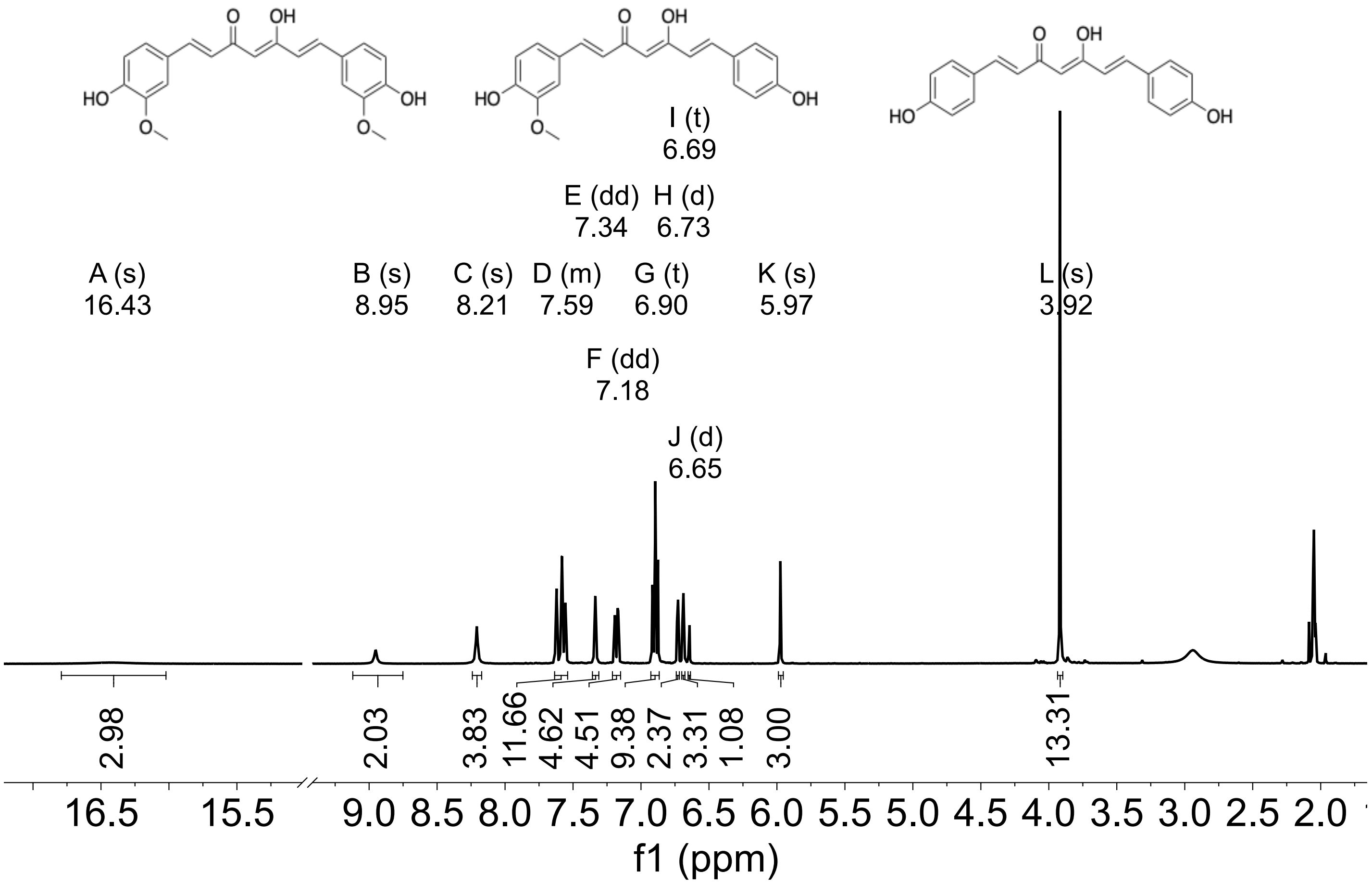

Figure S50. ^{1}**H-NMR spectrum of curcuminoids C-3 (CUR, DMC, BDMC) from experimental condition i), Acetone-*d6*, 400MHz.**

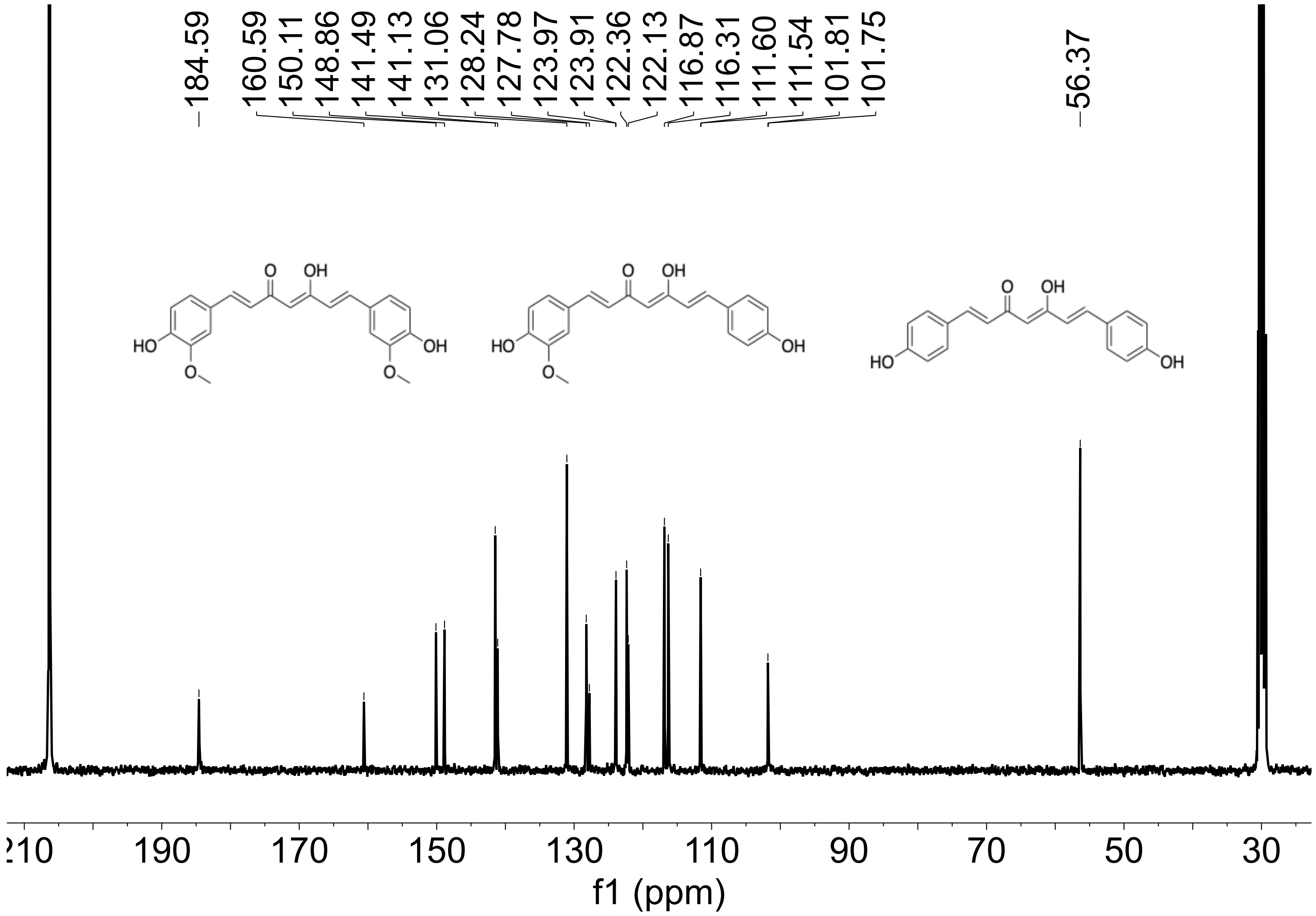

Figure S51. ^{13}C-NMR spectrum of curcuminoids C-3 (CUR, DMC, BDMC) from experimental condition i), Acetone-*d6*, 100MHz.

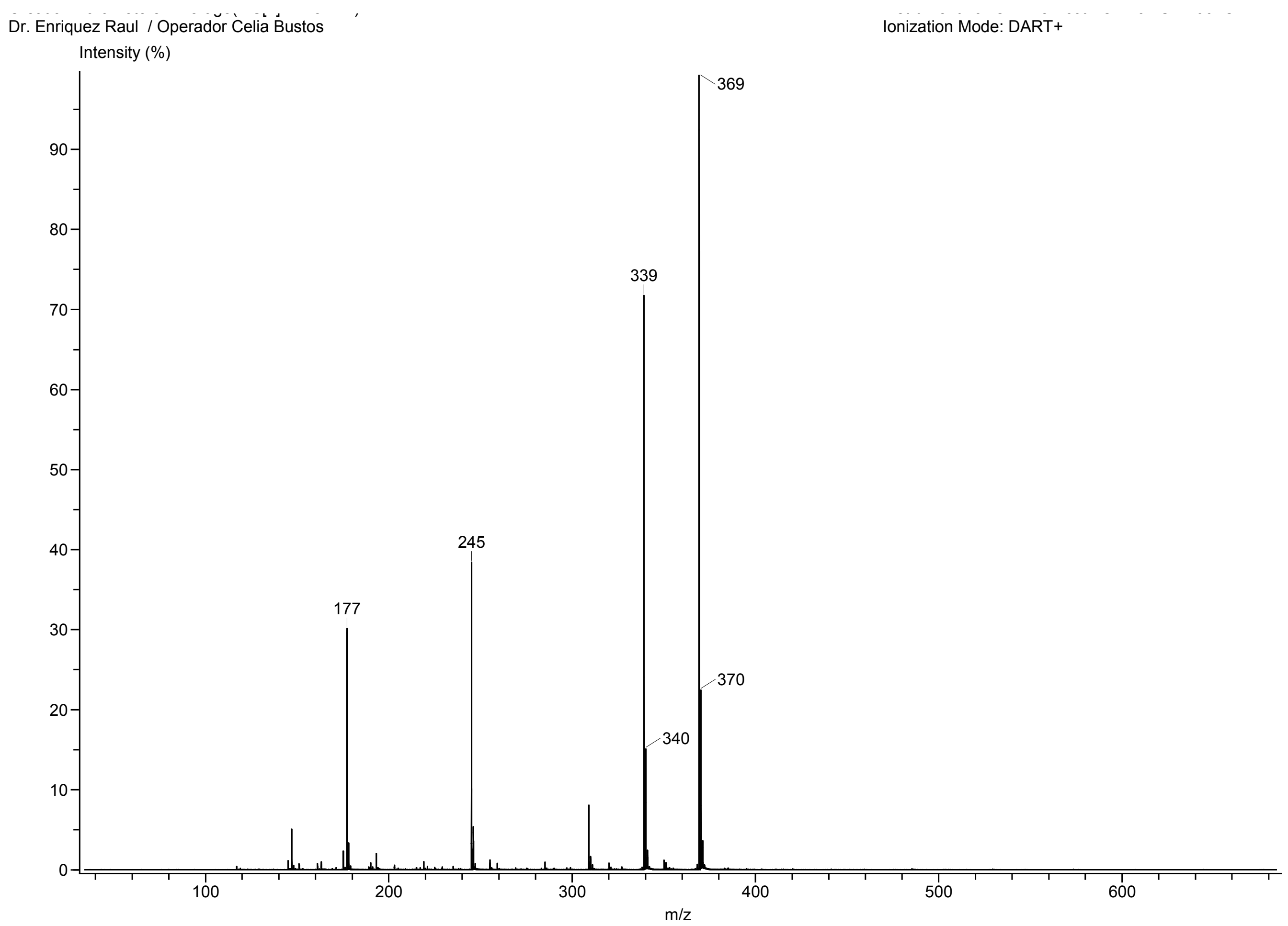

Figure S52. Mass spectrum of curcuminoids C-3 (DART$^+$) from experimental condition i).

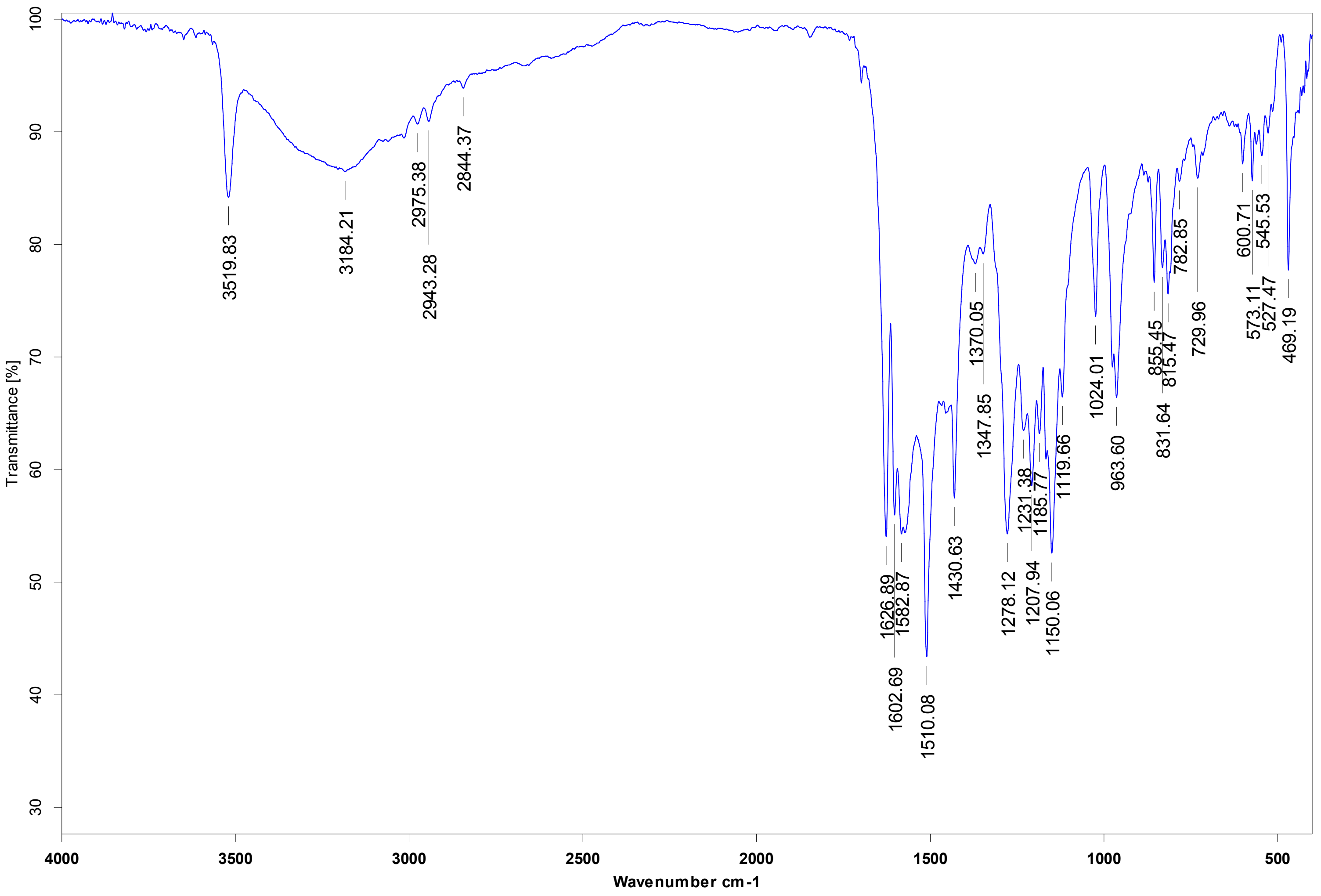

Figure S53. IR spectrum of curcuminoids C-3 from experimental condition i).

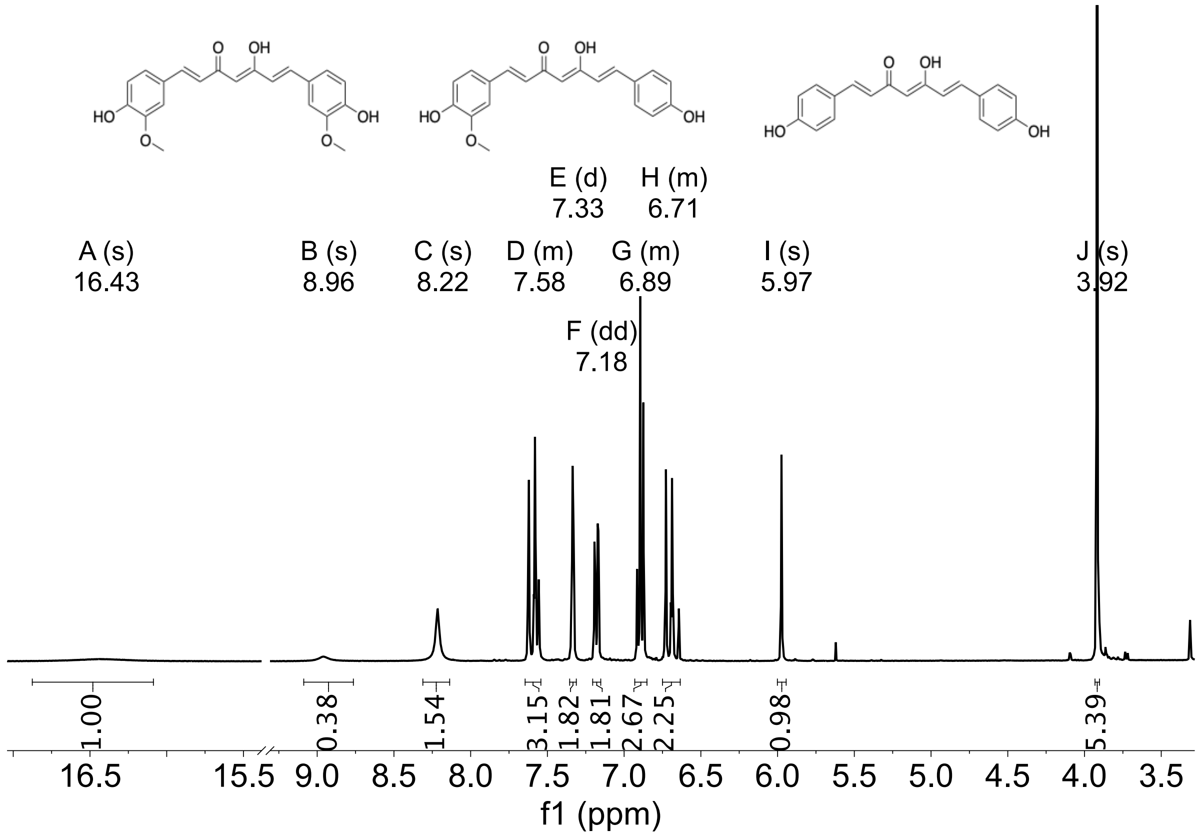

Figure S54. ^{1}H-NMR spectrum of curcuminoids C-3 (CUR, DMC, BDMC) from experimental condition ii), Acetone-*d6*, 400MHz.

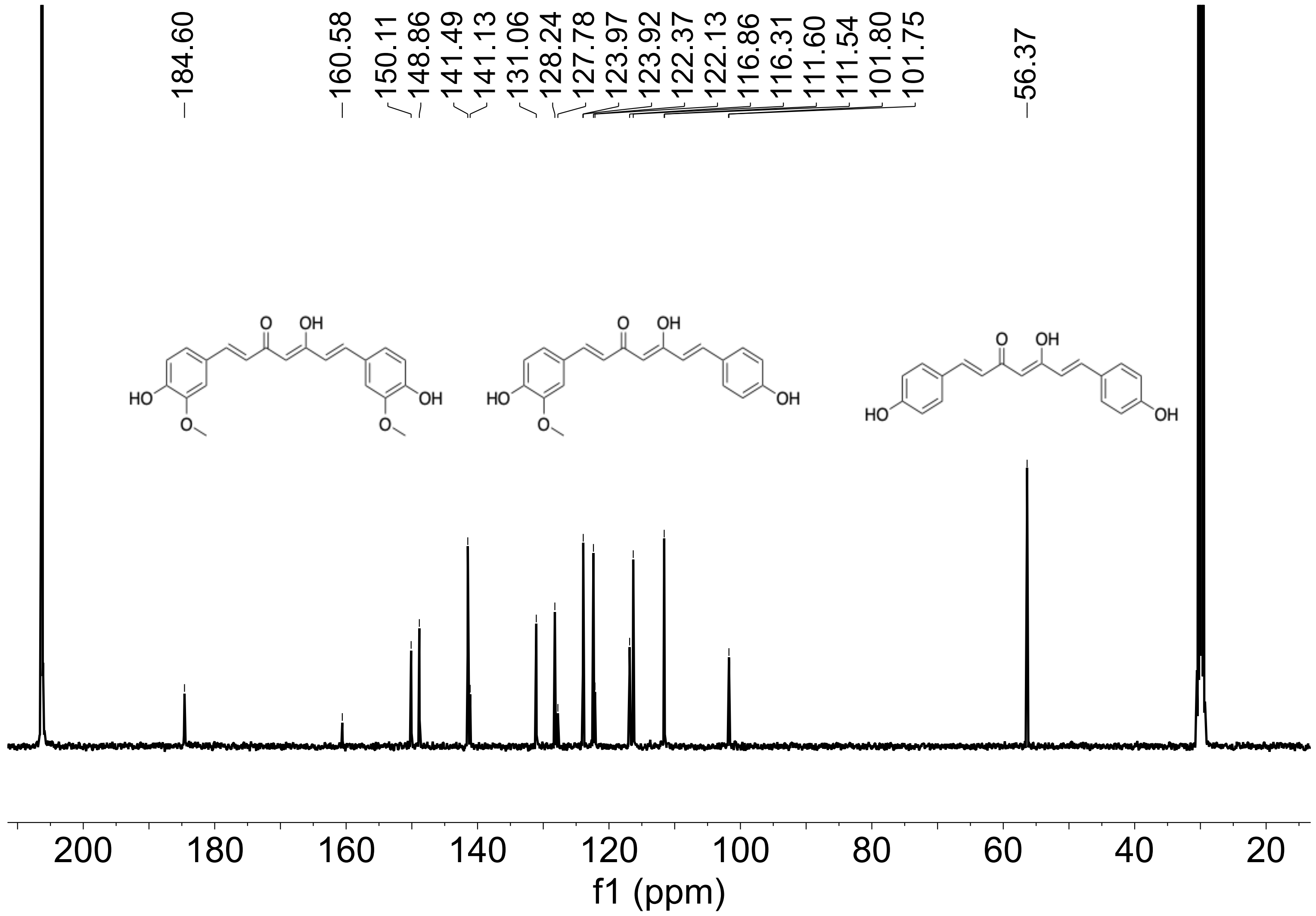

Figure S55. ^{13}C-NMR spectrum of curcuminoids C-3 (CUR, DMC, BDMC) from experimental condition ii), Acetone-*d6*, 100MHz.

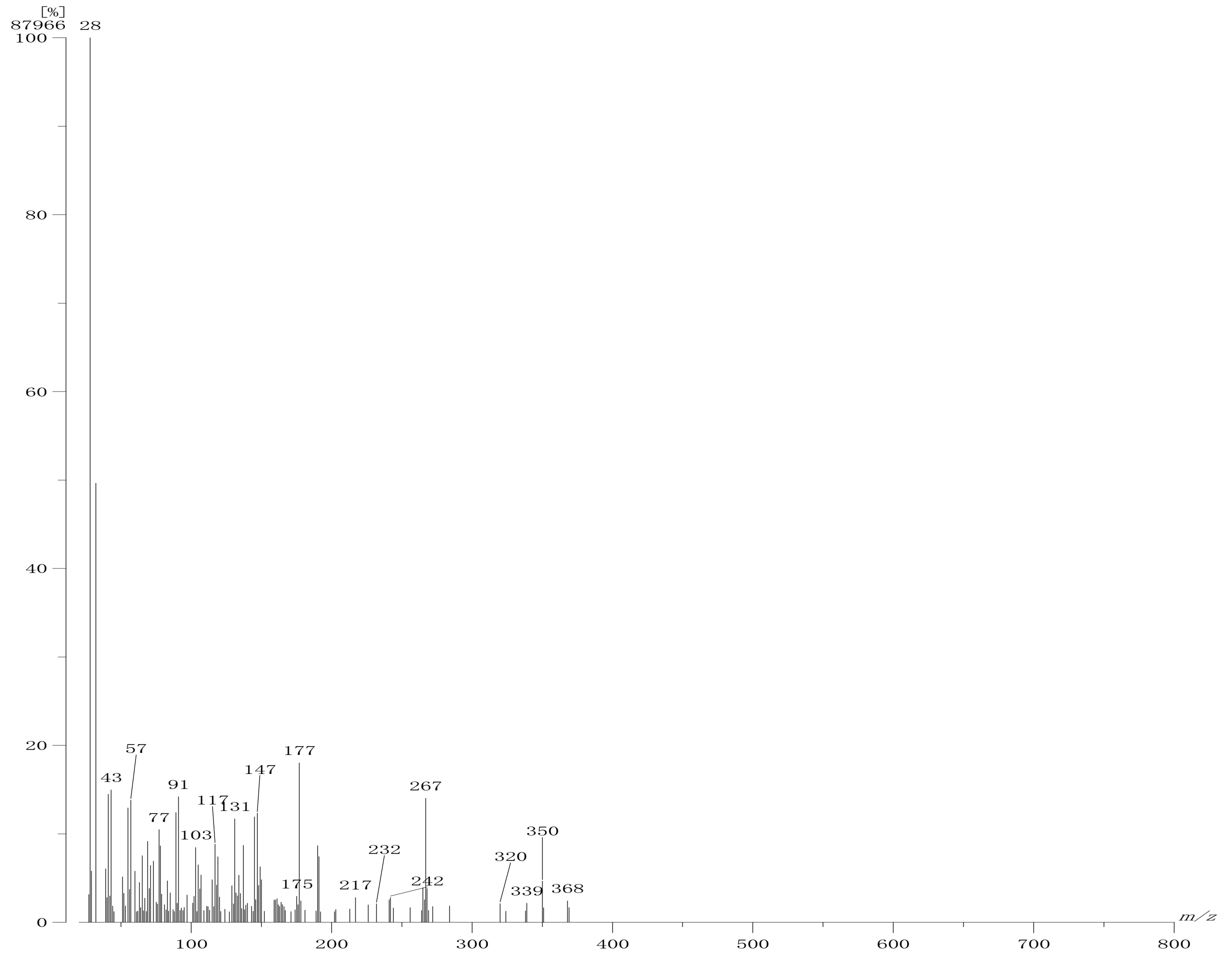

Figure S56. Mass spectrum of curcuminoids C-3 (EI$^+$) from experimental condition ii).

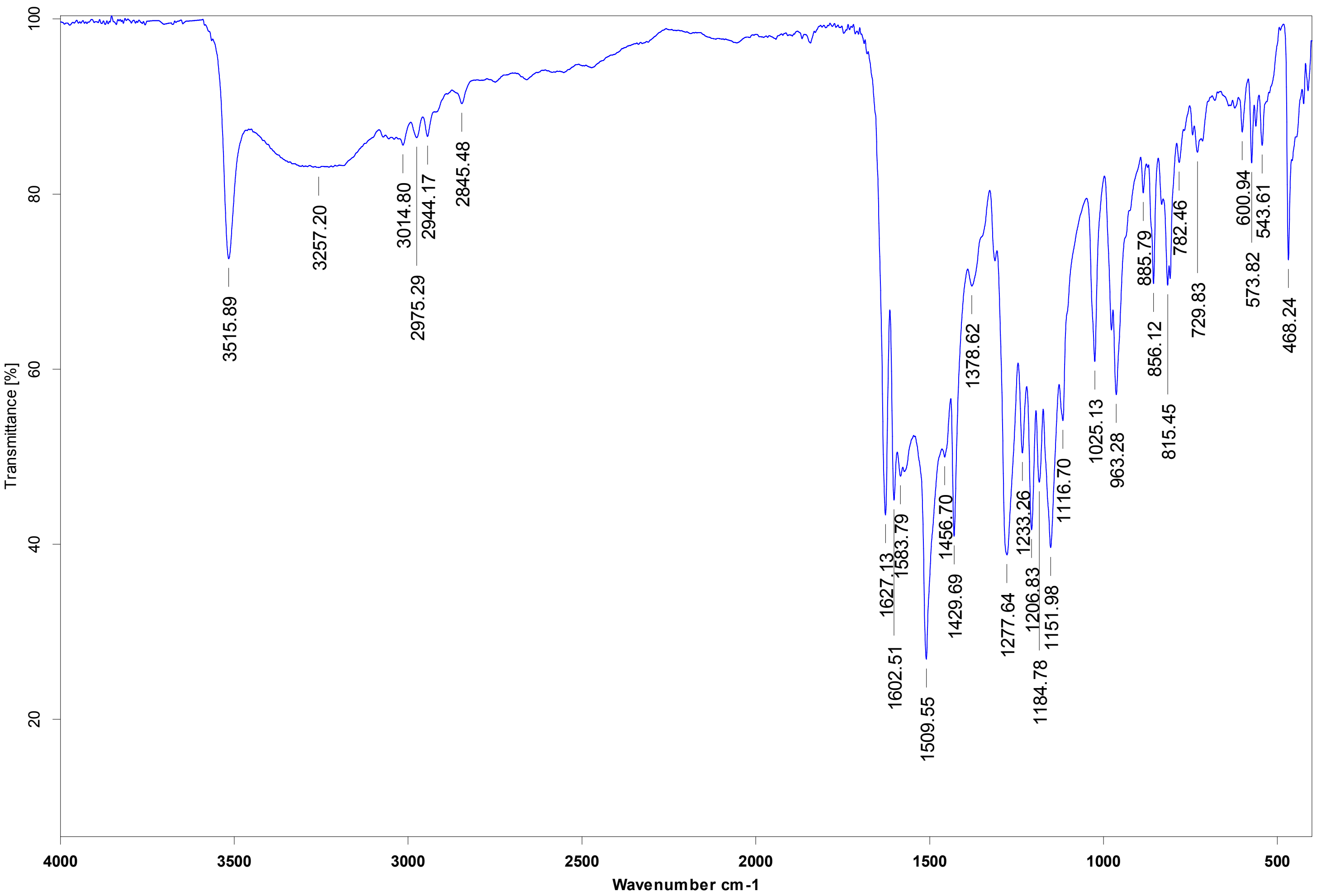

Figure S57. IR spectrum of curcuminoids C-3 from experimental condition ii).

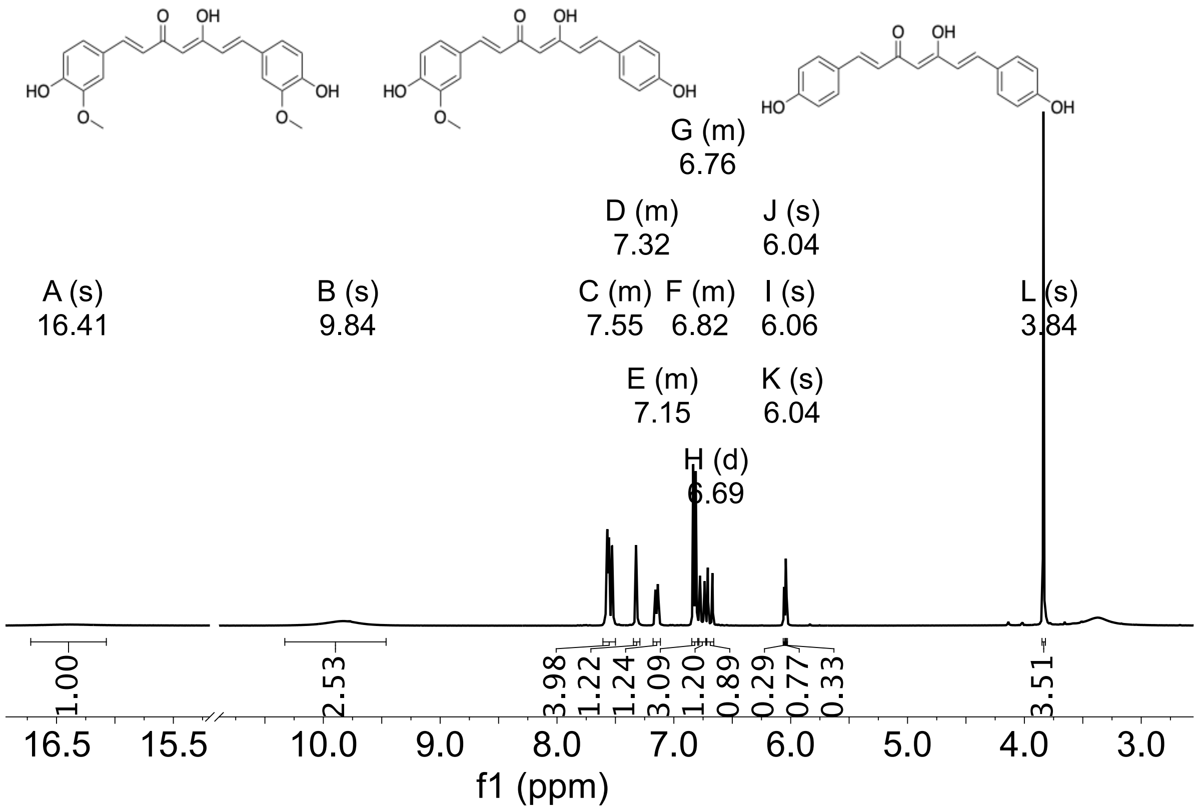

Figure S58. **^{1}H-NMR spectrum of curcuminoids C-3 (CUR, DMC, BDMC) from experimental condition iii), Acetone-*d6*, 400MHz.**

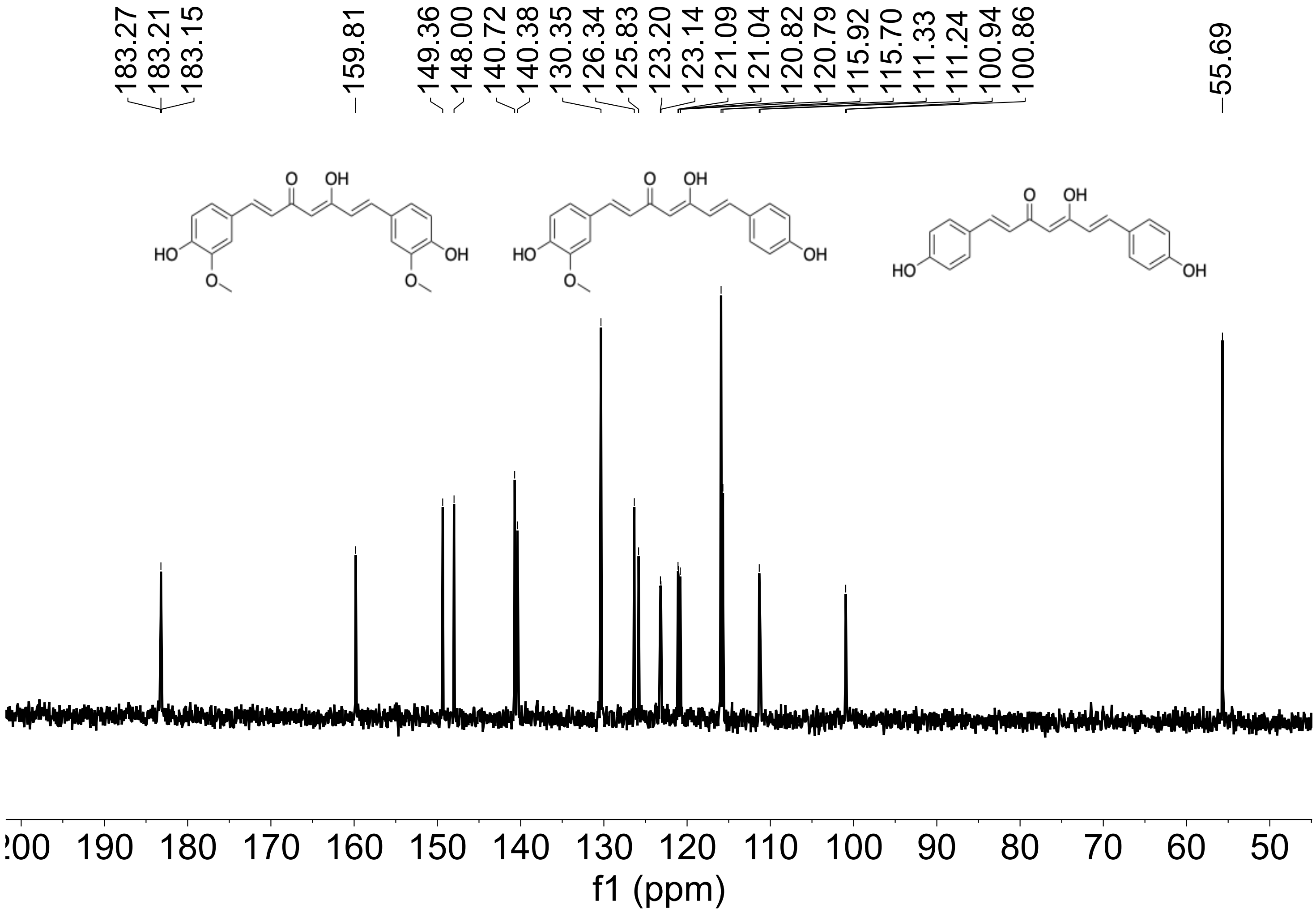

Figure S59. ^{13}**C-NMR spectrum of curcuminoids C-3 (CUR, DMC, BDMC) from experimental condition iii), Acetone-*d6*, 100MHz.**

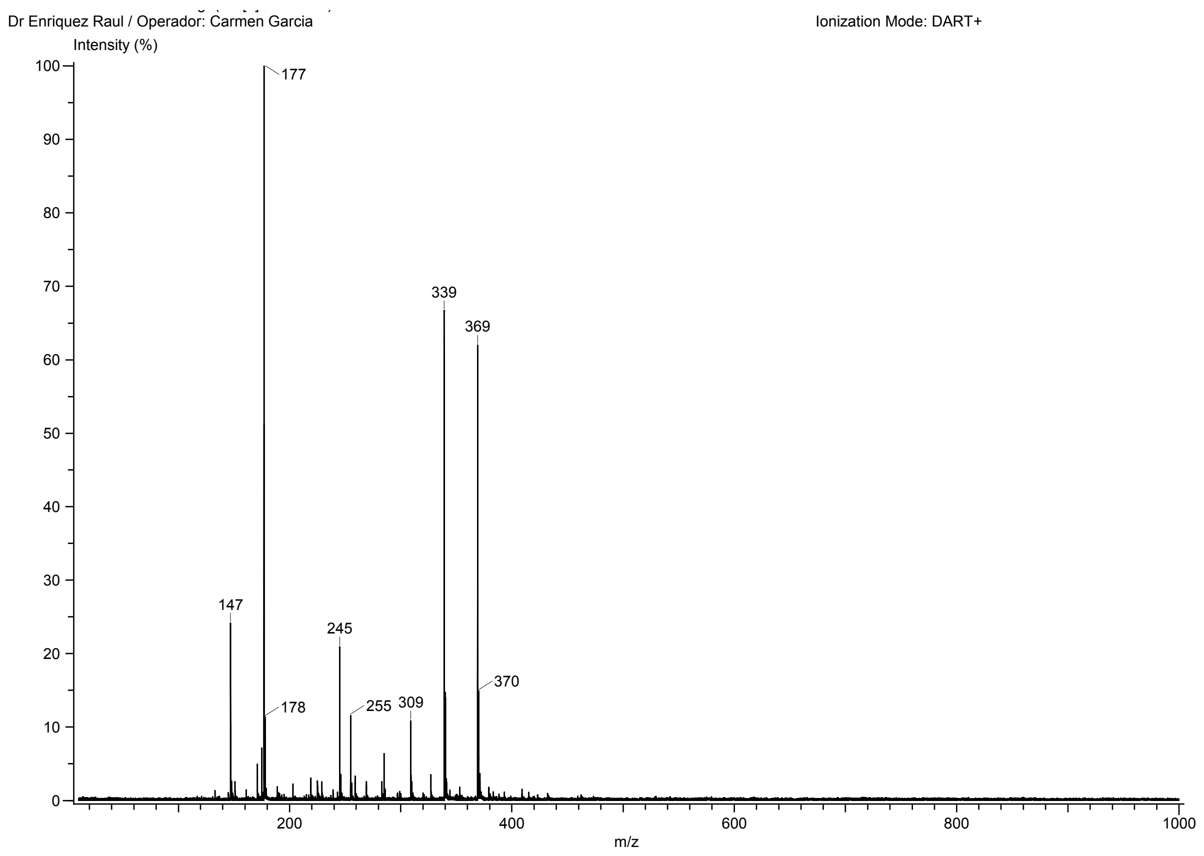

Figure S60. Mass spectrum (DART+) of curcuminoids C3 from experimental condition iii).

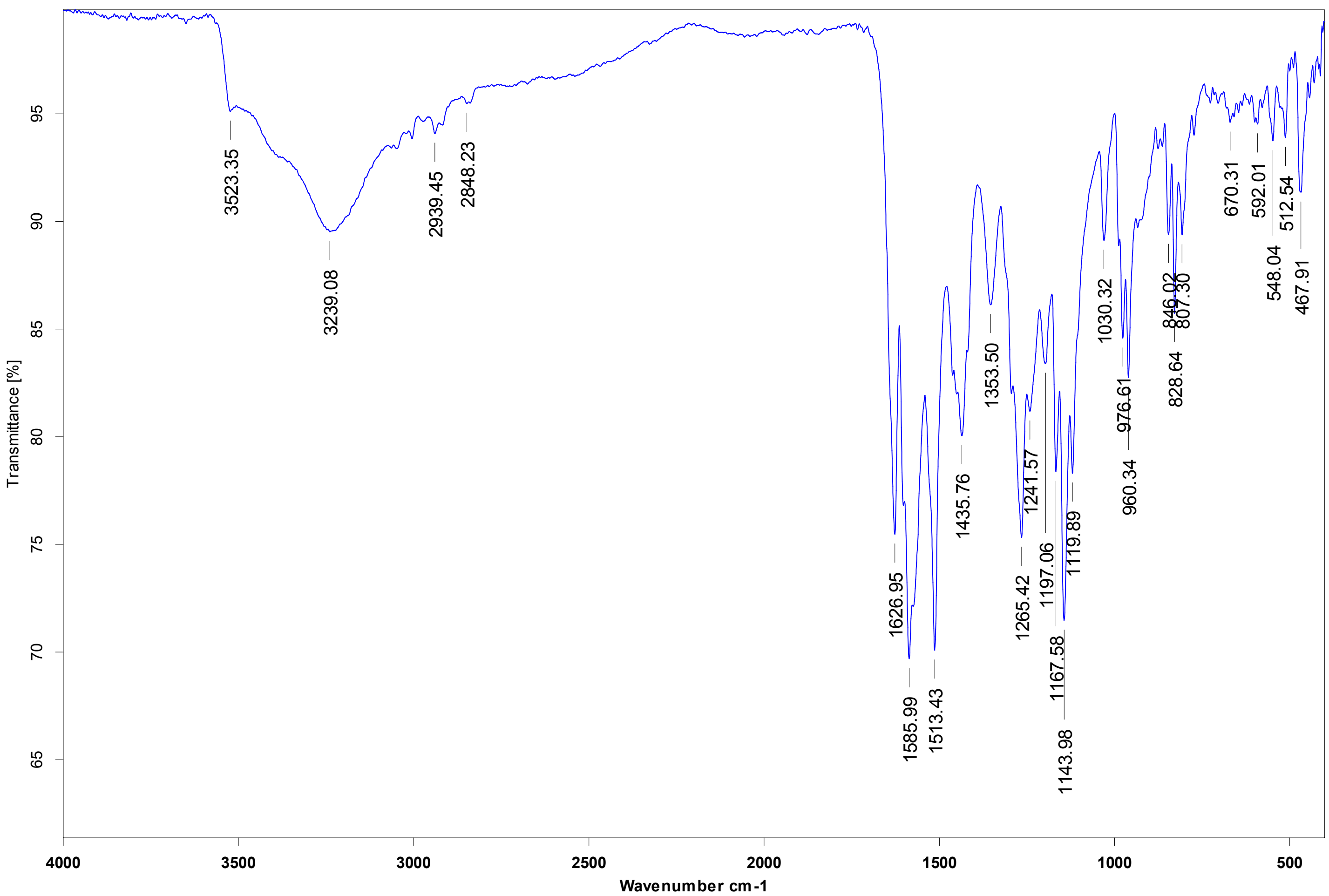

Figure S61. IR spectrum of curcuminoids C-3 from experimental condition iii).

Table S1. DPPH Inhibition (%) of pure compounds CUR, DMC and BDMC.

Sample	Concentration (μM)	D. O. (515 nm)	DPPH inhibition (%)	IC_{50} (μM)
CUR	Control	0.633±0.018		
	1	0.603±0.026	4.82±1.35	25.97±1.73
	1.78	0.608±0.017	3.87±1.27	
	3.16	0.577±0.022	8.82±1.28	
	5.62	0.539±0.021*	14.81±1.24*	
	10	0.482±0.021**	23.83±1.38**	
	17.78	0.394±0.020**	37.72±1.61**	
	31.62	0.274±0.018**	56.79±1.88**	
	56.23	0.137±0.010**	78.29±1.11**	
	100	0.048±0.002**	92.45±0.30**	
DMC	Control	0.633±0.018		
	1	0.626±0.020	0.98±0.74	46.04±1.70
	1.78	0.610±0.021	3.57±0.78	
	3.16	0.596±0.020	5.82±0.68	
	5.62	0.577±0.021	8.82±0.87	
	10	0.539±0.022*	14.91±1.31*	
	17.78	0.484±0.023**	23.6±1.61**	
	31.62	0.400±0.021**	36.81±1.52**	
	56.23	0.269±0.015**	57.51±1.56**	
	100	0.131±0.013**	79.38±1.42**	
BDMC	Control	0.648±0.013		
	1	0.650±0.011	-0.27±0.38	>100
	10	0.638±0.012	1.64±0.07	
	100	0.568±0.012**	12.41±0.12**	

α-tocopherol	Control	0.675±0.002		
	10	0.588±0.005*	12.86±0.85*	41.15±0.14
	17.78	0.529±0.005**	21.64±0.90**	
	31.62	0.420±0.001**	37.72±0.29**	
	56.23	0.219±0.002**	67.54±0.24**	
	100	0.034±0.001**	94.99±0.22**	
Quercetin	Control	0.675±0.002		
	3.16	0.560±0.004*	17.03±0.79*	10.87±0.40
	5.62	0.491±0.007**	27.14±1.24**	
	10	0.363±0.008**	46.21±1.29**	
	17.78	0.167±0.016**	75.17±2.40**	
	31.62	0.041±0.001**	93.87±0.19**	

All data were represented as mean ± standard error (SEM). Data were analyzed by one-way analysis of variance (ANOVA) followed by Dunnett's test for comparison against control. Values of $p \leq 0.05$ (*) and $p \leq 0.01$ (**) were considered statistically significant

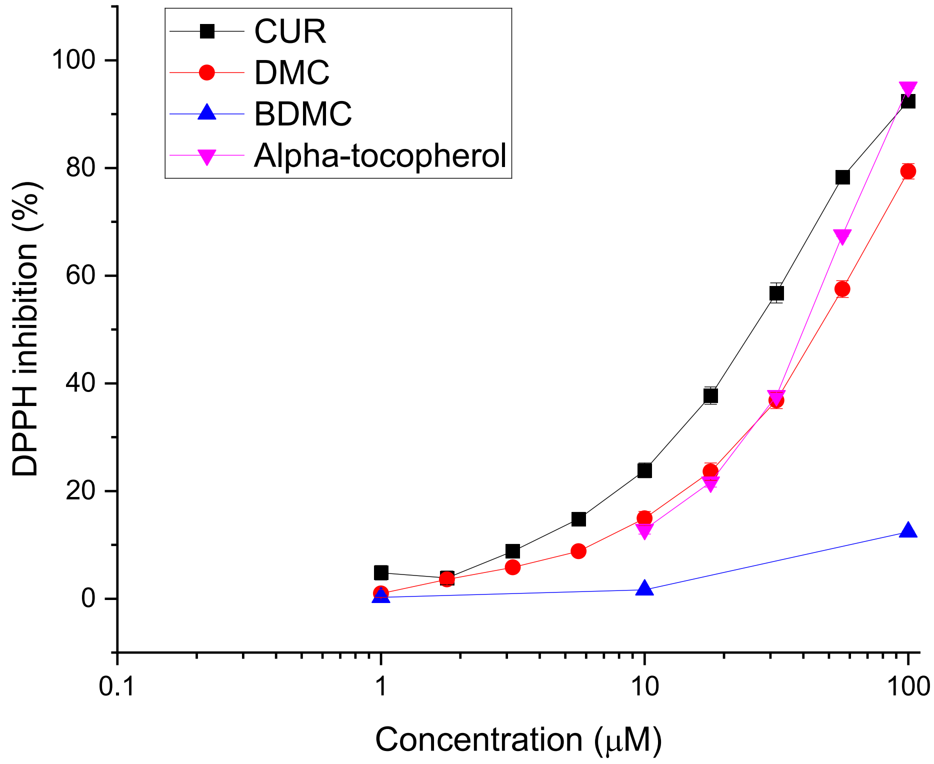

Figure S62. Plot dose-response DPPH Inhibition (%) of pure curcuminoids (CUR, DMC and BDMC).

Table S2. TBARS Inhibition (%) of pure compounds CUR, DMC and BDMC.

Sample	Concentration (μM)	TBARS (nmol/mg prot.)	Inhibition (%)	IC_{50} (μM)
CUR	Basal	0.33±0.02		
	Control	10.32±0.42		
	0.32	7.92±0.41**	23.33±1.12**	1.12±0.08
	1	5.50±0.47**	46.88±2.47**	
	3.16	1.48±0.38**	85.82±3.13**	
	10	0.38±0.04**	96.33±0.28**	
	31.62	0.30±0.02**	97.05±0.09**	
DMC	Basal	0.33±0.02		
	Control	10.32±0.42		
	0.32	8.44±0.39*	18.28±0.72*	1.51±0.19
	1	6.49±0.48**	37.28±2.32**	
	3.16	2.20±0.56**	78.94±4.74**	
	10	0.40±0.03**	96.1±0.35**	
	31.62	0.34±0.02**	96.74±0.17**	
BDMC	Basal	0.33±0.02		
	Control	10.32±0.42		
	0.32	9.39±0.45	9.07±0.62	3.71±0.14
	1	8.17±0.50**	20.97±1.95**	
	3.16	5.99±0.42**	42.05±1.82**	
	10	0.75±0.07**	92.72±0.42**	
	31.62	0.42±0.02**	95.98±0.06**	

All data were represented as mean ± standard error (SEM). Data were analyzed by one-way analysis of variance (ANOVA) followed by Dunnett's test for comparison against control. Values of $p \leq 0.05$ (*) and $p \leq 0.01$ (**) were considered statistically significant

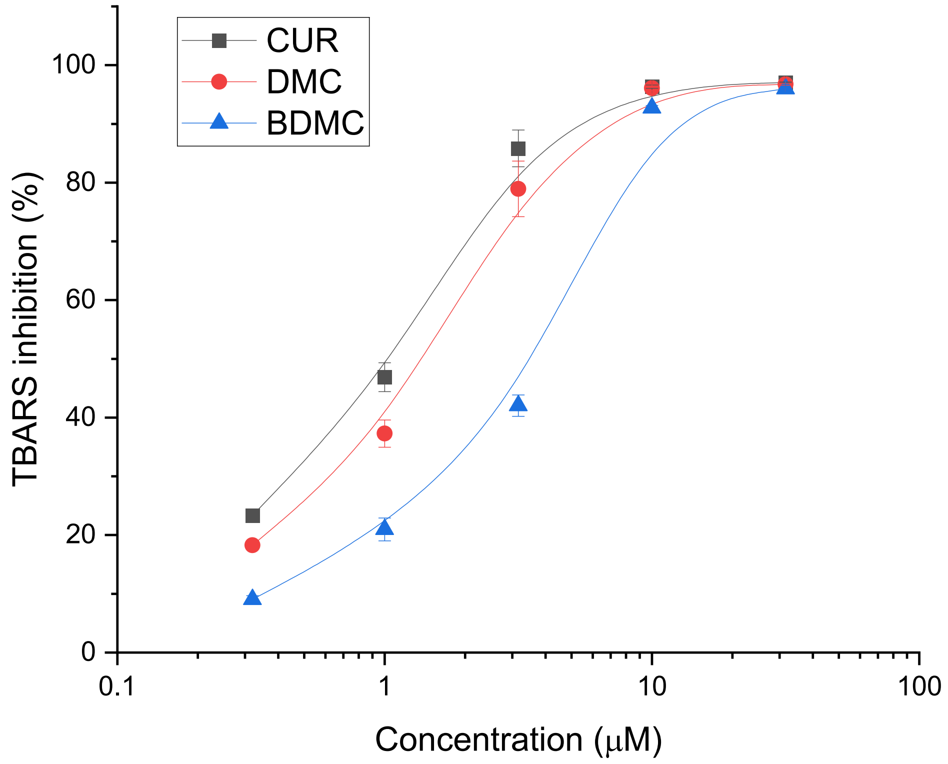

Figure S63. Plot dose-response TBARS Inhibition (%) of pure curcuminoids (CUR, DMC and BDMC).

Table S3. DPPH Inhibition (%) of C-3 mixtures.

Sample	Concentration (μg/mL)	D. O. (515 nm)	DPPH Inhibition (%)	IC_{50} (μg/mL)
Curcuminoids from turmeric	Control	0.625±0.023		
	1	0.584±0.020	6.66±0.93	8.75±0.17
	1.78	0.545±0.018**	12.92±0.74**	
	3.16	0.491±0.019**	21.45±0.25**	
	5.62	0.405±0.018**	35.31±0.68**	
	10	0.283±0.016**	54.71±0.97**	
	17.78	0.149±0.012**	76.2±1.05**	
	31.62	0.062±0.006**	90.11±0.61**	
	56.23	0.039±0.003**	93.86±0.26**	
	100	0.032±0.001**	94.79±0.11**	
C-3 Condition (i)	Control	0.651±0.003		
	1	0.615±0.003**	5.46±0.25**	13.19±0.13
	1.78	0.586±0.003**	9.92±0.24**	
	3.16	0.547±0.004**	15.90±0.46**	
	5.62	0.486±0.002**	25.25±0.56**	
	10	0.388±0.001**	40.29±0.36**	
	17.78	0.254±0.001**	61.00±0.37**	
	31.62	0.115±0.003**	82.40±0.39**	
	56.23	0.045±0.001**	93.03±0.13**	
	100	0.029±0.002**	95.49±0.29**	

C-3 Condition (ii)	Control	0.651±0.003		
	1	0.591±0.004**	9.15±0.41**	9.21±0.05
	1.78	0.564±0.000**	13.36±0.43**	
	3.16	0.512±0.002**	21.32±0.19**	
	5.62	0.424±0.004**	34.83±0.37**	
	10	0.311±0.003**	52.19±0.71**	
	17.78	0.159±0.001**	75.65±0.21**	
	31.62	0.060±0.001**	90.83±0.15**	
	56.23	0.030±0.001**	95.36±0.15**	
	100	0.029±0.002**	95.58±0.26**	
C-3 Condition (iii)	Control	0.625±0.023		
	1	0.596±0.020	4.69±0.57	13.17±0.24
	1.78	0.569±0.022	9.17±0.31	
	3.16	0.526±0.018**	15.84±0.68**	
	5.62	0.468±0.019**	25.17±0.56**	
	10	0.376±0.018**	39.96±0.85**	
	17.78	0.243±0.015**	61.18±0.91**	
	31.62	0.113±0.009**	82.07±0.86**	
	56.23	0.047±0.003**	92.57±0.21**	
	100	0.033±0.002**	94.79±0.32**	
α-tocopherol	4.31	0.588±0.005*	12.86±0.85*	
	7.66	0.529±0.005**	21.64±0.90**	17.72 ± 0.06
	13.62	0.420±0.001**	37.72±0.29**	
	24.22	0.219±0.002**	67.54±0.24**	
	43.07	0.034±0.001**	94.99±0.22**	

Quercetin	1.07	0.560±0.004*	17.03±0.79*	
	1.90	0.491±0.007**	27.14±1.24**	3.67±0.13
	3.38	0.363±0.008**	46.21±1.29**	
	6.01	0.167±0.016**	75.17±2.40**	
	10.69	0.041±0.001**	93.87±0.19**	

All data were represented as mean ± standard error (SEM). Data were analyzed by one-way analysis of variance (ANOVA) followed by Dunnett's test for comparison against control. Values of $p \leq 0.05$ (*) and $p \leq 0.01$ (**) were considered statistically significant

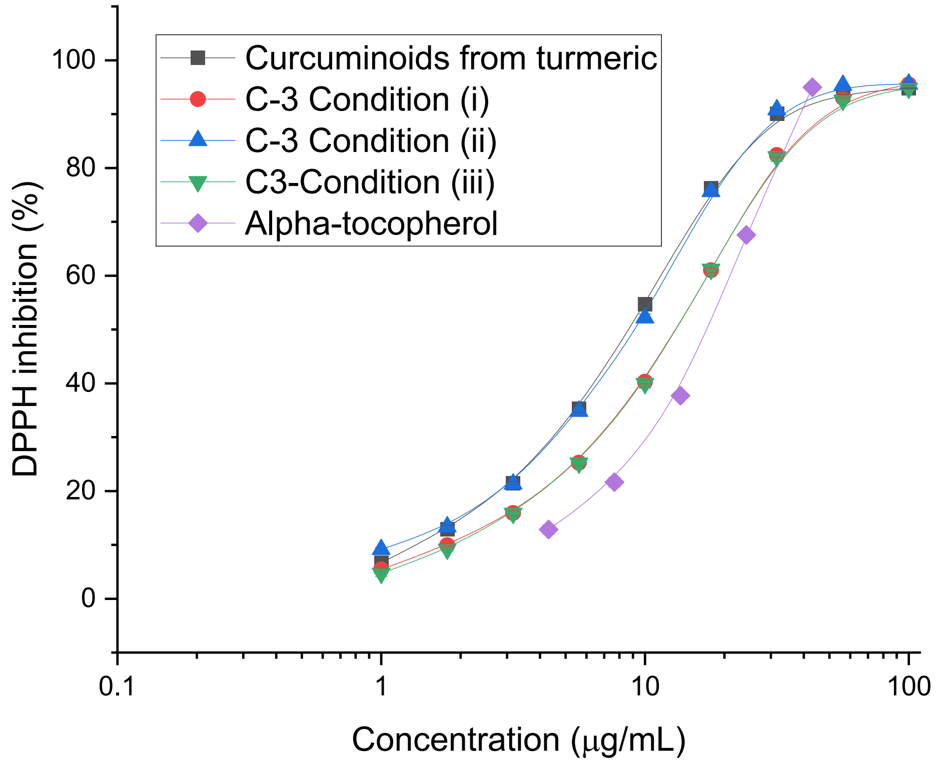

Figure S64. Plot dose-response DPPH Inhibition (%) of C-3 mixtures.

Table S4. TBARS Inhibition (%) of C-3 mixtures.

Sample	Concentration (μg/mL)	TBARS (nmol/mg prot.)	Inhibition (%)	IC_{50} (μg/mL)
Curcuminoids from turmeric	Basal	0.33±0.02		
	Control	10.32±0.42		
	0.32	7.80±0.36**	24.34±2.53**	1.24±0.04
	1	5.83±0.17**	43.39±1.07**	
	3.16	1.38±0.12**	86.65±0.90**	
	10	0.40±0.05**	96.17±0.36**	
	31.62	0.35±0.05**	96.63±0.55**	
C-3 Condition (i)	Basal	0.357±0.016		
	Control	11.450±0.887		
	0.1	10.550±0.625	7.58±1.65	1.87±0.11
	0.32	9.509±0.661	16.83±1.21	
	1	7.325±0.613**	36.07±0.81**	
	3.16	3.965±0.511**	65.52±2.76**	
	10	0.369±0.011**	96.73±0.32**	
C-3 Condition (ii)	Basal	0.357±0.016		
	Control	11.450±0.887		
	0.1	9.916±0.391	12.88±3.53	1.43±0.10
	0.32	8.613±0.332**	24.23±4.07**	
	1	6.609±0.331**	41.95±2.86**	
	3.16	3.147±0.308**	72.5±1.89**	
	10	0.556±0.134**	95±1.44**	
C-3 Condition (iii)	Basal	0.37±0.03		
	Control	10.30±0.43		
	0.32	8.36±0.28**	18.83±0.66**	1.50±0.02

1	6.28±0.23**	39.00±0.32**
3.16	2.72±0.08**	73.59±0.70**
10	0.44±0.03**	95.76±0.23**
31.62	0.38±0.03**	96.31±0.23**

All data were represented as mean ± standard error (SEM). Data were analyzed by one-way analysis of variance (ANOVA) followed by Dunnett's test for comparison against control. Values of $p \leq 0.05$ (*) and $p \leq 0.01$ (**) were considered statistically significant

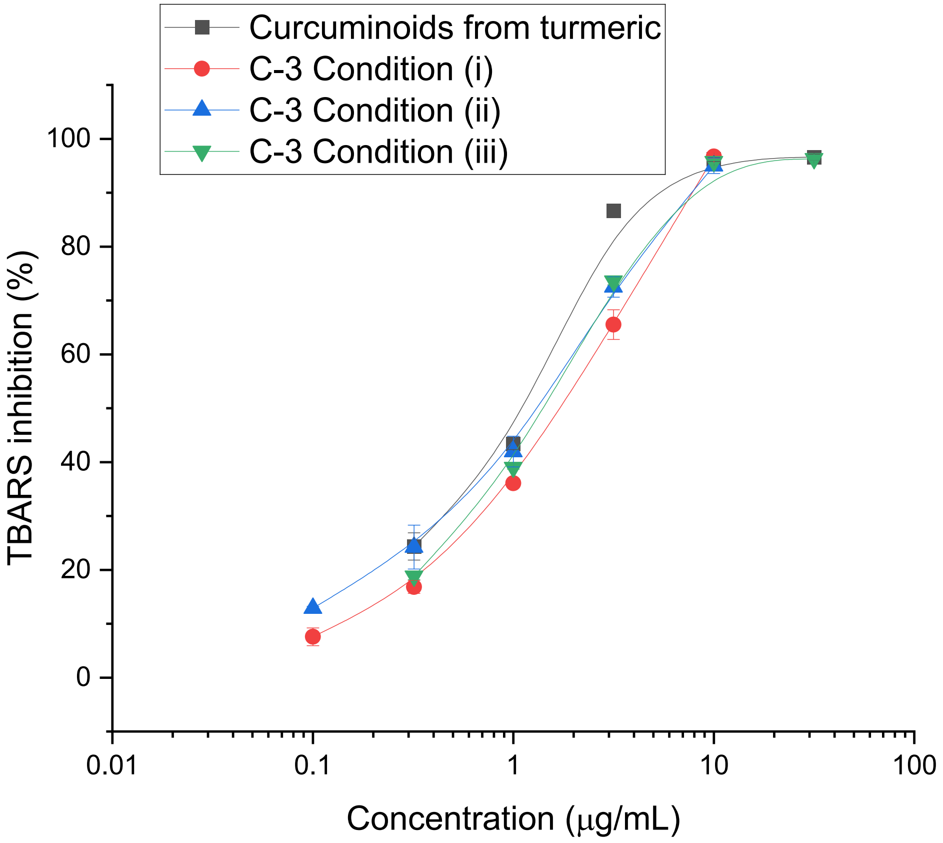

Figure S65. Plot dose-response TBARS Inhibition (%) of C-3 mixtures.
